# Monodentate Phosphine Modulation in Cyclometallated Platinum(II) Complexes for Antileishmanial, Antiviral, and Antitumor Applications

**DOI:** 10.1002/cmdc.202500782

**Published:** 2025-12-24

**Authors:** Antonio A. de Oliveira‐Neto, Gustavo Clauss, Jennyfer Castro, Marcus S. A. Garcia, Natasha M. Cassani, Bruna C. Sandim, Ana Laura C. Oliveira, Stephanie P. B. Reyes, Nádija N. P. da Silva, Fillipe V. Rocha, Ana C. G. Jardim, Danilo C. Miguel, Camilla Abbehausen

**Affiliations:** ^1^ Institute of Chemistry University of Campinas Campinas, 13083‐970 São Paulo Brazil; ^2^ Institute of Biology University of Campinas Rua Monteiro Lobato, 255 Campinas 13083‐970 São Paulo Brazil; ^3^ Laboratory of Antiviral Research (LAPAV) Institute of Biomedical Sciences Federal University of Uberlândia João naves de Ávila Avenue, 2121 Uberlândia 38408‐100 Minas Gerais Brazil; ^4^ Department of Chemistry Federal University of São Carlos Rodovia Washington Luis São Carlos 13565‐905 São Paulo Brazil

**Keywords:** antileishmanial, antivirals, neglected diseases, organometallic platinum complexes, phosphines

## Abstract

Complexes are emerging as promising alternatives for the treatment of neglected parasitic and viral infections, which urgently require new therapeutic strategies due to limited effective drugs. In this study, a series of [Pt(II)(phpy)(PR_3_)Cl] complexes, where phpy is 2‐phenylpyridine, and PR_3_ represents triphenylphosphine (PPh_3_), 1,3,5‐triaza‐7‐phosphaadamantane (PTA), para‐benzoic acid‐diphenylphosphine (PPh_2_(Ph*p*‐COOH), or tris(2‐carboxyethyl)phosphine (TCEP), are synthesized and systematically evaluated for their chemical properties and in vitro biological activities. Chemical reactivity, including ligand exchange with L‐histidine and *N*‐acetylcysteine, hydrophilic/lipophilic balance, and interactions with bovine serum albumin (BSA) and DNA, was correlated with biological outcomes. The novel TCEP complex exhibited exceptional chloride stability and intrinsic fluorescence but lacked antiviral and antileishmanial activity. The PTA derivative showed selective antileishmanial activity, achieving a selectivity index (SI) of 10.8 and reducing the infectivity index by 40% at 12 µM. Also, PTA showed selective antitumor activity in ovarian cancer (SI 9.1). In contrast, the PPh_2_(Ph*p*‐COOH) derivative demonstrated significant antiviral activity, inhibiting Mayaro virus and Zika virus replication by 94% and 78%, respectively, at 50 µM. These findings underscore the potential of coordination chemistry to fine‐tune biological activity and support the rational design of metal‐based therapeutics for neglected diseases.

## Graphical abstract

Schematic representation depicting the influence of phosphine ligand identity on the biological and physicochemical characteristics of Pt(II) complexes: PTA promotes antileishmanial activity, PPh_2_(Ph*p*–COOH) strengthens antiviral activity, and TCEP enhances fluorescence.

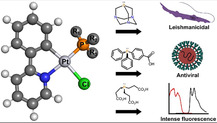

## 1 Introduction

Neglected tropical diseases (NTDs) affect more than one billion people worldwide, particularly in low‐ and middle‐income countries, and represent significant public health concerns. Among the NTDs, leishmaniasis, and arboviral infections, such as those caused by Zika and Mayaro viruses, are highlighted in this work.^[^ ^1^, ^2^, ^3^ ^]^ Leishmaniasis is a parasitic infection caused by the protozoa *Leishmania*, affecting around 1 million individuals across 98 countries per year, and is caused by at least 20 species of the protozoa. Transmission occurs via the bite of phlebotomine sandflies harboring the extracellular promastigote stage of the parasite, which is subsequently introduced into mammalian hosts, including humans, as well as domestic and wild animals. Within the mammalian host, the promastigote stage differentiates into the intracellular amastigote form, which is responsible for the pathogenesis and progression of the disease.^[^ ^1^ ^,^ ^3^, ^4^, ^5^ ^]^ Leishmaniasis is categorized into cutaneous, mucocutaneous, and visceral forms, with the latter affecting internal organs and posing a potentially fatal risk if left untreated. *Leishmania amazonensis* and *L. braziliensis* are predominantly associated with cutaneous leishmaniasis, particularly in Brazil.^[^ ^1^ ^,^ ^4^ ^,^ ^6^ ^]^ Currently, no vaccines are available for human use, and the existing chemotherapeutic regimens for leishmaniasis, including sodium stibogluconate, meglumine antimoniate, pentamidine, amphotericin B, miltefosine, and paromomycin sulfate, are often limited by their considerable toxicity, increasing resistance rate, and the requirement for intravenous or intramuscular administration. Although liposomal formulations of amphotericin B exhibit reduced toxicity, they remain associated with high costs and do not demonstrate consistent efficacy across all clinical manifestations of leishmaniasis.^[^ ^1^ ^,^ ^4^, ^5^, ^6^ ^]^

Another NTD is the arbovirus infections transmitted to humans through the bites of infected mosquitoes, with Zika virus (ZIKV), Mayaro virus (MAYV), Chikungunya virus (CHKV), and Dengue being notable examples.^[^ ^2^ ^,^ ^7^ ^,^ ^8^ ^]^ Arboviruses are responsible for over 40,000 deaths annually and impose a substantial economic burden on public health systems. The main clinical manifestations of arboviral infections include rash, fever, fatigue, headache, arthralgia, and conjunctivitis. Notably, maternal infection with ZIKV has been linked to the occurrence of microcephaly in newborns. In some cases of CHKV and MAYV, the infection may progress to a chronic arthritogenic disease, with symptoms persisting for months after the initial infection. To date, no vaccines or efficacious primary therapies have been approved for the prevention or treatment of arboviral infection.^[^ ^2^ ^,^ ^7^, ^8^, ^9^, ^10^, ^11^ ^]^

NTDs in general require attention from the scientific community and alternative therapies. In this context, metal complexes present the possibility of an alternative mode of action, as these types of infections pose significant challenges due to their intracellular nature, thereby necessitating the design of complexes with high selectivity indices (SI), minimizing cytotoxicity toward mammalian cells while maximizing activity against pathogens or inhibition of viral replication. Therefore, the antiviral and antiparasitic activities of metal complexes are much less explored than their antitumoral effects. The topics were systematically reviewed.^[^ ^4^ ^,^ ^12^, ^13^, ^14^, ^15^, ^16^, ^17^ ^]^ In this context, the stability of metal complexes is a critical factor, as they must traverse multiple cellular membranes and remain intact in various biological environments to reach their targets with minimal off‐target effects.^[^ ^4^ ^,^ ^16^, ^17^, ^18^, ^19^, ^20^, ^21^, ^22^ ^]^

Our research group has been investigating the role of phosphine and *N*‐heterocyclic carbenes (NHC) in the development of metal‐based inhibitors targeting zinc finger motifs, as well as in antiparasitic and antiviral applications.^[^ ^1^ ^,^ ^3^ ^,^ ^23^, ^24^, ^25^ ^]^ In our efforts, great attention was taken to Au(I) and Cu(I) bearing NHC and phosphine, but platinum(II) can offer high ligand inertness, especially with organometallic bonds.

Platinum has played a central role in the development of metal‐based pharmaceuticals, particularly since the clinical success of Pt(II) based anticancer agents such as cisplatin, carboplatin, and oxaliplatin.^[^ ^26^ ^]^ Over the past six decades, these complexes have become integral to standard chemotherapy protocols, especially for testicular, ovarian, head and neck cancers, and are frequently employed as second‐line treatments in other malignancies, including lung and skin cancers.^[^ ^27^ ^]^ In parallel, a wide variety of ligands have been explored to modulate the physicochemical and biological properties of these metal complexes. Among these, organometallic frameworks have gained prominence, with cyclometalated C^N Pt(II) complexes, particularly those incorporating 2‐phenylpyridine (phpy), receiving significant attention due to their catalytic activity and strong luminescence.^[^ ^28^ ^,^ ^29^ ^]^ These luminescent properties make such complexes attractive candidates for applications as bioprobes.^[^ ^30^ ^]^

Tertiary phosphines represent another well‐established class of ligands in metal‐based drug design.^[^ ^23^ ^,^ ^31^ ^,^ ^32^ ^]^ Their importance dates back to the development and approval of auranofin by Sutton and colleagues for the treatment of rheumatoid arthritis.^[^ ^33^ ^]^ Auranofin analogs have demonstrated a correlation between the chemical nature of the phosphine ligand and its bioavailability.^[^ ^34^, ^35^, ^36^ ^]^ Subsequent studies by Mirabelli and coworkers, as well as Berners–Price and colleagues, further substantiated the relationship between phosphine characteristics and the pharmacological behavior of gold complexes.^[^ ^37^ ^,^ ^38^ ^]^

In recent studies, Pt(II) complexes of the general formula [Pt(phpy)(PR_3_)Cl], where PR_3_ represents either triphenylphosphine (PPh_3_) or 1,3,5‐triaza‐7‐phosphaadamantane (PTA), have been evaluated for their anticancer activity.^[^ ^39^ ^,^ ^40^ ^]^ These complexes exhibited in vitro cytotoxicity against leukemia, lymphocyte, nonsmall cell lung carcinoma, and colorectal cancer cell lines, with IC_50_ values ranging from 0.8 to 24 µM. Notably, complexes with PTA showed enhanced activity. In a separate study, Solomatina and coworkers demonstrated the utility of these complexes as fluorescent bioprobes, showing that substitution of the chloride ligand with Histidine residues led to fluorescence activation.^[^ ^30^ ^,^ ^41^ ^,^ ^42^ ^]^

In the present study, we report the study of five Pt(II) complexes of the type [Pt(phpy)(PR_3_)Cl] for the design of antiparasitic and antiviral drugs. We evaluated the effect of the phosphine on chemical characteristics such as lipophilicity, ligand exchange reactions, albumin, and DNA interactions. The results were correlated with in vitro antiparasitic and antiviral activities.

## 2 Results

### 2.1 Synthesis and Characterization

**Figure 1:**
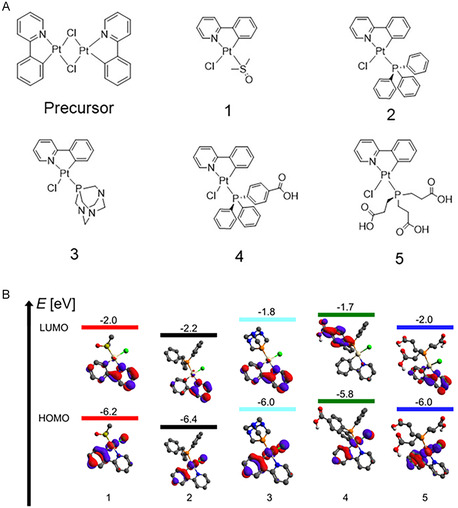
A) Structure of the synthesized complexes. B) HOMO and LUMO (eV) for Complexes 1–5.

The synthesis of the N^C cycloplatinated complexes followed the traditional route, which reacts the K_2_[PtCl_4_] complex salt with phenylpyridine, forming the dimer bearing two µ‐Cl, isolated as a greenish powder (**Figure** **1**A).^[^ ^29^ ^]^ Adaptations of volumes, time, and temperature had to be performed and are described in the experimental section. This dimer was used as a precursor, which forms 1 by dissolving it in DMSO and precipitating it as a green solid by the addition of water.^[^ ^43^ ^]^ From 1, DMSO can be exchanged by phosphines using an adequate solvent according to the phosphine solubility as described in the experimental section and in other articles in the literature.^[^ ^39^ ^,^ ^41^ ^]^ Among the five complexes, only 5 was not previously described. Single crystal was not obtained after testing different solvent systems. The complexes 1–5 possess different degrees of solubility in DMSO and CH_2_Cl_2_. The complexes are insoluble in water at a concentration of 1 mg mL^–1^.

Elemental analysis, electronspray ionization mass spectrometry (ESI‐MS), and ^1^H and ^13^C nuclear magnetic resonance spectroscopy (NMR) confirmed that complexes 1–5 were formed and are pure. The ^1^H, ^13^C, and ^31^P NMR, Fourier transformed infrared spectroscopy (FTIR), Raman spectra, and mass spectrometry are provided in the Supporting Information (Figure S1–S7, Supporting Information) and are in agreement with the literature.^[^ ^39^ ^,^ ^41^ ^,^ ^43^, ^44^, ^45^ ^]^ Theoretical calculations of the vibrational frequencies of the bonds in the five complexes were conducted. These computational findings, in combination with previously reported literature data, facilitated the assignment of the bands observed in the IR, far‐IR, and Raman spectra. For complex 5, the coordination is confirmed by ^1^H NMR, by the shifts in the CH_2_ groups, and by ^31^P NMR, which shows a singlet in 12.13 ppm in contrast to the signal of the free TCEP in 15.86 ppm at D_2_O. (Figure S3, Supporting Information) The singlet in 12.13 ppm has two antenna signals due to the nuclear coupling between ^195^Pt‐^31^P. ^1^H NMR spectra of 4 in DMSO‐d^6^ show a signal at 13.21 ppm assigned to the carboxylic acid proton of the benzoic group. Its integral is 1, showing it is totally protonated in DMSO‐d^6^ (Figure S1, Supporting Information). A similar analysis is relevant for 5, which shows a peak at 12.36 ppm assigned to the 3 protons of the tris‐carboxylic acid. The integral of this signal is 2, demonstrating the deprotonation of one unit in DMSO.

Thermogravimetric analysis indicated that all five complexes are thermally stable up to 124 °C. The steps in the weight loss process, related to the ligands, were well defined and showed errors within a margin of at most 5%. The phosphine derivatives are more thermally stable than the precursor. Complexes 2–5 begin decomposition in the range of 147–250 °C, whereas Complex 1 starts losing DMSO at around 124 °C (Figure S8, Supporting Information).

Bond dissociation energy (BDE) values in kJ/mol are useful as they indicate the bond strength between Pt and Cl.^[^ ^46^ ^]^ The Pt—Cl bond strength of the complexes containing phosphine derivatives follows the order 4 < 2 < 5 < 3 (**Table** **1**). The order seems to be influenced by the phosphorus pKa in the phosphine, which follows the same order (PPh_2_(Ph*p*‐COOH) < Ph_3_P < TCEP < PTA).^[^ ^25^ ^,^ ^47^, ^48^, ^49^, ^50^ ^]^ This order is confirmed by the magnitude of the ^1^J Pt–P coupling, which increases as the basicity of the phosphine decreases^[^ ^47^ ^]^ (Table 1).

**Table 1:** BDE, kJ mol^–^^1^ of the Pt—Cl bond for the five complexes; HOMO–LUMO energy gap (in eV) for the five complexes; Gibbs free energy change (ΔG in kJ mol^–1^) for the exchange reactions with Nac, His and DMSO for the five complexes; coupling ^1^J_pt–P_ in the ^31^P NMR spectra of complexes 2–5 (121.50 MHz; DMSO‐d^6^).

Complex	BDE [kJ mol^–1^]	GAP [eV] HOMO‐LUMO	ΔG [kJ mol^–1^] Nac (Theoretical)	ΔG [kJ mol^–1^] His (Theoretical)	ΔG [kJ mol^–1^] DMSO (Theoretical)	^1^J_Pt‐P_ MHz
**1**	436.7	4.19	77.4	−26.2	109.2	–
**2**	414.8	4.10	75.80	21.7	82.6	5334
**3**	427.8	4.13	75.1	25.7	88.1	4728
**4**	407.8	4.03	77.9	19.8	83.2	5352
**5**	418	4.22	82.2	35.4	88.8	4965

The electronic spectra of the complexes are similar. The electronic spectra present two bands around 256 and 285 nm, assigned to the *π* → π* transitions of phpy and aromatic phosphines, and three metal‐ligand charge transfer (MLCT) bands at around 315, 325, and 375 nm with small variation among the complexes (Figure S9, Supporting Information). These values are similar to those reported in literature,^[^ ^28^ ^,^ ^51^, ^52^, ^53^ ^]^ as there are no experimental data available in the literature for the novel complex 5, its spectral features were compared with time‐dependent density functional theory (TD‐DFT) calculations, which revealed highest occupied molecular orbital (HOMO) to lowest unoccupied molecular orbital (LUMO) transition exhibiting the same profile observed for the other complexes, corresponding to the dPt/*π*Cl/*π*phpy → π*phpy transition.

### 2.2 Ligand Exchange Reaction in DMSO

Speciation is a relevant topic in bioinorganic chemistry. DMSO can coordinate to metals and promote ligand exchange reactions, initiating speciation in solution. To evaluate the lability of chloride or phosphine ligands in DMSO, we prepared solutions of the complexes in CDCl_3_/DMSO with a complex‐to‐DMSO molar ratio of 1:10 and recorded ^1^H NMR spectra at 24 and 48 h. The coordinated DMSO presents a singlet at 3.65 ppm, while the uncoordinated at 2.62 ppm. In the analysis of Complex 1, this exchange is observed through an increase in the relative integral at 3.65 ppm in the ^1^H NMR spectra, while for Complexes 2–4, it is observed by the appearance of a peak near this value in the ^1^H NMR spectra. This exchange was only observed in the analyses of Complexes 1 and 3 after 48 h, with the proportions between the species containing two coordinated DMSO molecules and Complex 1, and the species containing one DMSO molecule and Complex 3 being 9.8% and less than 1%, respectively. No change in the ^31^P signal was seen after 48 h. No significant changes were observed in the ^1^H and ^31^P NMR spectra of Complex 5 in DMSO‐d^6^ over intervals of 24 and 48 h. (Figure S10, Supporting Information). DFT analyses of the exchange reaction, using DMSO as an implicit solvent, resulted in positive Gibbs free energy variations for all complexes, suggesting the exchange reactions are thermodynamically unfavorable (Table 1).

### 2.3 Interaction with N‐Acetyl‐L‐Cysteine (Nac) and L‐Histidine (His)

Nac is an excellent probe to follow ligand exchange reactions between metal complexes and thiolated biomolecules.^[^ ^54^ ^]^ Platinum complexes are usually prone to ligand exchange with sulfur donor molecules, which might affect the biological outcome.^[^ ^41^ ^,^ ^55^ ^]^ The reaction of the 1–5 with different concentrations of Nac was followed by UV–vis. The MLCT band around 375 nm is shifted to around 425 nm when a ligand exchange reaction occurs.^[^ ^28^ ^]^ The reactions were performed in a highly excess of Nac, starting in a molar ratio complex:Nac of 1:20. The increments in Nac concentration were by 20 equivalents, and the final point had a molar ratio of 1:220. The titration curves are presented in Figure S11–S12, Supporting Information. After the addition of a large excess of Nac, 220 molar equivalents, the ligand exchange in complex 2 reached 41.8% conversion. This result suggests the ligand exchange with thiol‐rich molecules is not thermodynamically favored. Theoretical calculations of the Gibbs free energy change (ΔG) associated with the ligand exchange of chloride by Nac were performed for each complex. (Table 1), indicating the exchanges are thermodynamically unfavorable for all complexes.

^1^H NMR spectroscopy was employed for 1, and both ^1^H and ^31^P NMR spectroscopy were used for 2–5 to investigate their interactions with His. The ^31^P NMR analysis of 5 revealed no significant changes between the spectra of the free complex and that obtained in the presence of His, indicating undetectable interaction under the experimental conditions used. In contrast, 2–4 exhibited marked changes in their ^31^P NMR spectra after the addition of His, indicating ligand exchange between chloride and His. These changes were characterized by the appearance of three new ^31^P resonance signals for each complex in DMSO‐d^6^/D_2_O (5/1): 22.20, 22.06, and 21.98 ppm for complex 2, whose free complex signal in DMSO‐d^6^ is 23.42 ppm; –64.68, –65.42, and –65.47 ppm for complex 3, whose free complex signal in DMSO‐d^6^ is –66.99 ppm; 22.25, 22.14, and 22.06 ppm for complex 4, whose free complex signal in DMSO‐d^6^ is 23.82 ppm (Figure S13, Supporting Information). The signal for the free complex 5 in DMSO‐d^6^ appears at 12.14 ppm, and after interaction with 15 equivalents of His in DMSO‐d^6^/D_2_O (5/1), it shifted to 12.69 ppm. The three signals observed for 2–4 can be attributed to the formation of different conformers or isomers of coordinated His that display isomerism associated with tautomerism of the imidazole ring, which may result in multiple conformations with distinct orientations relative to the cyclometalated moiety, as previously demonstrated.^[^ ^42^ ^]^

The ^1^H NMR signals around 3.30 and 3.40 ppm are characteristic of chloride substitution by His. These signals were observed in the spectra of complexes 2, 3, and 4. The absence of such signals in the spectra of 5 is consistent with the ^31^P NMR data, confirming the lack of chloride‐to‐His exchange. In the case of 1, it is suggested that the DMSO exchanges preferentially, positioning the coordinated His *trans* to the nitrogen atom of the phpy ligand. The Gibbs free energy changes (ΔG) associated with the substitution of chloride and DMSO by His in complex 1 were calculated. The former reaction was endergonic, with a ΔG of 19.8 kJ mol^–1^, whereas the latter was exergonic, with a ΔG of –26.2 kJ mol^–^^1^, indicating a more favorable outcome. Additionally, theoretical calculations of the Gibbs free energy change (ΔG) for the substitution of the chloride ligand by His in complexes 2–5 were performed (Table 1).

The LCMS analysis performed on a mixture of Complex 3 and His at a molar ratio of 1:15, confirmed the formation of [Pt(phpy)(PTA)(His)]^+^ by the signals m/z 661.1760 and 661.1754 and retention times of 8.24 and 9.45 min, respectively. Fragmentation (MS^2^) of the molecular ions at 661.1760 and 661.1754 m/z showed the signal m/z 506.1066, assigned to [Complex 3–Cl]^+^. The 155.0694 difference indicates the loss of one His molecule (Figure S14, Supporting Information). Calculations showed that the energy difference between the two Complex 3 + His isomers is 3.5 kJ mol^–^^1^, suggesting that the two signals are complex isomers.

The DFT calculations for the ligand exchange reaction of Complex 3 with His and Nac are 25.7 and 75.1 kJ mol^–^^1^, respectively (Table 1). Based on these results, it can be concluded that Complex 3, as well as the other four complexes, reacts preferentially with His rather than with Nac. However, it is important to highlight that, except for the substitution of DMSO by His in Complex 1, all these reactions are endergonic and, therefore, thermodynamically unfavorable.

Although theoretical and experimental data did not allow for a clear correlation between the Pt—Cl bond strength and the chloride ligand exchange in complexes 1–5 with Nac and His, it was observed that an increase in the HOMO–LUMO energy gap (eV) (Table 1 and Figure 1B) is associated with a lower tendency for ligand substitution. Specifically, complexes exhibiting a larger HOMO–LUMO gap demonstrate a less favorable chloride exchange with Nac (complexes 1–5) and with His (complexes 2–5), a finding that is corroborated by experimental evidence. A similar finding has been reported in the literature, where Solomatina and coworkers demonstrated that the substitution of the chloride ligand by imidazole is more favorable in platinum organometallic complexes containing aromatic phosphine ligands, rather than aliphatic ones.^[^ ^41^ ^]^

### 2.4 Significant Variation in Log P Can Affect Biological Effects

The octanol/water partition coefficient (log P) is a critical parameter for drugs, as it provides valuable information on the distribution tendency of the studied complexes between physiological polar and nonpolar environments. The balance of hydrophilic and lipophilic is crucial for their pharmacological efficacy.^[^ ^1^ ^]^ Complexes 1–5 were subjected to the octanol/water shake‐flask methodology (according to the OECD Guidelines for the Testing of Chemicals),^[^ ^56^ ^]^ and the concentration in the octanol phase was measured by UV–vis spectroscopy through the MLCT band, while the aqueous phase concentration was determined indirectly. The addition of DMSO was necessary for solubility reasons during the preparation of stock solutions: 5% (v/v) for complexes 1, 4, and 5, and 15% (v/v) for complexes 2 and 3. It is important to note that after the addition of water saturated with 1‐octanol during mechanical shaking experiments, the DMSO content was 2.5% for complexes 1, 4, and 5, and 7.5% for complexes 2 and 3. Although this certainly affects the log *p* values and may represent a possible source of error, the test still allows for a meaningful comparison among the complexes. The calibration curve was previously determined in octanol pre‐saturated with water, using the specific DMSO concentration required for each complex (Figure S15, Supporting Information). All complexes showed positive log *p* values, demonstrating their lipophilicity (**Table** **2**). The most hydrophilic complexes are 5 and 3, due to the hydrophilic groups present in the phosphine ligand. The most lipophilic complex is 2.

**Table 2:** Complexes 1–5 interactions with BSA measured by quenching of intrinsic fluorescence emission of BSA and treated with Stern–Volmer equations, where *K*_SV_ is the Stern–Volmer constant, K_b_ is the binding constant, and *n* is the number of interaction sites. Competitive binding parameters between the EB–CT‐DNA adduct and complexes 1–5, determined from the steady‐state fluorescence quenching of the adduct in the presence of each complex, Stern–Volmer constants (*K*_SV_, M^−1^) and quenching rate constants (kq, M^−1^ s^−1^). The logarithmic partition coefficients (log P) of each complex were determined from measurements of their respective MLCT band absorbances.

Complex	BSA interaction			*DNA* interaction		*Log P*
	K_SV_ 10^4^ M^−1^	K_b_ 10^4^ M^−1^	*n*	K_SV_ 10^3^ M^−1^	kq 10^11^ M^−1^ s^−1^	
1	1.87 ± (0.07)	1.00 ± (0.10)	1.14 ± (0.03)	3.36 ± (0.15)	1.46 ± (0.06)	1.04 ± (0.09)
2	1.52 ± (0.04)	1.54 ± (0.15)	0.98 ± (0.03)	5.91 ± (0.14)	2.57 ± (0.06)	1.20 ± (0.02)
3	3.04 ± (0.10)	3.50 ± (0.61)	0.96 ± (0.05)	4.62 ± (0.10)	2.01 ± (0.04)	0.44 ± (0.02)
4	2.17 ± (0.09)	1.41 ± (0.18)	1.09 ± (0.04)	2.44 ± (0.15)	1.06 ± (0.06)	1.05 ± (0.01)
5	1.60 ± (0.06)	1.82 ± (0.30)	0.96 ± (0.05)	4.74 ± (0.18)	2.06 ± (0.08)	0.21 ± (0.03)

### 2.5 Serum Albumin

Albumins are serum proteins that play a crucial role in the transport of both exogenous and endogenous substances. Bovine serum albumin (BSA) has been more extensively employed than human serum albumin in studies assessing the binding affinity of potential drug candidates to serum albumin, primarily due to its high availability, lower cost, and the strong structural homology between the two proteins. BSA exhibits intrinsic fluorescence at ≈345 nm, arising from its two tryptophan residues, which undergo quenching upon interaction with metal complexes.^[^ ^1^ ^,^ ^57^ ^]^ It was first necessary to demonstrate that the complexes do not exhibit significant fluorescence emission in the 300–450 nm range when excited at 280 nm; their spectra were compared to that of BSA (Figure S16, Supporting Information). The complexes were tested for their interaction with BSA by measuring BSA intrinsic emission band (*λ*_em_ 350 nm, *λ*_exc_ 280 nm) in a mixture with different concentrations of 1–5. The emission subjected to Stern–Volmer treatment and the data are presented in Table 2. All the curves can be found in *Supporting Information* (Figure S17–S18, Supporting Information).

All complexes exhibit K_SV_ and Kb values on the order of 10^4^ (Table 2), indicating an efficient interaction with the protein, within the range that allows their transport, distribution, and release at the respective targets within the body.^[^ ^1^ ^,^ ^58^ ^,^ ^59^ ^]^ Complex 3 has a slightly higher constant. All the complexes do not present linear relations of F_0_/F versus complex concentration (K_SV_); the quadratic polynomial function is the one that best represents this complete graph. They present deviations in higher concentrations. To calculate K_SV_ and K_b_, we use the linear portion of the curves in lower concentrations. The linearity is kept up to complex: BSA molar ratio close to 0.5. This result suggests that static and dynamic quenching are happening for all the complexes in low concentrations of the complexes.^[^ ^60^, ^61^, ^62^, ^63^, ^64^, ^65^, ^66^ ^]^

### 2.6 The Ethidium Bromide Displacement Assay by Fluorescence Emission Suggests that the Five Complexes May Act as Intercalators with CT‐DNA

To investigate the potential intercalative interaction of the complexes with DNA, fluorescence spectroscopy measurements were performed based on the displacement of ethidium bromide. Ethidium bromide is a fluorescent phenanthridine dye that serves as a typical DNA intercalator. This property arises from the interaction between its planar phenanthridine ring and the stacked base pairs of CT‐DNA, leading to the formation of the EB–CT‐DNA adduct. It is noteworthy that neither ethidium bromide nor DNA exhibits fluorescence upon excitation at 540 nm; however, the EB–CT‐DNA adduct displays intense emission. Consequently, ethidium bromide is widely employed as an indirect probe for DNA intercalation, as the introduction of another intercalative complex into the EB–CT‐DNA system may result in the displacement of EB from the intercalation sites, thereby causing quenching of the EB–CT‐DNA fluorescence.^[^ ^67^, ^68^, ^69^ ^]^ First, the five complexes did not exhibit significant fluorescence, either in the presence or absence of CT‐DNA in buffer solution, within the 560–800 nm range when excited at 540 nm (Figure S19, Supporting Information). The K_SV_ and kq values for the interaction of each complex with DNA are shown in Table 2. The kq values are higher than 10^10 ^M^−1 ^s^−1^, indicating that the fluorescence quenching of the EB–CT‐DNA adduct induced by the complexes may occur through a static quenching mechanism, leading to the formation of a new Complex CT‐DNA adduct. From these data, it is possible to suggest a competition between each complex and EB for CT‐DNA; in other words, an intercalation between each complex and DNA may be indirectly suggested.^[^ ^67^ ^,^ ^70^ ^]^ The *K*_f_ value was obtained from Equation (2), where *K*_f_ = *K*_b_. The constant *K*_f_ represents the fluorescence binding constant, or apparent binding constant.^[^ ^68^ ^,^ ^69^ ^]^ The *K*_f_ values ranged from 4.79 × 10^3 ^M^−1^ to 3.10 × 10^4 ^ M^−1^ for the five complexes, whereas the corresponding *n* values, representing the number of binding sites per nucleotide, were found to vary between 0.61 and 0.98 for these complexes. The *K*_f_ and *n* values, together with the fluorescence spectra of the EB–CT‐DNA adduct in the presence of each complex, are provided in the Supporting Information (Figure S20–S21, Supporting Information). The arrows denote the fluorescence quenching of the EB–CT‐DNA system induced by the addition of each complex.

### 2.7 Complex 5 is Fluorescent in All Conditions and Complex 3 is Fluorescent in the Presence of His

**Figure 2:**
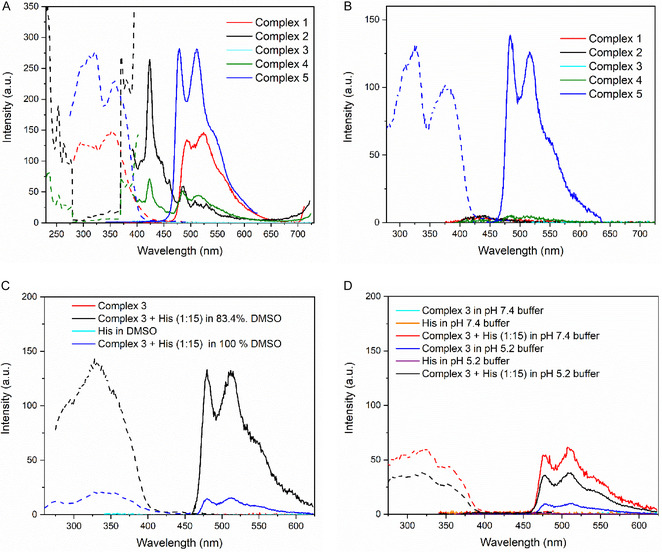
Excitation (dashed) and emission (solid) spectra of complexes in different conditions. A) Excitation spectra (*λ*_em_ = 524 nm for Complex 1, *λ*_em_ = 424 nm for Complex 2, *λ*_em_ = 423 nm for Complex 4 and *λ*_em_ = 511 nm for Complex 5) and emission spectra (*λ*_ex_ = 356 nm for Complex 1, *λ*_ex_ = 371 nm for Complex 2, *λ*_ex_ = 372 nm for Complexes 3–4, and *λ*_ex_ = 322 nm for 5) of 1–5 in Tris‐HCl 100 mM pH 7.4 containing 13.3% (v/v) DMSO (except 2 19.3%. (v/v) DMSO) at RT at 1,3 × 10^−4^ mol L^–^^1^. B) Excitation spectra (*λ*_em_ = 516 nm for Complex 5) and emission spectra (*λ*_ex_ = 356 nm for Complex 1, *λ*_ex_ = 371 nm for Complex 2, *λ*_ex_ = 372 nm for Complexes 3–4, and *λ*_ex_ = 322 nm for Complex 5) of 1–5 in DMSO) at RT at 1,3 × 10^−4^ mol L^–^^1^. C) Excitation spectra (*λ*_em_ = 510 nm for a mixture Complex 3 + His (1:15) in 83.4% DMSO and *λ*_em_ = 480 nm for a mixture Complex 3 + His (1:15) in 100% DMSO) and emission spectra (*λ*_ex_ = 321 nm for 3, His, a mixture Complex 3 + His (1:15) in 100% DMSO and 83.4% DMSO at RT at 1,1 × 10^−4^ mol L^–^^1^ for Complex 3. D) Excitation spectra (*λ*_em_ = 510 nm for a mixture Complex 3 + His (1:15) in Tri‐HCl 100 mM pH 7.4 and sodium acetate buffer 100 mM pH 5.2) and emission spectra (*λ*_ex_ = 321 for Complex 3, His, and a mixture Complex 3 + His in Tri‐HCl 100 mM pH 7.4 and sodium acetate buffer 100 mM pH 5.2 at RT at 1,1 × 10^−4^ mol L^–1^ for Complex 3.

The emission spectra of complexes 1–5 were obtained in 130 µM solutions both in 100 mM Tris‐HCl buffer at pH 7.4 containing 13.3% (v/v) DMSO for complexes 1 and 3–5, and 19.3% (v/v) DMSO for complex 2, due to solubility reasons (**Figure** **2**A) and in 100% DMSO (Figure 2B). All spectra were acquired at room temperature. The excitation wavelength was chosen for each complex by measuring emission spectra at all the absorption bands. After defining the main emission band, excitation spectra were acquired at this emission band to define the maximum excitation wavelength. However, for the fluorescence studies, the excitation bands of 371 and 372 nm were used for 2 and 4.

The complexes do not present emission bands in DMSO, except for Complex 5, which presents three emission bands, at 484 nm, 516, and a shoulder in 556 nm (Figure 2B). On the other hand, in Tris‐HCl pH 7.4, all complexes, except for 3, present emission (Figure 2A). The complexes 1, 2, 4, and 5 exhibit emission in the range of 450–600 nm with three emission bands detected close to 480, 510 nm, and a shoulder at 560 nm. In the fluorescence spectra of Complex 2, it is also possible to observe low‐intensity signals at 460 and 531 nm. For 2 and 4, these bands are lower, and a more intense band is observed around 425 nm. The complex 3 highlights the fact that no emission band was found for any excitation tested in both solvents. The results also show that emission is dependent on the solvent system. Previous reports were performed in DMSO or CH_2_Cl_2_, showing that the complexes 2–4 were not emissive.^[^ ^41^ ^,^ ^42^ ^]^ These findings indicate that the phosphine ligand plays a decisive role in modulating the fluorescence of the complexes, both in buffer at pH 7.4, which mimics physiological conditions, and in DMSO. A direct comparison of the fluorescence emission of all five complexes in Tris‐HCl buffer at pH 7.4 is not possible, as they contain different DMSO concentrations. However, it can be concluded that DMSO quenches the fluorescence of Complexes 1, 2, and 4, but not of Complex 5. Fluorescence constitutes a key physicochemical property, essential for enabling the application of metal complexes as biosensors or in bioimaging.^[^ ^30^ ^,^ ^41^ ^]^

A fluorescence emission kinetics analysis of the complexes was carried out in the presence of amino acids (aa) Nac, Cys, and His at a molar ratio of [aa]/[Complex] = 5 for 60 min. No significant changes in the emission were seen for 1–5 in the reaction with Nac and Cys. In the case of His, except for 3, the complexes did not turn on the emission. In the case of 3, which does not present any emission in the conditions tested, the emission turned on by the addition of 5 equivalents of His at the initial time points with no significant changes over time. An intense emission band at 478, 510 nm, and a shoulder are observed at 548 nm (*λ*_exc_ = 321 nm).

Solomatina and coworkers reported the emission of complexes 2–4 and other N^CPt(II) bound to several phosphines. When chloride is replaced by imidazole in DMSO or CH_2_Cl_2_ solution at room temperature, a strong emission is observed.^[^ ^41^ ^,^ ^42^ ^]^ The author also prepared 2 and reacted with several amino acids. Only the reaction of platinum ciclometallated complex with His was able to produce a strong emission spectra in DMSO.^[^ ^42^ ^]^

Inspired by this work and by our previous results, we reacted Complex 3 with His. Titrations were performed at molar ratios ranging from 1:5 to 1:55 of [Complex 3]/[His]. It was found that the maximum emission at 510 nm upon excitation at 321 nm does not show significant changes after the [His]/[Complex 3] ratio exceeds 1:15 (Figure S22, Supporting Information). Based on this result, a fluorescence analysis of Complex 3 + His at a 1:15 ratio was carried out, exciting at 321 nm in 100% and 83.4% DMSO, in 100 mM Tris‐HCl buffer at pH 7.4, and in 100 mM acetate buffer at pH 5.2 (Figure 2C,D).

For Complex 3, the emission in the range of 480–600 nm is activated with the addition of His in all conditions. The most intense emission is seen at an 83.4% DMSO solution. Interestingly, Complex 3 presents a low emission in this region at pH 5.2, which brought up the hypothesis that the protonation of PTA nitrogen groups could be activating the emission band. Aqueous solution in pH 5.2 (corrected with low amounts of HCl) of Complex 3 does not present emission bands, demonstrating the protonation is not the cause of activating emission (Figure S23, Supporting Information). It is noteworthy that the fluorescence intensity of the Complex 3 + His interaction remains unchanged across both acidic media, namely the buffer solution and the aqueous HCl solution. The set of results demonstrates Complex 3 has an emission band in the region of 480—510 nm (*λ*_exc_ 321 nm) when the chloride is replaced by His.

BSA also activates the fluorescence of Complex 3, with emission observed at a 1:1 molar ratio, pH 7.0, and excitation at 321 nm (Figure S24, Supporting Information). The emission peaks are observed at 478 nm and 510 nm, with a shoulder near 548 nm, similar to the profile of Complex 3 in the presence of His at a 1:15 molar ratio.

Attempts to visualize the complexes in cells were unsuccessful. Because excitation at the fluorescence maximum required high energy, imaging was performed using an excitation wavelength within the absorption band but below the emission maximum. Under these conditions, the complexes did not produce a detectable fluorescence signal, suggesting that their emission efficiency in the cellular environment is low or that the signal was below the detection limit for the equipment used.

### 2.8 Complexes Present Significant Antiviral Activity

The complexes were tested for the inhibition of viral replication of the Mayaro (MAYV) and Zika (ZIKV) viruses. First, cell viability in the host cell (Vero E6) was measured, and the concentration where viability was higher than 80% was chosen for measurement of inhibition of viral replication. As positive controls, we report EIDD‐2749 (molnupiravir prodrug), a nucleoside analog that inhibits viral RNA polymerase, with activity against alphaviruses, used in the evaluation of MAYV,^[^ ^71^ ^,^ ^72^ ^]^ and for ZIKV, we added Obatoclax (OLX), a Bcl‐2 protein modulator, with antiviral effects described against ZIKV, EBOV, and other RNA viruses.^[^ ^73^ ^,^ ^74^ ^]^ The concentration in µM and the % cell viability are reported in **Table** **3**. Among the cell viabilities, Complex 3 has the lowest concentration, showing it is more toxic for host cells than the others. Complex 1 was able to inhibit MAYV replication selectively, while Complex 3, although less potent, is also selective. In general, complexes were very potent against MAYV and showed lower potency against ZIKV, with Complex 4 presenting the highest inhibition rate on virus replication.

**Table 3:** Antiviral effects of the complexes in the highest cell viability (Vero E6) concentration.

Complex	Concentration [µM]	MAYV		ZIKV	
		Cell viability [%]	Inhibition of replication [%]	Cell viability [%]	Inhibition of replication [%]
1	50.00	107.0	95.50	108.6	0.00
2	50.00	101.4	90.60	101.3	26.10
3	10.00	102.3	76.60	79.20	4.70
4	50.00	94.50	93.90	97.80	78.30
5	50.00	98.50	0	100.0	15.60
OLX	1.000	–	–	100.0	99.20
EIDD2749	10.0	100.0	100.0	–	–

### 2.9 Complex 3 Presents Significant Leishmanicidal Activity

The inhibitory concentration 50% (IC_50_) of 1–5 was determined for *Leishmania amazonensis*, one of the causative agents of cutaneous leishmaniasis in the New World (**Table** **4**).^[^ ^75^ ^]^ The complexes were not active in relevant concentrations < 10.0 µM against the promastigote stage, except for the Complex 3, which demonstrated significant leishmanicidal activity. In this case, the IC_50_ in bone marrow differentiated macrophages (BMDM) was determined, showing a value of 34.5 µM. The values represent a selectivity index (SI) of 10.8, in accordance with the heteroleptic neutral Au(NHC) that we have been testing^[^ ^3^ ^]^ and higher than values found for heteroleptic neutral Cu(NHC) tested in the same conditions and species and the positive control Ambisome (AMB).^[^ ^1^ ^]^

**Table 4:** Inhibitory concentration at 50% (IC_50_) in *L.* *amazonen* *si* *s* promastigotes and at 50% in BMDM and SI calculated (SI) by IC_50_ (BMDM)/ IC_50_ (*L. amazonensis*).

Complex	*L. amazonensis* IC_50_ ± SD [µM]	BMDM IC_50_ [µM]	SI
1	>50.0	–	–
2	>25.0	–	–
3	3.02 ± 0.70	34.5	10.8
4	>50.0	–	–
5	>50.0	–	–
AMB^[^ ^6^ ^]^	0.53 ± 0.10	1.97 ± 0.29	3.71

**Figure 3:**
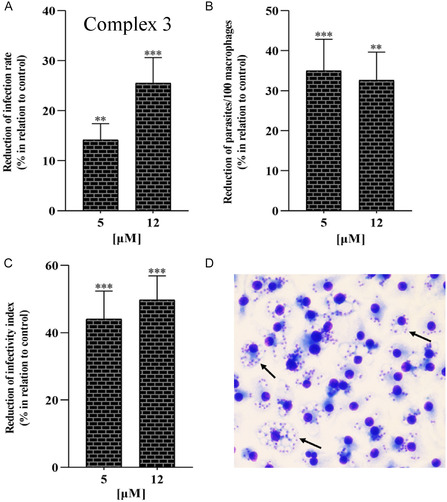
Reduction in macrophagic infection rates. BMDM were infected with promastigotes of *L. amazonensis* (5 parasites:1 BMDM), and infections were incubated with 5 and 12 µM of Complex 3 for 24 h. A) Reduction of the infection rate presented as a % in relation to control. B) Reduction of intracellular parasite number compared to untreated infections. C) Infectivity index (infection rate x parasites/100 macrophages) obtained for 3‐incubated cells in relation to untreated control infections. D) Representative image of infected BMDM. Arrows point to amastigotes; BMDM nucleus size ≈8 µm. **p < 0.005; ***p < 0.0005 (untreated control infections *versus* Complex 3‐incubated infections).

To further evaluate the leishmanicidal effects of Complex 3, infected BMDM were incubated with this complex at 5 and 12 µM for 24 h. After infection, promastigotes differentiate into amastigotes inside a parasitophorous vacuole. To evaluate the effects of Complex 3 on the amastigotes, BMDM is infected with promastigotes, generating infected BMDM (**Figure** **3**D). The infection rate is determined as the number of BMDM cells that are infected (control around 80%). The infected BMDM is treated with Complex 3, and the number of infected cells is determined again, giving an infection rate for the treated cells. Figure 3A reports the decrease in the infection rate caused by the Complex 3. Figure 3B reports the number of amastigotes/100 macrophages, and Figure 3C reports the infectivity index, which is the number of amastigotes per number of infected cells. Complex 3 was able to reduce the infection by around 35% and reduce the infectivity index by 45% at 12 µM when compared to untreated infections.

### 2.10 Viability in Cell Lines

Within the evaluated concentration range (0.39–25 µM), none of the tested complexes exhibited significant cytotoxicity toward MCF‐7 (breast cancer) or HaCaT (nontumor keratinocyte) cells, which prevented the determination of IC_50_ values for these cell lines. This result indicates low nonspecific toxicity under the experimental conditions (**Table** **5**). However, in the A2780 ovarian carcinoma cell line, complexes 1 and 3 exhibited significant cytotoxicity, with an IC_50_ value of 2.75 ± 0.3 µM and 1.39 ± 0.21 µM. Meanwhile, Complex 3 displayed much lower toxicity toward the nontumorigenic MRC‐5 fibroblast cell line, with an IC_50_ value of 17.4 ± 1 µM. This results in a SI greater than 9.10, highlighting its preferential activity against tumor cells. Conversely, Complex 1 exhibited activity against moderate toxicity toward MRC‐5 cells (IC_50_ = 5.06 ± 0.6 µM), resulting in a lower selectivity (SI = 3.64). However, both Complexes 1 and 3 showed higher selectivity indices than cisplatin. Complexes 2, 4, and 5 remained inactive across all evaluated cell lines. Overall, Complex 3 is the most promising candidate due to its significant and selective cytotoxicity toward ovarian cancer cells, as well as its limited impact on nontumorigenic cells. Further investigation into its mechanism of action and potential as an anticancer agent is warranted. These results suggest that the new complexes have a more favorable therapeutic profile and could be promising candidates for developing metal‐based anticancer agents with reduced off‐target toxicity.

**Table 5:** Inhibitory concentration at 50% (IC_50_) in vitro against MCF‐7, HaCaT, A2780, and MRC‐5 cells after 48 h treatment with 15.

Complex	MCF 7 IC_50_ [µM]	HaCaT IC_50_ [µM]	A2780 IC_50_ [µM]	MRC‐5 IC_50_ [µM]	SI
1	>25.0	>25.0	1.39 ± 0.21	5.06 ± 0.60	3.64
2	>25.0	>25.0	>25.0	>25	–
3	>25.0	>25.0	2.75 ± 0.30	17.4 ± 1.00	9.10
4	>25.0	>25.0	>25.0	>25	–
5	>25.0	>25.0	>25.0	>25	–
cisplatin	–	–	11.80 ± 0.80	29.09 ± 0.78	2.47

SI: IC_50_ (MRC‐5)/IC_50_ (A2780).

Given that Complex 3 exhibits the most potent antileishmanial and antitumor activity, and that the substitution of its chloride ligand by His is accompanied by an increase in fluorescence in buffers at pH 7.4 and 5.2, the latter simulating the environment of the parasitophorous vacuole, these findings may provide valuable insights for future investigations aimed at elucidating the biomolecular target of this complex, particularly if the reactive site contains His residues.^[^ ^6^ ^,^ ^30^ ^]^

## 3 Conclusion

The study of the [Pt(II)(phpy)(PR_3_)Cl] series revealed the significant influence of the phosphine ligand on both antiviral, leishmanicidal, and antitumoral activities. Among the complexes, the one coordinating TCEP (5) is novel. This complex exhibited the highest inertness within the series, showing negligible chloride exchange with amino acids and displaying increased hydrophilicity. Additionally, complex 5 demonstrated intrinsic fluorescence in the 480–550 nm range. Despite its interesting chemical properties, it showed no significant antitumor, antiviral, or antileishmania activity. In contrast, the PTA derivative (Complex 3) was the only complex in the series to exhibit leishmanicidal activity, with a promising SI. However, its antiviral potential is limited, primarily due to cytotoxicity in Vero‐E6 host cells, a limitation not observed in BMDM. Although Complexes 3 and 5 have similar log *p* values, Complex 3 lacks proton donor groups. We propose that the presence of multiple proton donor groups in Complex 5, along with its high chloride inertness, reduces its suitability for medicinal applications, despite its favorable hydrophilic/lipophilic balance. The most effective antiviral activity against arboviruses was observed with Complexes 1, 2, and 4. Complex 1 inhibited 96% of Mayaro virus (MAYV) replication at 50 µM but showed no significant effect against Zika virus (ZIKV). In contrast, Complex 4 inhibited MAYV and ZIKV replication by 94 and 78%, respectively, at the same concentration, representing the best balance of antiviral efficacy in the series. Regarding the antitumor effects, complexes 1 and 3 are active against ovarian tumor cells, but Complex 3 is more selective and offers a promising starting point for further investigations into its therapeutic potential. Overall, this study demonstrates that the [Pt(II)(phpy)(PR_3_)X] complexes can be modulated to fine‐tune selective biological activity by modifying ligands, offering a pathway to optimize antitumor, antiviral, and leishmanicidal properties.

## 4 Experimental Section

4.1

4.1.1

K_2_PtCl_4_, 2‐phenylpyridine (phpy), triphenylphosphine (Ph_3_P), p‐benzoic acid diphenylphosphine PPh_2_(Ph*p*‐COOH), 1,3,5‐triaza‐7‐phosphaadamantane (PTA), tris‐carboxyethylphosphine (TCEP), BSA, *N*‐acetylcysteine (Nac), *L*‐cysteine (Cys), and *L*‐Histidine (His) were purchased from Sigma–Aldrich and used as received. Calf‐thymus DNA (CT‐DNA) was purchased from Sigma–Aldrich. Ethidium bromide (EB) was purchased from Bio‐Rad Laboratories and stored in a refrigerator. Solvents and common salts are maintained in the laboratory from different suppliers. The reactions to obtain the Precursor and 5 were carried out under an argon atmosphere. Buffers, BSA, and DNA solutions were stored in the refrigerator. CHN elemental analysis was performed on the PerkinElmer 2400 Series II CHN Analyzer instrument. ^1^H, ^13^C, and ^31^P NMR were performed on the Bruker Avance III HD 250 MHz, Bruker Avance II + 300 MHz, Bruker Avance III 500 MHz, and Bruker Avance III 600 MHz and indicated accordingly. The UV–vis electronic absorption spectra were obtained using a Cary 5000 spectrophotometer with a 3500 µL quartz cuvette and a 10 mm optical path length, the fluorescence emission spectra were obtained using an Agilent Cary Eclipse spectrofluorimeter with a 3500 µL quartz cuvette with four polished faces and a 10 mm optical path length, the IR and Far‐IR vibrational spectra were obtained using the Agilent Cary 660 FTIR and Agilent Cary 630 FTIR spectrometers, the Raman vibrational spectra were obtained using Xplora equipment, with a 785 nm laser, 50x objective, 100% filter and acquisition time of 5 s. Complex mass spectra were obtained on the Orbitrap Thermo Q‐Exactive, in positive polarity, in which a 50 μL solution of 1 mg mL^–^^1^ of each complex was diluted to 1 mL with H_2_O:CH_3_CN 1:1 v/v. Interaction with His was performed in LCMS using a C‐18 column eluted with a gradient of water and methanol and Orbitrap Thermo Q‐Exactive. The thermogravimetric analysis was conducted using the TGA‐DTA 6200 device with a heating ramp of 20 °C min^–^^1^, in an inert atmosphere, from 25 to 940 °C.

##### Synthesis: Di(μ‐Chloro)bis(phenylpyridinato)diplatinum (Pt_2_(phpy)_2_µ‐Cl_2_, Precursor)

The synthesis followed procedures in the literature with adaptations.^[^ ^29^ ^,^ ^43^ ^]^ In brief, 1500 mg (3.61 mmol) of K_2_PtCl_4_ were added to 30 mL of distilled water, forming a reddish solution that was left stirring in a reaction flask under an argon atmosphere, then 515 μL (3.61 mmol) of 2‐phenylpyridine in 30 mL of ethoxyethanol were added, forming a clear solution that was quantitatively transferred to the reaction flask. The flask was kept under stirring under an argon atmosphere at 70 °C for around 48 h, when a greenish precipitate was formed. The solid was filtered, washed with water and ethanol, and left to dry under vacuum. 1065 mg of the brownish–green complex, totaling 77.2% yield. Insoluble in most solvents, it undergoes solvolysis in dimethyl sulfoxide (DMSO). IR cm^−1^: 3003 (*υ *= C—H aromatic ring); 1607, 1582, 1484, 1424 (*υ* C=C aromatic ring); 743 (*δ *= C—H ortho‐disubstituted aromatic ring); 386 (*δ* Pt‐N^C); 328 (*υ* C‐Pt‐Cl symmetric), 235 (*υ* N‐Pt‐Cl symmetric). RAMAN cm^−1^: 241 (*υ* N‐Pt‐Cl asymmetric); 319 (*υ* C‐Pt‐Cl symmetric); 382 (*δ* Pt‐N^C). Anal. Calcd (%) for C_22_H_16_Cl_2_N_2_Pt_2_: C 34.34; H 2.10; N 3.64. Found (%): C 35.41; H 1.76; N 3.75).

##### Synthesis: Chlorodimethy lsulfoxidephenylpyrid ineplatinum(II)(PtCl(DMSO)(phpy), (1)

The synthesis followed procedures from Godbert and coworkers.^[^ ^43^ ^]^ In brief, a 28 mL brownish solution of the precursor, 1.065 g, in DMSO was prepared under stirring when 120 mL of distilled water was added to the flask, resulting in precipitation. The system was left stirring for 20 h at 25 °C. The solid was separated by filtration, washed with distilled water, and dried under vacuum. Green solid (896.6 mg), yield 69.8%. ^1^H NMR (250 MHz; CDCl_3_) *δ* ppm: 9.62 (d, 1H, phpy, ^3^J_H–H_ = 6.13 Hz,^3^J_Pt–H_ = 18.18 Hz); 8.33 (d, 1H, phpy, ^3^J_H–H_ = 6.81 Hz, ^3^J_Pt–H_ = 18.08 Hz); 7.88 (t, 1H, phpy, ^3^J_H–H_ = 7.77 Hz); 7.73 (d, 1H, phpy, ^3^J_H–H_ = 7.95 Hz); 7.51 (d, 1H, phpy, ^3^J_H–H_ = 6.71 Hz); 7.26–7.20 (m, 3H, phpy); 3.65 (s, 6H, C**H**_**3**_ DMSO, ^3^J_Pt–H_ = 12 Hz). ^13^C NMR (125 MHz; CDCl_3_) *δ* ppm: 166.17 and 150.21 (pyridine ring of phpy); 144.51, 140.50, and 140.22 (pyridine and phenyl rings of phpy); 134.22, 130.93, 125.41, and 123.93 (phenyl ring of phpy); 122.03 and 118.73 (pyridine ring of phpy); 47.35 (**C**H_3_ DMSO). IR cm^−1^: 2993 (*υ* = C—H aromatic ring); 1608, 1589, 1559, 1490 (*υ* C=C aromatic ring); 1023 (*υ* S=O); 746 (*δ* = C—H ortho‐disubstituted aromatic ring); 445 (*υ* Pt‐S); 258 (*υ* Pt—Cl). RAMAN (cm^−1^): 265 (*υ* Pt—Cl); 383 (*δ* Pt—N^C); 445 (*υ* Pt‐S) Anal. Calcd (%) for C_13_H_14_ClNOPtS: C 33,73; H 3,05; N 3,03. Found (%): C 33.83; H 2.62; N 3,01. ESI^+^ MS (*m*/*z*): [M—Cl]^+^ calcd. (427.04384 m/z) found (427.04353); [M—Cl+ CH_3_CN]^+^ calcd. (468.07038 m/z) found (468.07008).

##### Synthesis: Chloro‐Phenylpyridine‐Triphenylphosphineplatinum(II) ([PtCl(phpy)(PPh_3_)], 2)

The synthesis was adapted from Samoei and coworkers.^[^ ^39^ ^]^ In brief, 222.7 mg (0.85 mmol) of triphenylphosine, PPh_3_, was added to the greenish ketone solution (36 mL) of 1 (393 mg, 0.85 mmol) under stirring. A precipitate is formed immediately and is left under stirring for 24 h at 25 °C. Then, the precipitate was filtered, washed with ice‐cold acetone, and subsequently dried under vacuum. Yellowish–green solid, 403.2 mg, yield 73.4%.^1^H NMR (250 MHz; CDCl_3_) *δ* ppm: 9.89 (d, 1H, phpy, ^3^J_H–H_ = 9.75 Hz, ^3^J_Pt–H_ = 16.86 Hz); 7.91–7.71 (m, 8H, phpy and PPh_3_); 7.54–7.26 (m, 11H, phpy and Ph_3_P); 6.96 (t, 1H, phpy, ^3^J_H–H_ = 7.46 Hz); 6.67 (d, 1H, phpy, ^3^J_H–H_ = 7.84, ^3^J_Pt–H_ = 27.48 Hz); 6.52 (t, 1H, phpy, ^3^J_H–H_ = 7.78 Hz). ^13^C NMR (125 Hz; CDCl_3_) *δ* ppm: 165.77 and 149.45 (pyridine ring of phpy); 145.58, 141.91, 139.45 and 138.36 (pyridine and phenyl rings of phpy); 135.58, 130.75 and 130.26 (PPh_3_ aromatic ring); 129.64 (phenyl ring of phpy); 127.87 (PPh_3_ aromatic ring); 123.53, 123.32, 122.00 and 118.23 (pyridine and phenyl rings of phpy). ^31^P NMR (101.25 MHz; CDCl_3_) *δ* ppm: 23.57 (^1^J_Pt–P_ = 2175.3 Hz). IR cm^−1^: 3046 (*υ*  = C—H phpy and Ph_3_P aromatic ring); 1605, 1582, 1559, 1480 (*υ* C=C phpy and PPh_3_ aromatic ring); 744 (*δ* = C—H ortho‐disubstituted phpy aromatic ring); 690 (*δ* = C—H PPh_3_ monosubstituted aromatic ring); 540 (*υ* Pt—P); 282 (*υ* Pt—Cl). RAMAN cm^−1^: 283 (*υ* Pt—Cl); 378 (*δ* Pt—N^C); 543 (*υ* Pt—P); 690 (*δ* = C—H PPh_3_ monosubstituted aromatic ring). Anal. Calcd (%) C_29_H_23_ClNPPt: C 53.83; H 3.58; N 2.16. Found (%): C 53.93; H 3.14; N 2.17. ESI^+^ MS (*m*/*z*): [M—Cl]^+^ calcd. (611.12104) found (611.12064); [M–Cl + CH_3_CN]^+^ calcd. (652.14759) found (652.14686).

##### Synthesis: Chloro‐Phenylpyridine‐1,3,5‐Triaza‐7‐Phosphaadamantaneplatinum(II) ([PtCl(phpy)(PTA)], 3)

The synthesis was adapted from Solomatina and coworkers.^[^ ^41^ ^]^ In brief, 33.89 mg (0.21  mmol) of 1,3,5‐Triaza‐7‐phosphaadamantane, PTA, was added to a greenish solution of 1, 100 mg (0.21  mmol) in CH_2_Cl_2_ (30 mL). The solution was kept under stirring at 25 °C for 6 h, when 10 mL of hexane was added, and the mixture was left to evaporate slowly, forming a crystalline greenish–yellow precipitate. The precipitate is washed with hexane and dried under vacuum. Greenish–yellow solid, 66.9 mg, yield of 57.0%. ^1^H NMR (250 MHz; CDCl_3_) *δ* ppm: 9.59 (d, 1H, phpy, ^3^J_H–H_ = 9.95 Hz, ^3^J_Pt–H_ = 14.97 Hz); 7.86 (t, 1H, phpy, ^3^J_H–H_ = 7.51 Hz); 7.76 (d, 1H, phpy, ^3^J_H–H_ = 8.07 Hz); 7.59 (d, 1H, phpy, ^3^J_H–H_ = 4.66 Hz); 7.43 (m, 1H, phpy; 7.32 (t, 1H, phpy, ^3^J_H–H_ = 6, 12 Hz); 7.20–7.12 (m, 2H, phpy); 4.59–4.54 (m, 12 H, C**H**_**2**_ PTA). ^13^C NMR (125 MHz; CDCl_3_) *δ* ppm: 164.99 and 148.02 (pyridine ring of phpy); 145.69, 140.03, 139.69, and 135.96 (perydine and phenyl rings of phpy); 130.85, 124.60, and 124.23 (phenyl ring of phpy); 122.29 and 118.43 (pyridine ring of phpy); 73.44 (N—**C**—N of PTA) and 51.29 (P—**C**—N of PTA). ^31^P NMR (101.25 MHz; CDCl_3_) *δ* ppm: −67.50 (^1^J_Pt–P_ = 1933.86). IR cm^−1^: 3041 (*υ* = C—H aromatic ring); 1603, 1583, 1,483, 1437 (*υ* C=C aromatic ring); 1419 (*δ* P—CH_2_ PTA ring); 737 (*δ* = C—H ortho‐disubstituted aromatic ring); 582 (*υ* Pt—P); 390 (*δ* Pt—N^C); 274 (*υ* Pt—Cl). RAMAN cm^−1^: 279 (*υ* Pt—Cl); 392 (*δ* Pt—N^C); 586 (*υ* Pt‐P). Anal. Calcd (%) for C_17_H_20_ClN_4_PPt: C 37.68; H 3.72; N 10.34. Found (%): C 37.75; H 3.29; N 10.19. ESI^+^ MS (*m*/*z*): [M ‐ Cl]^+^ calcd. (506.10678) found (506.10618); [M–Cl + CH_3_CN]^+^ calcd. (547.13333) found (547.13296).

##### Synthesis: Chloro‐Phenylpyridine‐p‐Benzoicaciddiphenylphosphineplatinum(II) ([PtCl(phpy)(PPh_2_(Ph*p*‐COOH))], 4)

The synthesis was adapted from Solomatina and coworkers.^[^ ^41^ ^]^ In brief, 99.45 mg (0.33 mmol) of 4‐(diphenylphosphino)benzoic acid, PPh_2_(Ph*p*‐COOH), was added to a greenish solution of 1 (150 mg, 0.33 mmol) in CH_2_Cl_2_ (20 mL). The greenish solution was left stirring at 25 °C for 5 h, when 10 mL of hexane was added, and the solvent evaporated slowly. The yellow precipitate was washed with hexane and dried under vacuum. Yellow solid, 159.7 mg, yield 71.3%. ^1^H NMR (250 MHz; CDCl_3_) *δ* ppm: 9.87 ppm (d, 1H, phpy, ^3^J_H–H_ = 8.78 Hz, ^3^J_Pt–H_ = 14.05 Hz); 8.02 (d, 2H, PPh_2_(Ph*p*‐COOH), ^3^JH‐H = 8.85 Hz); 7.93–7.71 (m, 8H, phpy and PPh_2_(Ph*p*‐COOH); 7.55–7.28 (m, 8H, phpy and PPh_2_(Ph*p*‐COOH); 6.97 (t, 1H, phpy, ^3^J_H–H_ = 8.22 Hz; 6.65 (d, 1H, phpy, ^3^J_H–H_ = 8.24 Hz, ^3^J_Pt–H_ = 19.89 Hz); 6.52 (t, 1H, phpy, ^3^J_H–H_ = 7.61 Hz). ^13^C NMR (125 MHz; DMSO‐d^6^) *δ* ppm: 167.23 (**C**OOH of PPh_2_(Ph*p*‐COOH ring); 165,46 and 148.74 (pyridine ring of phpy); 145.97, 141.82, 141.41, and 137.74 (phenyl and pyridine rings of phpy); 135,71, 134.94, 132.97, and 131.86 (PPh_2_(Ph*p*‐COOH ring); 130.01, 129.82, and 129.33 (phenyl and PPh_2_(Ph*p*‐COOH rings) 128.94 and 128,85 (Ph*p*‐COOH ring); 124.83; 124.06; 123.33, and 119.82 (phenyl and pyridine rings). ^31^P NMR (101.25 MHz; CDCl_3_) *δ* ppm: 24.19 (^1^J_Pt–P_ = 2182.99 Hz). IR cm^−1^: 3419 (*υ* O‐H); 3047 (*υ* =C–H phpy and PPh_2_(Ph*p*‐COOH) aromatic ring) 1689 (*υ* C=O); 1603, 1564, 1,483, 1435 (*υ* C=C phpy and PPh_2_(Ph*p*‐COOH) aromatic ring); 1265 (*υ* C—O); 854 (*δ *= C–H PPh_2_(Ph*p*‐COOH) para‐disubstituted aromatic ring); 752 (=C—H ortho‐disubstituted phpy aromatic ring); 694 (*δ *= C—H PPh_2_(Ph*p*‐COOH) monosubstituted aromatic ring), 553 (*υ* Pt—P); 374 (*δ* Pt—N^C); 282 (*υ* Pt—Cl). RAMAN cm^−1^: 283 (*υ* Pt—Cl); 378 (*δ* Pt—N^C); 556 (*υ* Pt—P). Anal. Calcd. (%) for C_30_H_23_ClNO_2_PPt + H_2_O: C 50.82; H 3.55; N 1.98. Found (%): C 51.36; H 3.24; N 1.92. ESI^+^ MS (*m*/*z*): [M–Cl]^+^ calcd. (655.11087) found (655.11115); [M–Cl + CH_3_CN]^+^ calcd. (696.13742) found (696.13777); [M–Cl + DMSO]^+^ calcd. (733.12480) found (733.12488).

##### Synthesis: Chloro‐Tris‐2‐Carboxyethylphosphine Platinum(II) ([PtCl(phpy)(TCEP))], 5)

The synthesis was adapted from Echeverri and coworkes.^[^ ^76^ ^]^ An acetone solution (20 mL) containing 90 mg (0.19 mmol) of 1 was prepared under stirring and an argon atmosphere. An aqueous solution (8 mL) of TCEP‐HCl, 55.8 mg (0.19 mmol), forming a yellowish solution, which was kept under stirring for 24 h. The acetone was removed in the rotatory evaporator, and distilled water was added, forming a yellow suspension. The solid was filtered, washed with water, and dried under vacuum. Yellow solid 59.9 mg, yield 48.5%. ^1^H NMR (250 MHz; DMSO‐d^6^) *δ* ppm: 12.36 (s, 2H, P–CH_2_–CH_2_–COO**H**); 9.03 (d, 1H, phpy, ^3^J_H–H_ = 5.84 Hz); 8.14 (m, 2H, phpy); 7.77 (d, 1H, phpy, ^3^J_H–H_ = 7.54 Hz); 7.62 (m, 1H, phpy); 7.30 (d, 1H, phpy, ^3^J_HH_ = 7.19 Hz, ^3^JPt–H = 24.36 Hz); 7.14–7.03 (m, 2H, phpy); 2.69‐2.08 (m, 12 H, P—C**H**_2_—C**H**_2_—COOH). ^13^C NMR (125 MHz; DMSO‐d^6^) *δ* ppm: 174.53, 173.62 and 173.50 (P—CH_2_—CH_2_ **—C**OOH of TCEP); 164.04 and 145.92 (pyridine ring of phpy); 145.46, 141.72, 137.02, and 135.55 (pyridine and phenyl rings of phpy); 131.08, 125.12, and 124.27 (phenyl ring of phpy) 123.06 and 119.69 (pyridine ring of phpy); 32.63; 30.21; 19.62; 19.32; 19.02, and 18.71 (P—**C**H_2_—**C**H_2_—COOH of TCEP). ^31^P NMR (101.25 MHz; DMSO‐d^6^) *δ* ppm: 12.13 (^1^J_Pt–P_ = 2014.97 Hz). IR cm^−1^: 3437 (*υ* O—H); 1739 (*υ* C=O); 1609, 1569, 1555, 1490 (*υ* C=C aromatic ring); 1426 (*δ* P—CH_2_); 1243 (*υ* C—O); 749 (*δ *= C—H ortho‐disubstituted aromatic ring); 521 (*υ* Pt—P); 386 (*δ* Pt—N^C); 285 (*υ* Pt—Cl). Anal. Calcd. (%) for C_20_H_23_ClNO_6_PPt + 1/3 C_3_H_6_O: C 38.55; H 3.85; N 2.14. Found (%): C 39.26; H 3.25; N 2.27. ESI^+^ MS (*m*/*z*) [M—Cl]^+^ calcd. (599.0905) found (599.0906).

##### Ligand Exchange Reactions with DMSO

NMR analyses were performed to study the ligand exchange reactions of the five complexes with DMSO. ^1^H and ^31^P NMR spectra of complexes 2–4 were initially recorded in CDCl_3_, followed by the addition of 10 equivalents of DMSO, with new spectra recorded after 24 and 48 h. Complex 1 was analyzed only by ^1^H NMR. Due to solubility reasons, 5 was dissolved in DMSO‐d^6^, and was analyzed using ^1^H and ^31^P NMR at the time of preparation and after 24 and 48 h, without the addition of nondeuterated DMSO.

##### Ligand Exchange Reactions with *N*‐Acetylcysteine and Histidine

To investigate the chloride to *N*‐acetylcysteine (Nac) exchange, UV–vis titrations were performed using a double beam spectrophotometer to assess the reactivity of complexes 1–5 toward thiol‐containing ligands. The reference cuvette contained 2 mL of DMSO, while the sample cuvette held a 3.24 × 10^−5 ^mol L^−1^ DMSO solution of the complex. After baseline correction and acquisition of the initial spectra, successive aliquots of a 20 × 10^−3 ^mol L^−1^ Nac stock solution in DMSO were added to both cuvettes. After each addition, solutions were homogenized prior to spectral acquisition. The titration began at a [Nac]/[Complex] ratio of 20 and progressed in 20‐equivalent increments to a final ratio of 220. All spectra were corrected for dilution. A systematic decrease in absorbance at ≈375 nm (MLCT band of the parent complex) and a concomitant increase at ≈425 nm (MLCT band of Nac‐bound species) were monitored throughout the titration. The analysis was therefore carried out in 100% DMSO.

The chloride to Histidine (His) exchange analysis was performed for complexes 1–5. Initially, 500 μL stock solutions of complexes were prepared in DMSO‐d^6^, yielding concentrations of 3.69 × 10^−3 ^mol L^−1^. The ^1^H and ^31^P NMR spectra of stock solutions were recorded. Subsequently, a 100 μL His stock solution in D_2_O at a concentration of 0.276 mol L^−1^ was added to the NMR tube and agitated. The final molar ratio [His]/[complex] was 15. After 24 h, additional ^1^H and ^31^P NMR measurements were performed.

##### Computational Details

To further explore the thermodynamics of the ligand exchange reactions with DMSO and Nac, theoretical calculations were performed. The structures of the complexes 1–5, as well as the DMSO and Nac and the reaction products [Pt(phpy)(DMSO)_2_]^+^, [Pt(phpy)(PPh_3_)DMSO]^+^, [Pt(phpy)(PTA)DMSO]^+^, [Pt(phpy){PPh_2_(Ph*p*‐COOH)}DMSO]^+^, [Pt(phpy)(TCEP)DMSO]^+^, [Pt(phpy)(DMSO)Nac], [Pt(phpy)(PPh_3_)Nac], [Pt(phpy)(PTA)Nac], [Pt(phpy){PPh_2_(p‐COOH)}Nac], and [Pt(phpy)(TCEP)Nac] and Cl^−^, were optimized using DFT calculations via the ORCA v. 6.0 package.^[^ ^77^ ^,^ ^78^ ^]^ The PBE0 functional was employed,^[^ ^79^ ^]^ with the SARC‐ZORA‐TZVP basis set for Pt(II),^[^ ^80^ ^]^ ZORA‐def2‐TZVP basis functions for other atoms,^[^ ^81^ ^]^ SARC/J auxiliary basis functions, the RIJCOSX approximation,^[^ ^82^ ^]^ and CPCM for implicit DMSO solvation.^[^ ^83^ ^,^ ^84^ ^]^ A convergence criterion of 1.0 × 10^−8 ^a.u. was used. The ligand exchange reaction between complexes 1–5 and His was also computed, for this, [Pt(phpy)(DMSO)His]^+^, [PtCl(phpy)His], [Pt(phpy)(PPh_3_)His]^+^ [Pt(phpy)(PTA)His]^+^, [Pt(phpy){PPh_2_(Ph*p*‐COOH)}His]^+^ and [Pt(phpy)(TCEP)His]^+^ structure were optimized in the same level of theory of the previous geometry optimizations.

Frequency calculations were performed at the same theoretical level as the geometry optimizations to confirm the presence of minima (by the absence of imaginary frequencies) and to compute thermodynamic data at 298.15 K. The ΔG of each reaction was then calculated by the difference between the sum of the energies of the products and reactants of each reaction. Additionally, frequency calculations were employed to interpret the experimental Raman and Infrared results.

BDE was the energy required for homolytic breakage of a specific bond. The Pt–Cl BDE was calculated for 1–5, following the protocol of Wright,^[^ ^85^ ^]^ via calculation of the enthalpy change in the homolytic breakage of a bond. The radical structures, [Pt(phpy)(DMSO)]˙, [Pt(phpy)(PTA)]˙, [Pt(phpy)(COOH)]˙, [Pt(phpy)(PPh_3_)]˙, [Pt(phpy)(TCEP)]˙, and Cl˙, were optimized and frequency calculations were performed using the same level of theory as used for the 1–5. The ΔH of the bond dissociation reactions was calculated by the difference between the sum of the enthalpies of the products and the initial complex of each reaction. TD‐DFT calculations, at the same level of theory, were carried out for complex 5, where the number of excited states to be calculated was 30, for the characterization of its UV–vis spectra.

##### BSA Interaction by Intrinsic Fluorescence Quenching and Stern–Volmer Treatment

DMSO stock solutions (1 mM) of complexes 1, 3, 4, and 5, and 690 µM for complex 2 were prepared. A 100 mM Tris‐HCl buffer (pH 7.4) solution of BSA was also prepared at a final concentration of 3.78 × 10^−4 ^mol L^−1^. The BSA concentration was determined spectrophotometrically using its molar absorptivity (*ε *= 43,824 M^−1 ^cm^−1^ at 280 nm). For fluorescence titration, a Tris‐HCl buffer solution of BSA was placed in a quartz cuvette, yielding a final concentration of 378 µM. Platinum complexes were titrated into the BSA solution in successive additions, covering a [Complex]/[BSA] molar ratio range from 0.05 to 0.9. After each addition, the mixture was equilibrated for 1 min prior to fluorescence measurement. Spectra were recorded using an excitation wavelength of 280 nm and emission monitored from 300 to 500 nm. The platinum complexes showed no intrinsic fluorescence in this range upon 280 nm excitation, ensuring no interference in the analysis. The emission intensity at ≈350 nm (BSA emission maximum) was analyzed using the Stern–Volmer Equation (1), where *K*_SV_ is the Stern–Volmer constant, *F*_0_ and *F* are the relative fluorescence intensity in the absence and presence of the quencher, and [*Q*] is the concentration of the quencher.

(1) $$F_{0}/F=1+K_{\mathrm{SV}}\left[Q\right]$$

The Scatchard Equation (2) is used to predict the number of interaction sites (*n*) and the equilibrium constant (*K*_b_) between BSA and the quencher.^[^ ^1^ ^,^ ^57^ ^,^ ^70^ ^]^

(2) $$\mathrm{Log}\left(\frac{F_{0}-F}{F}\right)=\mathrm{Log}Kb+n\mathrm{Log}\left[Q\right]$$

##### CT–DNA Interaction via Ethidium Bromide Competition through Fluorescence Emission Analysis

A solution of calf thymus DNA (CT‐DNA) was prepared in 100 mM Tris‐HCl buffer (pH 7.4) containing 500 mM NaCl. The DNA concentration was determined to be 283.05 µM using its molar absorptivity (*ε* = 6600 M^−1^cm^−1^) at 260 nm. An A_260_/A_280_ absorbance ratio greater than 1.8 confirmed the absence of protein contamination.^[^ ^86^ ^]^ Stock solutions of 1, 3, 4, and 5 (1.0 mM) and 2 (0.7 mM) were prepared in DMSO. To assess whether the complexes interact with DNA through an intercalative binding mode, a competitive assay with ethidium bromide (EB) was conducted using fluorescence emission spectroscopy, aiming to determine which complex is capable of displacing EB from the CT‐DNA–EB adduct. The experiments were conducted in a quartz cuvette with a 1 cm optical path length, containing 30 µM CT‐DNA and 23 µM EB in 100 mM Tris‐HCl buffer (pH 7.4) containing 500 mM NaCl, while the concentration of each complex was varied from 0 to 100 µM. The solutions were excited at 540 nm, and the emission spectra were recorded in the range of 560–800 nm. The Stern–Volmer quenching constants (K_SV_) were calculated using Equation (1). It is known that K_SV_ = kq*τ_0_*, where kq represents the quenching rate constant, and *τ_0_* corresponds to the lifetime of the CT‐DNA–EB adduct (*τ_0_* = 23.0 × 10^−9^ s). An equation similar to Equation (2) was employed; however, in this case, *K*_b_ *=* *K*_f_ which denotes the fluorescence binding constant, while *n* represents the number of binding sites per nucleotide.^[^ ^67^ ^,^ ^68^ ^,^ ^70^ ^]^

##### Octanol/Water Partition Coefficient Determination by Shake Flask Methodology

The logarithmic partition coefficients (log P) of the platinum complexes between 1‐octanol and water were determined using a modified shake‐flask method based on OECD guidelines, with spectrophotometric quantification of the platinum species in the organic phase.^[^ ^1^ ^,^ ^56^ ^,^ ^87^, ^88^, ^89^, ^90^ ^]^ Prior to the experiments, water and 1‐octanol were mutually saturated by stirring together for 48 h. Calibration curves for each complex were established by measuring the UV–vis absorbance in the range of 373–379 nm, using 1‐octanol solutions with concentrations between 35 and 180 µM. To enhance solubility, DMSO was added: 5% (v/v) for most complexes and 15% (v/v) for Complexes 2 and 3. It is important to note that these represented the lowest possible DMSO concentrations in the 1‐octanol solutions of each complex required to obtain concentrated and precipitate‐free solutions, from which the calibration curves were subsequently constructed by dilution. Partitioning experiments were performed in triplicate. Each sample consisted of 7.5 mL of a solution stock close to 100 µM of the respective complex in water‐saturated 1‐octanol (with the corresponding DMSO content), mixed with 7.5 mL of 1‐octanol‐saturated water, resulting in a total volume of 15 mL. The mixtures were placed in conical centrifuge tubes, shaken mechanically for 4 h at 25 °C, and subsequently centrifuged at 4000 rpm for 15 min to separate the phases. The 1‐octanol phase was collected and analyzed by UV–vis spectroscopy. It should be noted that DMSO exhibits its own log P;^[^ ^91^ ^]^ thus, for each complex, a suitable amount of DMSO was added to the 1‐octanol phase prior to UV–vis measurements to ensure that its concentration matched that of the stock solution and the solution used to construct the corresponding calibration curve. Complex concentrations were determined using the calibration curves. Aqueous concentrations were calculated indirectly by subtracting the amount present in the 1‐octanol phase, after the mechanical shaking analysis, from the total concentration in the initial stock solution, due to the insufficient solubility of the complexes in water to allow direct calibration. This is shown in Equation (3). The log *p* value was subsequently determined using Equation (4).

(3) [Complex]water=[Complex]stock−[Complex]octanol

(4) $$\log P=\log\frac{\left[\text{Complex}\right]\text{octanol}}{\left[\text{Complex}\right]\text{water}}$$

##### Interaction of the Complexes with Nac, L‐Cysteine (Cys), and His by Fluorescence

Solutions of 1–5 (70 µM) were prepared in 100 mM Tris‐HCl buffer (pH 7.4), with DMSO concentrations between 7% and 10% (v/v) to adjust solubility. Their fluorescence emission spectra were obtained using the excitation wavelengths determined previously by measuring excitation and emission spectra, and are indicated in the results. Interaction with Nac, Cys, and His was performed in the kinetic mode. The kinetics were performed in the molar ratio [amino acid]: [complex] 5:1. The spectra were acquired every 3 min from 0 to 60 min. Only 3 interacted with His turning on the emission. For this reason, 3 was evaluated by titration. The titration was performed in the same previous conditions by adding a fixed volume of a His stock solution at 10 mM in the same buffer, to create a range of molar ratios from 5 to 55 equivalents of amino acid for mol of complex. Solvents and pH were varied to test the solvent and pH effects. These experiments were conducted in four different solvent media: 100 mM Tris‐HCl, pH 7.4 with 13.3% DMSO (v/v); DMSO solution with 16.6% water (v/v); DMSO (100%); 100 mM acetate buffer, pH 5.2. The Complex 3 was dissolved in 110 µM, and His was added in 15 equivalent molar excess.

##### Mass Spectrometric Analysis of the Interaction between Complex 3 and Histidine

A sample of 1 mg of Complex 3 was dissolved in 900 μL of DMSO and mixed with an aqueous solution containing 4.29 mg of His in 100 μL, yielding a 1:15 molar ratio between Complex 3 and His. The resulting mixture was filtered through a 0.22 μm syringe filter. The filtrate was further diluted in water and analyzed by LCMS using a C‐18 column, eluted with a water and methanol gradient in positive ion mode.

##### Antiviral Activity: Vero E6 Cell Culture

Vero E6 cells (kidney tissue derived from an African green monkey, American Type Culture Collection ATCC CRL‐1586) were cultured in modified Dulbecco's Modified Eagle Medium (DMEM; Sigma–Aldrich) supplemented with 100 U mL^–1^ penicillin (Gibco Life Technologies), 100 μg mL^–1^ streptomycin (Gibco Life Technologies), 1% (v/v) nonessential amino acids (Gibco Life Technologies), and 10% (v/v) fetal bovine serum (FBS; Hyclone) at 37 °C in a humidified 5% CO_2_ incubator.

##### Antiviral Activity: Vero E6 Cell Viability Assay

Cell viability was measured by the MTT ((3‐(4,5‐dimethylthiazol‐2‐yl)−2,5‐diphenyltetrazolium bromide) assay. Vero E6 cells were seeded in 96‐well plates at a density of 2 × 10^4^ cells per well in 48‐well plates for MAYV‐*nanoluc* assays and 5 × 10^3^ cells per well for ZIKV assays. Cells were incubated overnight at 37 °C in a 5% CO_2_ humidified incubator. To the cell culture medium, solutions of the complexes at concentrations of 50, 10, and 2 μM were added, and these solutions were incubated with these cells at 37 °C for 24 or 72 h, respectively, corresponding to the infection assay time for each virus, MAYV or ZIKV. DMSO 0.1% was used as an untreated control. Then, the medium was removed, and 1 mg mL^–^^1^ of MTT solution was added to each well and incubated for 30 min at 37 °C in a 5% CO_2_ humidified incubator. Then, the medium was replaced with 100 μL of DMSO to solubilize the formazan crystals. Subsequently, absorbance was measured by optical density (OD) of each well at 560 nm using the GloMax microplate reader (Promega). Cell viability was calculated according to the Equation (T/C) × 100%, where T and C represent the mean OD of the treated group and the vehicle control group, respectively. Data were analyzed for normal distribution to demonstrate the applicability of parametric or nonparametric tests. Then, a two‐way ANOVA test was employed to compare the treatment of each complex with the DMSO control, with a *p* < 0.05.

##### Antiviral Activity: Antiviral Activity Assays—ZIKV

A wild‐type ZIKV sample isolated from a clinical sample from a patient in Brazil (ZIKV_PE243_)^[^ ^92^ ^]^ was amplified using Vero E6 cells in a 75 cm^2^ flask for 3 days. Then, the viral supernatant was collected and stored at –80 °C. To determine viral titers, 5 × 10^3^ Vero E6 cells were seeded in each well of 96‐well plates for 24 h before infection. Then, cells were infected with a tenfold serial dilution of ZIKV_PE243_ and incubated for 72 h in a humidified 5% CO_2_ incubator at 37 °C. Then, the cells were fixed with 4% (v/v) formaldehyde, washed with PBS [1x], and incubated with blocking buffer (BB) containing 0.1% (v/v) Triton X‐100, 0.2% (w/v) BSA, and PBS for 30 min, for immunofluorescence assay as previously described.^[^ ^93^ ^]^

To evaluate the antiviral activity of each complex, Vero E6 cells were seeded at a density of 5 × 10^3^ cells per well in 96‐well plates for 24 h and then infected with ZIKV_PE243_ at a multiplicity of infection of 0.01 in the presence of each complex at the established noncytotoxic concentration for 72 h. DMSO 0.1% and Obatoclax (OLX)^[^ ^73^ ^,^ ^74^ ^]^ at 1 µM were used as negative and positive controls, respectively. Cells were fixed with 4% (v/v) formaldehyde, washed with PBS [x1], and stained with BB for the immunofluorescence assay. Focus‐forming units were measured at EVOs cell fluorescence microscopy (Thermo Fisher Scientific) and calculated according to the Equation (T/C) × 100%, where T and C represent the mean OD of the treated group and the vehicle control group, respectively. Data were analyzed for normal distribution to demonstrate the applicability of parametric or nonparametric tests. Then, a two‐way ANOVA test was employed to compare the treatment of each complex with the DMSO control, with a *p* < 0.05.

##### Antiviral Activity: Antiviral Activity Assays—MAYV

MAYV‐*nanoluc* was produced and rescued as previously described.^[^ ^71^ ^]^ To determine viral titers, 8 × 10^4^ Vero E6 cells/well were seeded in 24‐well plate, and 24 h later, cells were infected with a tenfold serial dilution of MAYV infectious clone. Cells were incubated with virus for 1 h at 37 °C and 5% CO_2_, followed by the inoculum removal, washed with PBS to remove the unbound virus, and the addition of fresh medium supplemented with 1% dilution of stock of penicillin and streptomycin, 2% FBS, and 1% carboxymethyl cellulose (CMC). Infected cells were incubated for 2 days in a humidified 5% CO_2_ incubator at 37 °C, followed by fixation with 4% formaldehyde and staining with 0.5% violet crystal. The viral foci were visualized to determine plaque morphology.

To evaluate the antiviral activity of the complexes, Vero E6 cells were seeded at a density of 2 × 10^4^ cells per well in 48‐well plates. After 24 h, cells were infected with MAYV‐*nanoluc* in the presence of each complex at its highest noncytotoxic concentration for 24 h. DMSO 0.1% and EIDD2749 at 10 µM (4^′^‐Fluorouridine, molnupiravir prodrug) were used as negative and positive controls, respectively.^[^ ^71^ ^,^ ^72^ ^]^ Then, samples were lysed with Renilla‐luciferase lysis buffer (Promega), and the levels of viral replication were quantified by measuring the nanoluciferase activity using the Renilla‐luciferase assay (Promega) with a GloMax microplate reader (Promega). The antiviral activity was calculated according to the Equation (T/C) × 100%, where T and C represent the mean of the treated and the vehicle control groups, respectively. Data were analyzed for normal distribution to demonstrate the applicability of parametric or nonparametric tests. Then, a two‐way ANOVA test was employed to compare the treatment of each complex with the DMSO control, with a *p* < 0.05.

##### Antiparasitic Activity: Parasites culture

*Leishmania* (*Leishmania*) *amazonensis* (IFLA/BR/67/PH8 ‐ The parasite strain was kindly donated by Dr Norma Andrews, University of Maryland at College Park, United States) promastigotes were cultivated at 25 °C in 25 cm^2^ tissue culture flasks containing M199 medium (Gibco‐BRL, Invitrogen) pH 7.4 supplemented with 10% heat‐inactivated fetal bovine serum, FBS (Invitrogen), 5% penicillin/streptomycin, 0.1% hemin (25 mg mL^–^^1^ in 50% triethanolamine), 10 mM adenine (pH 7.5), and 5 mM L‐glutamine, as previously described.^[^ ^94^ ^]^ Cultures were split every 5 days, after promastigotes reached the late logarithmic growth phase.

##### Antiparasitic Activity: Extraction of Primary Host Cell

BMDM were obtained after differentiation of precursor cells extracted from femurs and tibias of female Balb/C mice. The medullary cavities of the bones were washed with a 10 mL syringe and a 21G needle with Roswell Park Memorial Institute (RPMI 1640) medium (Gibco‐Invitrogen) supplemented with 20% FBS and 30% L929 fibroblast culture supernatant in 75 mm Petri dishes. The L929 fibroblast lineage was a gift from Dr Silvia Uliana, University of São Paulo, Brazil. Recovered cells were kept at 37 °C with 5% CO_2_ atmosphere. After 5 days, 5 mL of medium was added to each Petri dish, and after 7 days, differentiated BMDM were collected with fresh RPMI medium after scraping the plate with a sterile cell scraper (Biofil). The Ethics Committee on Animal Use of the University of Campinas (CEUA‐UNICAMP) (protocol 6022‐1/2022) approved all procedures using mice.

##### Antiparasitic Activity: Promastigotes and BMDM Assays

Cell viability was evaluated by the MTT assay using log phase *Leishmania* promastigotes (5 × 10^6^ cells/well) or BMDM (5 × 10^4 ^ cells/well) in 96‐well culture plates incubated with decreasing concentrations of the five platinum complexes (12; 6; 3; 1.5; 0.75; 0.37 μM) for promastigotes and (100; 50; 25; 12.5; 6.25; 3.13 μM) for BMDM for 24 h. The ligand PTA was also tested in parallel to exclude leishmanicidal activity, along with a group containing up to 1% (v/v) DMSO as a diluent (negative control) and Ambisome (AMB, 5 mM stock concentration; Sigma–Aldrich, USA) diluted in cell culture media as a positive control. The complex was freshly solubilized in pure sterile DMSO (Sigma–Aldrich) at 10 mM stocks for each experiment. Briefly, 30 μL of MTT (5 mg mL^–^^1^) was added to each well, and the plates were incubated for 3 h at 25 °C (for promastigotes) and 37 °C (for macrophages). Next, the reaction was stopped by the addition of 30 μL of SDS 20%. Absorbance reading was performed using a plate spectrophotometer (reference and test wavelength of 600 and 650 nm, respectively). OD values were converted to percentages compared to untreated control groups incubated with medium only (100% viability) to determine the 50% effective concentrations (IC_50_ for parasites and macrophages) from sigmoidal regression of dose–response curves (GraphPad Prism8 software, USA).

##### Antiparasitic Activity: In Vitro *L. amazonensis* Infections

To assess the viability of intracellular amastigotes, 4 × 10^5^ BMDM were cultured in each well containing a 13 mm glass coverslip in 24‐well plates. Next, cells were incubated with stationary phase promastigotes of *L*. (*L*.) *amazonensis* (5 parasites: 1 macrophage) for 24 h at 34 °C and 5% CO_2_ and then exposed or not to increasing concentrations (5 and 12 µM) of Complex 3 for 24 h. Coverslips were fixed with pure methanol and stained with an Instant Prov kit (NewProv, Brazil). The number of infected cells and amastigotes per 300 BMDM was quantified microscopically by direct counting as described.^[^ ^6^ ^]^ Infection rates (%) were determined as the proportion of BMDM containing at least one intracellular parasite in a total of 100 infected and noninfected BMDM. Inhibition data of intracellular amastigote multiplication were tallied in biological replicates.

##### Cell Viability Assay

Cell viability was evaluated by means of the MTT, according to a previously described protocol with adaptations.^[^ ^95^ ^,^ ^96^ ^]^ The MCF 7 (breast cancer), A2780 (ovarian cancer), MRC‐5 (fetal lung fibroblasts), and HaCaT nontumor cell lines (immortalized human keratinocytes) were purchased from the Banco de Células do Rio de Janeiro (BCRJ, Brazil) with codes BCJ0162, BCRJ0031, BCRJ0180, respectively, and HaCaT was donated by Prof. Carmen Silva Passos, Medical Sciences Faculty, University of Campinas, Brazil. The cells were seeded in 96‐well plates at a density of 5.10^4^ cells/well, in 100 μL of suitable culture medium (DMEM/F‐12 high glucose, supplemented with 10% FBS and 1% antibiotic). After 24 h of incubation at 37 °C with 5% CO_2_ for cell adhesion, the complexes were added in different concentrations (0.39–25 μM), diluted in culture medium with a maximum of 0.2% DMSO (v/v). A group containing the diluent only (0.2% DMSO v/v) was tested as a negative control, and a curve in the same concentration of cisplatin was used as a positive control. After 48 h of treatment, 23 μL of MTT solution (5 mg mL^–^^1^ in PBS) was added to each well, and the cells were incubated for another 4 h. Next, the medium was carefully removed, and the formazan crystals were solubilized with 100 μL of DMSO. Absorbance was measured at 570 nm using a microplate reader (Varioskansing multimode plate reader Thermo Fisher Scientific). The data were expressed as a percentage of cell viability in relation to the negative control (cells treated only with DMSO). All experiments were carried out in biological and technical triplicate. The IC_50_ values for MCF 7 were not determined because cell viability remained above 50% even at the highest concentration tested (25 μM).

## Conflict of Interest

The authors declare no conflict of interest.

## Author Contributions

**Antonio A. de Oliveira‐Neto**: synthesis and characterization of the complexes, biophysical analyses, stability, interaction with biomolecules and writing; **Gustavo Clauss**: theoretical calculations; **Jennyfer Castro**, **Nádija N. P. da Silva**, and **Fillipe V. Rocha**: cell viability assays, writing, and funding; **Marcus S. A. Garcia**: antileishmanial analyses; **Danilo C. Miguel**: antileishmanial analyses, writing, and funding; **Natasha M. Cassani**, **Bruna C. Sandim**, **Ana Laura C. Oliveira**, **Stephanie P. B. Reyes**: antiviral analysis; **Ana C. G. Jardim**: antiviral analysis, writing, and funding; **Camilla Abbehausen**: writing, review, conceptualization, data curation, acquisition of partnerships and funding.

## Supporting information

Supplementary Material

## Data Availability

All data supporting this study are provided in the Supporting Information (SI).

## References

[1] J. V. Fontes , I. A. Santos , L. B. Rosa , R. L. A. Lima , A. C. G. Jardim , D. C. Miguel , C. Abbehausen , ChemistrySelect 2022, 7, 1.

[2] L. H. Gonçalves Maciel , C. V. da Rocha Neto , Y. F. Martins , F. de Azevedo Furtado , P. C. Teixeira , M. Y. O. Dias , Y. K. B. Rodrigues , I. C. R. Piauilino , S. D. Pinto , A. C. Côrte Alencar , J. B. de L. Gimaque , M. P. G. Mourão , M. V. G. Lacerda , M. da C. Castilho , C. Bôtto‐Menezes , PLoS Negl. Trop. Dis. 2022, 16, 1. 10.1371/journal.pntd.0010727 PMC9560595 36228027

[3] L. B. Rosa , C. Galuppo , R. L. A. Lima , J. V. Fontes , F. S. Siqueira , W. A. S. Júdice , C. Abbehausen , D. C. Miguel , J. Inorg. Biochem. 2022, 229, 1. 10.1016/j.jinorgbio.2022.111726 35065320

[4] L. B. Rosa , R. L. Aires , L. S. Oliveira , J. V. Fontes , D. C. Miguel , C. Abbehausen , ChemMedChem 2021, 16, 1681. 33615725 10.1002/cmdc.202100022

[5] D. Pace , J. Infect. 2014, 69, S10. 25238669 10.1016/j.jinf.2014.07.016

[6] K. Minori , L. B. Rosa , R. Bonsignore , A. Casini , D. C. Miguel , ChemMedChem 2020, 15, 2146. 32830445 10.1002/cmdc.202000536 PMC7756297

[7] R. Dean , H. Melnyk , C. Anglin , J. Consum. Health Internet 2017, 21, 62.

[8] N. F. de Sousa , G. R. de Sousa , N. T. R. de Lima , E. B. de Assis , M. C. Aragão , É. P. de Moura , R. G. Gopalsamy , M. T. Scotti , L. Scotti , Curr. Drug Targets 2024, 25, 577. 38967077 10.2174/0113894501298864240627060247

[9] J. M. Nicacio , O. V. Gomes , R. F. do Carmo , S. L. P. Nunes , J. R. C. F. Rocha , C. D. F. de Souza , R. F. de O. Franca , R. Khouri , M. Barral‐Netto , A. da C. Armstrong , Viruses 2022, 14, 1. 10.3390/v14091988 PMC9502577 36146794

[10] R. E. Marques , J. F. Shimizu , M. L. Nogueira , N. Vasilakis , Expert Opin. Ther. Targets 2024, 28, 345. 38714500 10.1080/14728222.2024.2351504 PMC11189740

[11] J. Ferreira , D. Campos‐Ferreira , E. Figueiredo , J. L. Filho , Asian Pac. J. Trop. Biomed. 2020, 10, 95.

[12] M. Navarro , Coord. Chem. Rev. 2009, 253, 1619.

[13] M. Navarro , C. Gabbiani , L. Messori , D. Gambino , Drug Discov. Today 2010, 15, 1070. 20974285 10.1016/j.drudis.2010.10.005

[14] R. A. Sánchez‐Delgado , A. Anzellotti , L. Suárez , Met. Ions Biol. Syst. 2004, 41, 379. 15206123

[15] D. Gambino , L. Otero , Front. Chem. 2022, 9, 1. 10.3389/fchem.2021.816266 PMC8777014 35071192

[16] R. Paiva , A. Marçal Neto , I. Santos , A. Jardim , P. Corbi , F. Bergamini , Dalt. Trans. 2020, 49, 16004. 10.1039/d0dt02478c 33030464

[17] Y. C. Ong , S. Roy , P. C. Andrews , G. Gasser , Chem. Rev. 2019, 119, 73. 10.1021/acs.chemrev.8b00338 30507157

[18] N. S. Sultan , A. A. Shoukry , F. B. Rashidi , H. K. A. Elhakim , Cell Biochem. Biophys. 2024, 82, 2651. 39018004 10.1007/s12013-024-01376-9

[19] D. Rusanov , J. Zou , M. Babak , Pharmaceuticals 2022, 15, 1. 10.3390/ph15040453 PMC9031419 35455450

[20] L. Lv , T. Zheng , L. Tang , Z. Wang , W. Liu , Coord. Chem. Rev. 2025, 525, 1.

[21] V. Novohradsky , Z. Liu , M. Vojtiskova , P. J. Sadler , V. Brabec , J. Kasparkova , Metallomics 2014, 6, 682. 24448555 10.1039/c3mt00341h

[22] B. Bertrand , A. Casini , Dalt. Trans. 2014, 43, 4209. 10.1039/c3dt52524d 24225667

[23] R. Aires , I. Santos , J. Fontes , F. Bergamini , A. Jardim , C. Abbehausen , Metallomics 2022, 14, 1. 10.1093/mtomcs/mfac056 35894863

[24] C. Galuppo , A. O. Junior , L. Oliveira , P. Guarda , R. Buffon , C. Abbehausen , J. Inorg. Biochem. 2023, 240, 1. 10.1016/j.jinorgbio.2022.112117 36635196

[25] S. Q. Gomes , L. Vitoriano , E. G. R. de Arruda , A. L. T. G. Ruiz , T. Candido , J. E. de Carvalho , W. R. Lustri , C. Abbehausen , J. Inorg. Biochem. 2018, 186, 104. 29885553 10.1016/j.jinorgbio.2018.04.005

[26] R. G. Kenny , C. J. Marmion , Chem. Rev. 2019, 119, 1058. 30640441 10.1021/acs.chemrev.8b00271

[27] T. C. Johnstone , K. Suntharalingam , S. J. Lippard , Chem. Rev. 2016, 116, 3436. 26865551 10.1021/acs.chemrev.5b00597 PMC4792284

[28] M. Jamshidi , M. Babaghasabha , H. R. Shahsavari , S. M. Nabavizadeh , Dalt. Trans. 2017, 46, 15919. 10.1039/c7dt03599c 29119183

[29] N. Ghavale , A. Wadawale , S. Dey , V. K. Jain , J. Organomet. Chem. 2010, 695, 1237.

[30] Y. Ning , G. Q. Jin , M. X. Wang , S. Gao , J. L. Zhang , Curr. Opin. Chem. Biol. 2022, 66, 1. 10.1016/j.cbpa.2021.102097 34775149

[31] N. Mirzadeh , T. S. Reddy , S. K. Bhargava , Coord. Chem. Rev. 2019, 388, 343.

[32] G. H. Ribeiro , A. A. Batista , A. P. M. Guedes , T. D. de Oliveira , C. R. S. T. B. de Correia , L. Colina‐Vegas , M. A. Lima , J. A. Nóbrega , M. R. Cominetti , F. V. Rocha , A. G. Ferreira , E. E. Castellano , F. R. Teixeira , Inorg. Chem. 2020, 59, 15004. 32997499 10.1021/acs.inorgchem.0c01835

[33] B. M. Sutton , Gold Bull. 1986, 19, 15.

[34] M. Richert , R. Mikstacka , M. Walczyk , M. Cieślak , J. Kaźmierczak‐Barańska , K. Królewska‐Golińska , T. Muzioł , S. Biniak , Processes 2021, 9, 1.

[35] L. Ronga , I. Tolbatov , E. Giorgi , P. Pisarek , C. Enjalbal , A. Marrone , D. Tesauro , R. Lobinski , T. Marzo , D. Cirri , A. Pratesi , Inorg. Chem. 2023, 62, 10389. 37342994 10.1021/acs.inorgchem.3c01280 PMC10324304

[36] F. K. Keter , I. A. Guzei , M. Nell , W. E. V. Zyl , J. Darkwa , Inorg. Chem. 2014, 53, 2058. 24476103 10.1021/ic4025926 PMC3993921

[37] S. Crooke , R. Snyder , T. Butt , D. Ecker , H. Allaudeen , B. Monia , C. Mirabelli , Biochem. Pharmacol. 1986, 35, 3423. 3533080 10.1016/0006-2952(86)90608-8

[38] C. K. Mirabelli , D. T. Hill , L. F. Faucette , F. L. Mccabe , G. R. Girard , D. B. Bryan , B. M. Sutton , J. O. L. Bartus , S. T. Crooke , R. K. Johnson , J. Med. Chem. 1987, 30, 2181. 3681888 10.1021/jm00395a004

[39] H. Samouei , M. Rashidi , F. W. Heinemann , J. Iran. Chem. Soc. 2014, 11, 1207.

[40] G. L. Edwards , D. S. C. Black , G. B. Deacon , L. P. G. Wakelin , Can. J. Chem. 2005, 83, 980.

[41] A. I. Solomatina , P. S. Chelushkin , T. O. Abakumova , V. A. Zhemkov , M. Kim , I. Bezprozvanny , V. V. Gurzhiy , A. S. Melnikov , Y. A. Anufrikov , I. O. Koshevoy , S. H. Su , P. T. Chou , S. P. Tunik , Inorg. Chem. 2019, 58, 204. 30376305 10.1021/acs.inorgchem.8b02204

[42] A. I. Solomatina , P. S. Chelushkin , D. V. Krupenya , I. S. Podkorytov , T. O. Artamonova , V. V. Sizov , A. S. Melnikov , V. V. Gurzhiy , E. I. Koshel , V. I. Shcheslavskiy , S. P. Tunik , Bioconjug. Chem. 2017, 28, 426. 27977146 10.1021/acs.bioconjchem.6b00598

[43] N. Godbert , T. Pugliese , I. Aiello , A. Bellusci , A. Crispini , M. Ghedini , Eur. J. Inorg. Chem. 2007, 2007, 5105.

[44] J. Ruiz , C. Vicente , C. De Haro , A. Espinosa , Inorg. Chem. 2011, 50, 2151. 21314142 10.1021/ic101526h

[45] S. R. Bhowmik , S. Gangopadhyay , P. K. Gangopadhyay , J. Coord. Chem. 2005, 58, 795.

[46] S. Sadegh , S. S. Movafagh , Comments Inorg. Chem. 2025, 00, 1.

[47] T. Allman , R. Goel , Can. J. Chem. 1982, 60, 716.

[48] A. Guerriero , A. Ienco , T. Hicks , A. Cilibrizzi , RSC Adv. 2024, 14, 21139. 38966814 10.1039/d4ra02877e PMC11223513

[49] A. Guerriero , L. Gonsalvi , Inorg. Chim. Acta 2021, 518, 1.

[50] A. Kręzel , R. Latajka , G. D. Bujacz , W. Bal , Inorg. Chem. 2003, 42, 1994. 12639134 10.1021/ic025969y

[51] M. V. Dobrynin , E. V. Sokolova , M. A. Kinzhalov , A. S. Smirnov , G. L. Starova , V. Y. Kukushkin , R. M. Islamova , ACS Appl. Polym. Mater. 2021, 3, 857.

[52] J. Hu , M. Nikravesh , H. R. Shahsavari , R. B. Aghakhanpour , A. L. Rheingold , M. Alshami , Y. Sakamaki , H. Beyzavi , Inorg. Chem. 2020, 59, 16319. 33135890 10.1021/acs.inorgchem.0c02115 PMC7845769

[53] M. A. Ivanov , M. V. Puzyk , Russ. J. Gen. Chem. 2001, 71, 1660.

[54] I. S. Oliveira , M. S. A. Garcia , N. M. Cassani , A. L. C. Oliveira , L. C. F. Freitas , V. K. S. Bertolini , J. Castro , G. Clauss , J. Honorato , F. R. Gadelha , D. C. Miguel , A. C. G. Jardim , C. Abbehausen , Dalt. Trans. 2024, 53, 18963. 10.1039/d4dt01879f 39171417

[55] V. Cepeda , M. Fuertes , J. Castilla , C. Alonso , C. Quevedo , J. Perez , Anticancer. Agents Med. Chem. 2008, 7, 3. 10.2174/187152007779314044 17266502

[56] OECD/OCDE, Giudance 1995, 107, 1.

[57] A. L. de A. Querino , K. B. Enes , O. A. Chaves , D. Dittz , M. R. C. Couri , R. Diniz , H. Silva , Bioorg. Chem. 2020, 100, 1. 10.1016/j.bioorg.2020.103936 32438131

[58] A. Tarushi , X. Totta , A. Papadopoulos , J. Kljun , I. Turel , D. P. Kessissoglou , G. Psomas , Eur. J. Med. Chem. 2014, 74, 187. 24463435 10.1016/j.ejmech.2013.12.019

[59] A. Ramezanpour , K. Karami , M. Kharaziha , M. Zakariazadeh , J. Lipkowski , A. Shahpiri , N. Azizi , M. Namazian , Polyhedron 2021, 206, 1.

[60] L. Zarei , Z. Asadi , V. Eigner , M. Dusek , J. Mol. Struct. 2020, 1200, 1.

[61] V. K. Singh , H. Mishra , R. Ali , S. Umrao , R. Srivastava , S. Abraham , A. Misra , V. N. Singh , H. Mishra , R. S. Tiwari , A. Srivastava , ACS Appl. Nano Mater. 2019, 2, 566.

[62] S. Pandit , S. Kundu , Colloids Surfaces A Physicochem. Eng. Asp. 2021, 628, 1.

[63] P. L. Gentili , F. Ortica , G. Favaro , J. Phys. Chem. B. 2008, 112, 16793. 19367911 10.1021/jp805922g

[64] A. Heidari , H. Mansouri‐Torshizi , M. Saeidifar , K. Abdi , Bull. Chem. Soc. Jpn. 2021, 94, 2678.

[65] S. Hashemi , K. Karami , Z. S. Dehkordi , A. A. Momtazi‐Borojeni , S. A. Esmaeili , J. Biomol. Struct. Dyn. 2022, 40, 5000. 33356950 10.1080/07391102.2020.1865202

[66] J. Ghatge , S. T. Nandibewoor , S. D. Joshi , J. Photochem. Photobiol. A Chem. 2024, 451, 1.

[67] F. Jozefíková , S. Perontsis , K. Koňáriková , L. Švorc , M. Mazúr , G. Psomas , J. Moncol , J. Inorg. Biochem. 2022, 228, 1. 10.1016/j.jinorgbio.2021.111696 35030390

[68] A. Dey , R. Kumar , B. Dutta , R. Bandopadhyay , S. Chakrabortty , M. A. Khan , B. H. Jeon , A. K. Ghosh , ACS Omega 2023, 8, 45653. 38075834 10.1021/acsomega.3c05944 PMC10701725

[69] L. Tabrizi , H. Chiniforoshan , H. Tavakol , Spectrochim. Acta—Part A Mol. Biomol. Spectrosc. 2015, 141, 16. 10.1016/j.saa.2015.01.027 25659738

[70] A. L. de Andrade Querino , J. T. da Silva , J. T. Silva , G. M. Alvarenga , C. H. da Silveira , M. T. Q. de Magalhães , O. A. Chaves , B. A. Iglesias , R. Diniz , H. Silva , J. Biol. Inorg. Chem. 2019, 24, 1087. 31620894 10.1007/s00775-019-01719-5

[71] M. dos S. Marinho , Y. N. Zhang , N. M. Cassani , I. A. Santos , A. L. C. Oliveira , A. K. dos Santos Pereira , P. P. Corbi , B. Zhang , A. C. G. Jardim , Heliyon 2024, 10, 1. 10.1016/j.heliyon.2024.e33885 PMC11283106 39071632

[72] F. S. Varghese , K. Rausalu , M. Hakanen , S. Saul , B. M. Kümmerer , P. Susi , A. Merits , T. Ahola , Antimicrob. Agents Chemother. 2017, 61, 1. 10.1128/AAC.02227-16 PMC5328557 27993855

[73] S. Kuivanen , M. M. Bespalov , J. Nandania , A. Ianevski , V. Velagapudi , J. K. De Brabander , D. E. Kainov , O. Vapalahti , Antiviral Res. 2017, 139, 117. 28049006 10.1016/j.antiviral.2016.12.022

[74] S. Silva , J. F. Shimizu , D. M. de Oliveira , L. R. de Assis , C. Bittar , M. Mottin , B. K. de P. Sousa , N. C. de M. R. Mesquita , L. O. Regasini , P. Rahal , G. Oliva , A. L. Perryman , S. Ekins , C. H. Andrade , L. R. Goulart , R. Sabino‐Silva , A. Merits , M. Harris , A. C. G. Jardim , Sci. Rep. 2019, 9, 1. 31776405 10.1038/s41598-019-54169-z PMC6881388

[75] M. S. A. Garcia , V. A. O. Filho , M. B. C. Brioschi , K. Minori , D. C. Miguel , Trends Parasitol. 2025, 41, 66. 39572329 10.1016/j.pt.2024.10.023

[76] M. Echeverri , A. Alvarez‐Valdés , F. Navas , J. Perles , I. Sánchez‐Pérez , A. Quiroga , R. Soc. Open Sci. 2018, 5, 1. 10.1098/rsos.171340 PMC5830740 29515851

[77] F. Neese , F. Wennmohs , U. Becker , C. Riplinger , J. Chem. Phys. 2020, 152, 1. 10.1063/5.0004608 32534543

[78] F. Neese , Wiley Interdiscip. Rev. Comput. Mol. Sci. 2022, 12, 1.

[79] M. Ernzerhof , G. E. Scuseria , J. Chem. Phys. 1999, 110, 5029.

[80] N. Bartlett , Gold Bull. 1998, 31, 22.

[81] E. Lenthe , R. Leeuwen , E. Baerends , J. Snijders , Int. J. Quantum Chem. 1996, 57, 281.

[82] S. Kossmann , F. Neese , J. Chem. Theory Comput. 2010, 6, 2325. 26613489 10.1021/ct100199k

[83] M. Garcia‐Ratés , F. Neese , J. Comput. Chem. 2020, 41, 922. 31889331 10.1002/jcc.26139

[84] M. Garcia‐Ratés , F. Neese , J. Comput. Chem. 2019, 40, 1816. 30938846 10.1002/jcc.25833

[85] G. Dilabio , D. Pratt , A. Lofaro , J. Wright , J. Phys. Chem. 1999, 103, 1653.

[86] C. M. Teles , V. U. Antunes , R. S. Cardoso , T. Z. Candido , C. S. P. Lima , A. L. T. G. Ruiz , M. A. Juliano , D. C. Favaro , C. Abbehausen , Inorg. Chim. Acta 2020, 511, 1.

[87] M. Feizi‐Dehnayebi , E. Dehghanian , H. Mansouri‐Torshizi , J. Mol. Struct. 2021, 1240, 1. 10.1016/j.saa.2020.119215 33262078

[88] I. V. Tetko , H. P. Varbanov , M. S. Galanski , M. Talmaciu , J. A. Platts , M. Ravera , E. Gabano , J. Inorg. Biochem. 2016, 156, 1. 26717258 10.1016/j.jinorgbio.2015.12.006

[89] S. Jagadeesan , V. Balasubramanian , P. Baumann , M. Neuburger , D. Häussinger , C. G. Palivan , Inorg. Chem. 2013, 52, 12535. 24127683 10.1021/ic4016228

[90] M. Klose , S. Theiner , H. Varbanov , D. Hoefer , V. Pichler , M. Galanski , S. Meier‐Menches , B. Keppler , Inorganics 2018, 6, 1.

[91] M. Almendros , M. François‐heude , L. Legentil 2013, 2013, 123.

[92] C. L. Donald , B. Brennan , S. L. Cumberworth , V. V. Rezelj , J. J. Clark , M. T. Cordeiro , R. F. de Oliveira França , L. J. Pena , G. S. Wilkie , A. Da Silva Filipe , C. Davis , J. Hughes , M. Varjak , M. Selinger , L. Zuvanov , A. M. Owsianka , A. H. Patel , J. McLauchlan , B. D. Lindenbach , G. Fall , A. A. Sall , R. Biek , J. Rehwinkel , E. Schnettler , A. Kohl , PLoS Negl. Trop. Dis. 2016, 10, 1. 10.1371/journal.pntd.0005048 PMC5051680 27706161

[93] Y. Gao , Y. Wu , Int. J. Biol. Macromol. 2022, 203, 379. 35104473 10.1016/j.ijbiomac.2022.01.162

[94] V. O. Filho , M. Garcia , L. Rosa , S. Giorgio , D. Miguel , Parasitologia 2024, 4, 305.

[95] T. L. Riss , R. A. Moravec , A. L. Niles , S. Duellman , H. A. Benink , T. J. Worzella , L. Minor , Cell Viability Assays, in Assay Guidance Manual 2013 (Updated 2016).

[96] P. Kumar , A. Nagarajan , P. D. Uchil , Cold Spring Harb. Protoc. 2018, 2018, 469. 10.1101/pdb.top096222 30510131

