# Supplementary material for: Monodentate Phosphine Modulation in Cyclometallated Platinum(II) Complexes for Antileishmanial, Antiviral, and Antitumor Applications
Source: ChemMedChem. 2025 Dec 24;21(4):e202500782. doi: 10.1002/cmdc.202500782 (PMC12913238; doi:10.1002/cmdc.202500782)
Supplement: Supplementary file 1 — Supplementary Material [file CMDC-21-e202500782-s001.pdf]

# Supporting Information

# Monodentate Phosphine Modulation in Cyclometallated Platinum(II) Complexes for Antileishmanial, Antiviral and Antitumor Applications

*Antonio A. de Oliveira-Neto*[a], *Gustavo Clauss*[a], *Jennyfer Castro*[a], *Marcus S. A. Garcia*[b], *Natasha M. Cassani*[c], *Bruna C. Sandim*[c], *Ana Laura C. Oliveira*[c], *Stephanie P. B. Reyes*[c], *Nádija N.P. da Silva*[d], *Fillipe V. Rocha*[d], *Ana C. G. Jardim*[c] *Danilo C. Miguel*[b] *Camilla Abbehausen*[a]*

*a Institute of Chemistry, University of Campinas, Campinas, São Paulo, Brazil*

*b Institute of Biology, University of Campinas, São Paulo, Brazil*

*c Laboratory of Antiviral Research (LAPAV), Institute of Biomedical Sciences, Federal University of Uberlândia, Uberlândia, Minas Gerais, Brazil*

*d Department of Chemistry, Federal University of São Carlos, São Paulo, Brazil*

*Corresponding author: camilla@unicamp.br*

**Table of contents**

$^{1}H$ NMR spectra of the five complexes------------------------------------------------------------------------------3

$^{13}C$ NMR spectra of the five complexes and $^{1}H$-$^{1}H$ COSY NMR of complex 5------------------------------4

$^{31}P$ NMR spectra of complexes 2–5 and TCEP------------------------------------------------------------------5

IR spectra of the five complexes and their precursor ligands ----------------------------------------------------6

FAR-IR spectra of the five complexes and their precursor ligands -------------------------------------------7

Raman spectra of complexes 1–4 and their assignments. Theoretical assignments and IR spectra assignments of the five complexes------------------------------------------------------------------------------------8

Mass spectra of the five complexes-------------------------------------------------------------------------------------9

Thermogravimetric analysis (TGA) curves of the five complexes------------------------------------------10

UV–Vis spectra of the five complexes and their assignments. HOMO and LUMO of complex 5-------11

Chloride exchange by DMSO in the five complexes----------------------------------------------------------12

Titration of the five complexes with Nac-----------------------------------------------------------------------13

Interaction of the five complexes with His---------------------------------------------------------------------15

Mass spectra of the mixture of Complex 3 with His----------------------------------------------------------16

Calibration curve and log P of the five complexes------------------------------------------------------------17

Emission spectra of the five complexes and BSA excited at 280 nm----------------------------------------18

Interaction of the five complexes with BSA--------------------------------------------------------------------19

Emission spectra of all free complexes and in the presence of CT-DNA, as well as of the CT-DNA–EB adduct, excited at 540 nm ---------------------------------------------------------------------------------------21

Fluorescence quenching of the CT-DNA–EB adduct, excited at 540 nm, in the presence of each complex------------------------------------------------------------------------------------------------------------22

Fluorescence titration experiment of Complex 3 with His---------------------------------------------------24

Fluorescence of Complex 3 + His (1:15) in acidic buffer at pH 5.2 and in acidic medium at pH 5.2----24

Fluorescence spectra of Complex 3 in the presence of BSA (1:1) upon excitation at 321 nm-----------25

References-----------------------------------------------------------------------------------------------------------25

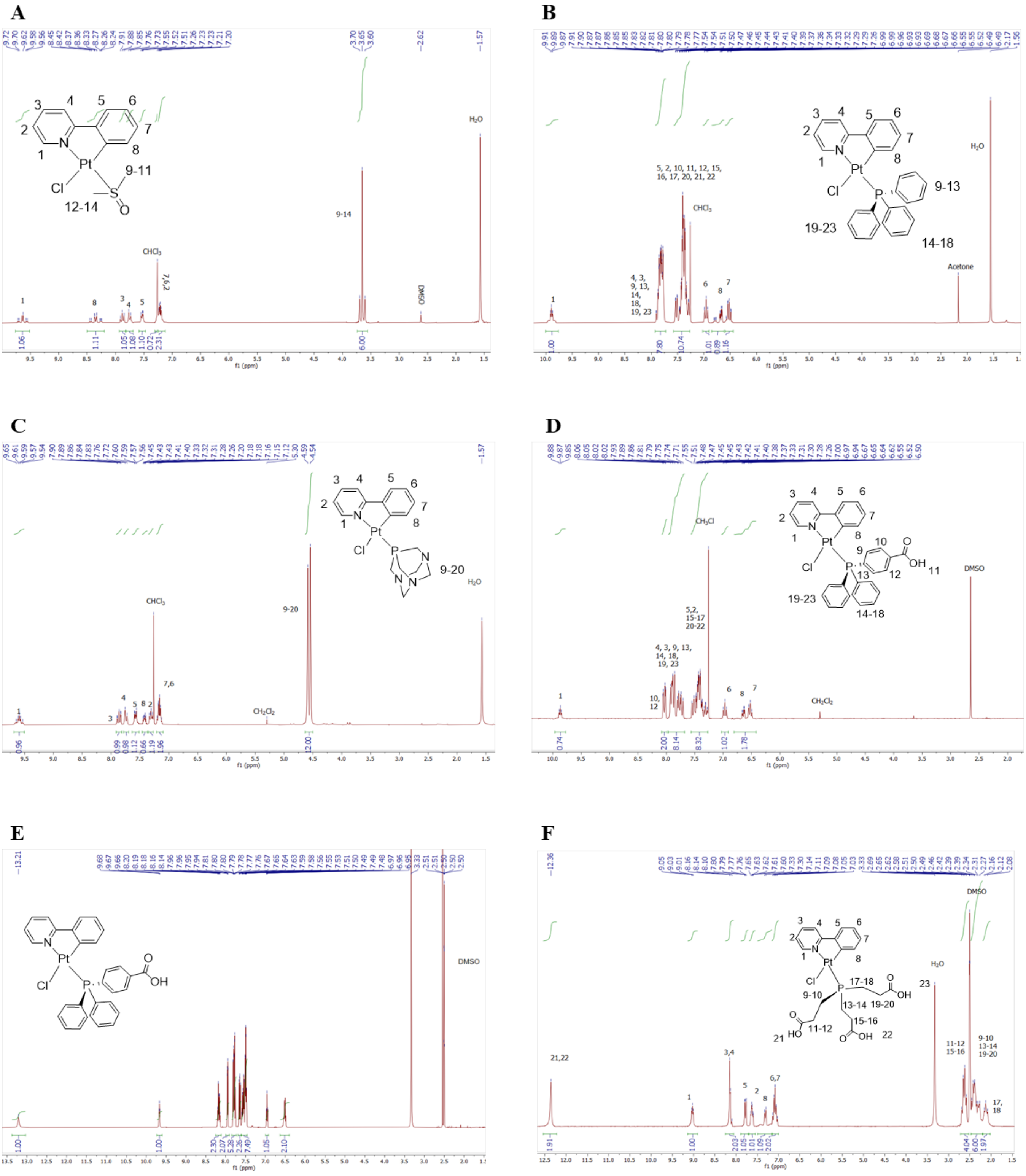


Figure S1: **A-D** $^{1}$H NMR spectra Complex 1-4 (250 MHz; $CDCl_3$) δ in ppm. **E**. $^{1}$H NMR spectra Complex 4 (500 MHz; DMSO-d$^{6}$) δ in ppm. **F** $^{1}$H NMR spectra Complex 5 (250 MHz; DMSO-d$^{6}$) δ in ppm.

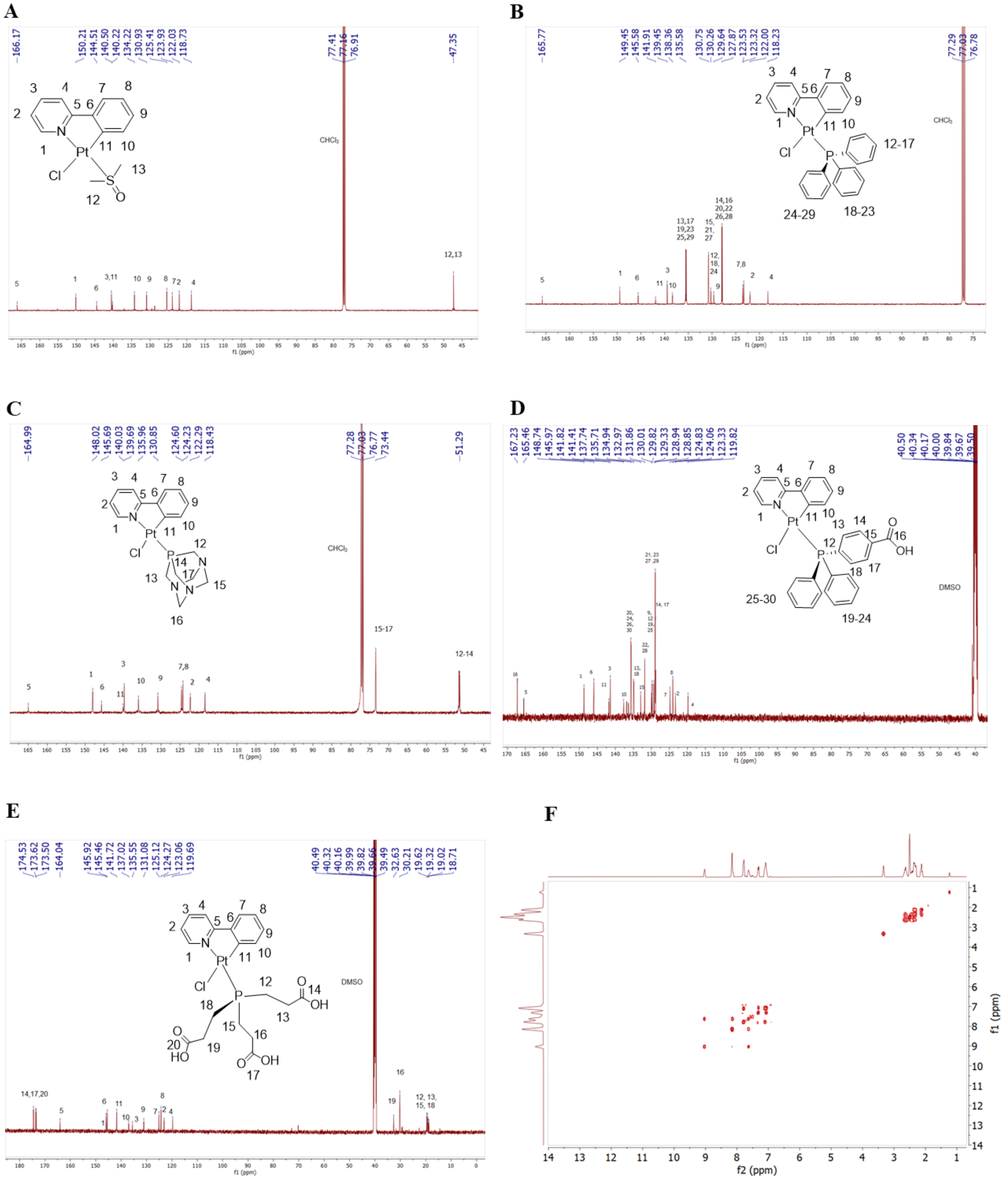


Figure S2: **A-C.** $^{13}$C NMR spectra Complex 1-3 (125 MHz; $CDCl_3$) δ in ppm. **D-E.** $^{13}$C NMR spectra Complex 4 and 5 (125 MHz; DMSO-d$^6$) δ in ppm. **F.** $^1$H-$^1$H COSY NMR Complex 5 (500 Mhz; DMSO-d$^6$).

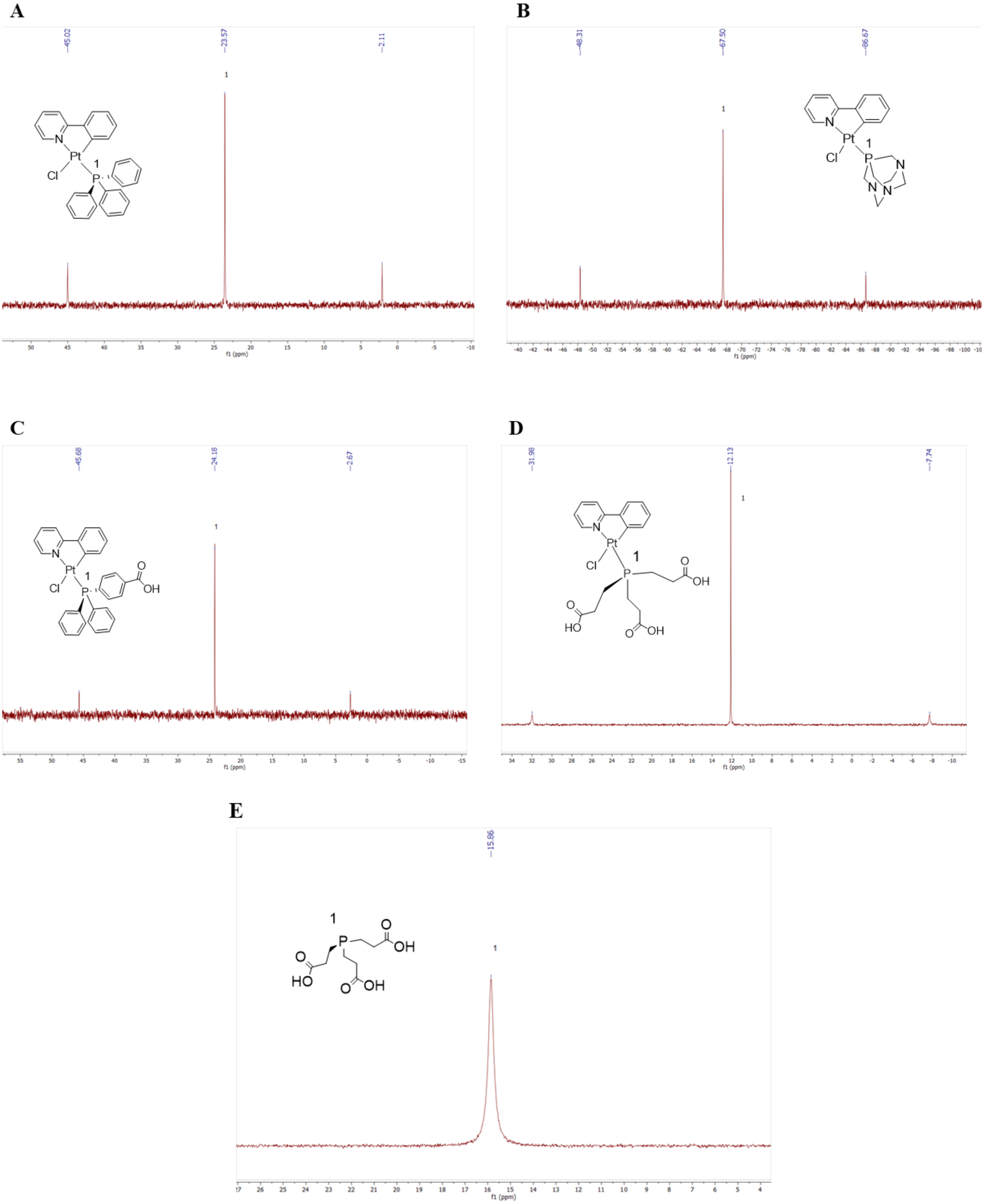


Figure S3: **A-C.** $^{31}$P NMR spectra Complex 2-4 (101.25 MHz; $CDCl_3$) δ in ppm. **D**. $^{31}$P NMR spectra Complex 5 (101.25 Mhz; DMSO-d$^6$) δ in ppm. **E.** $^{31}$P NMR spectra TCEP (101.25 Mhz; $D_2O$) δ in ppm.

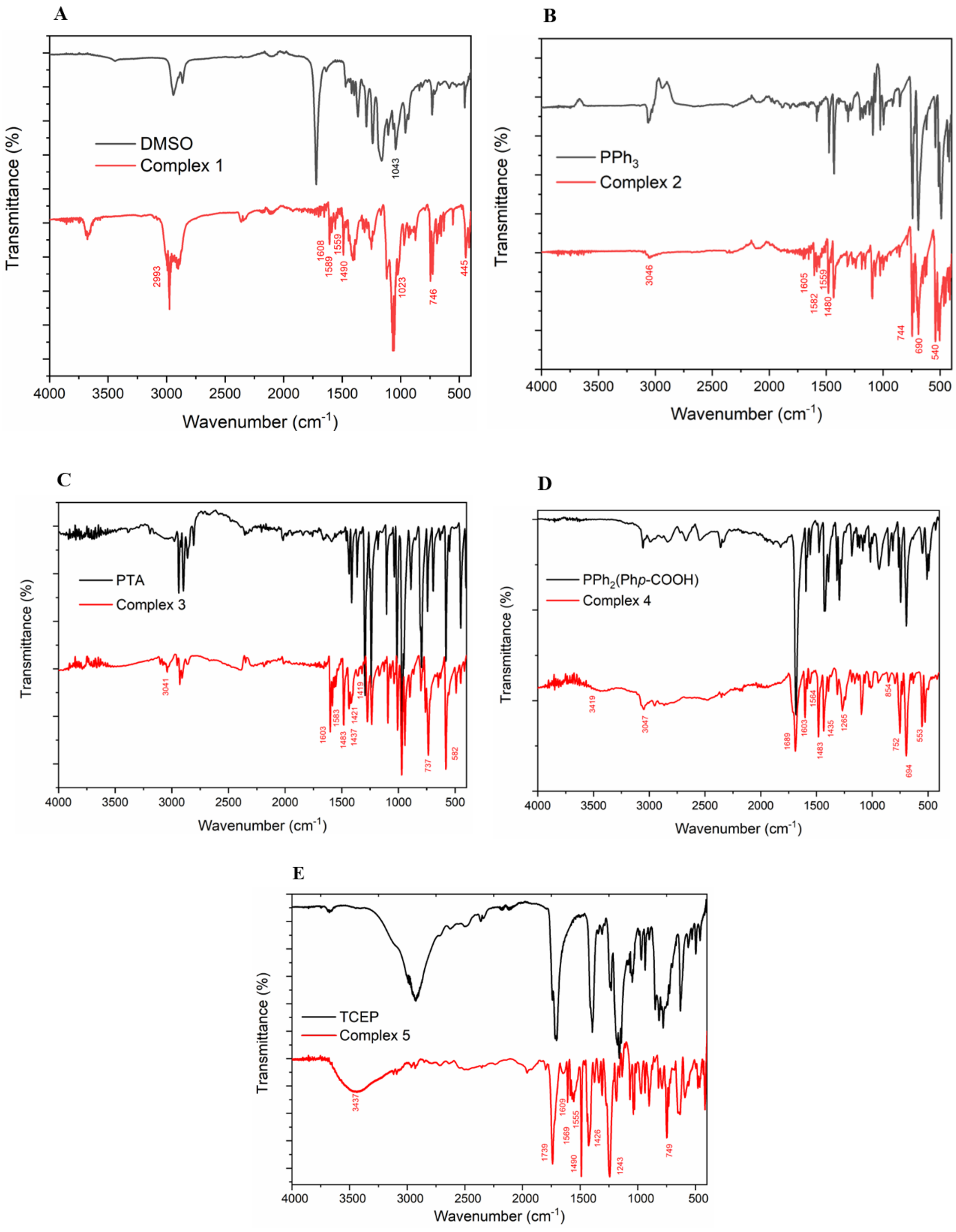


Figure S4: **A-E**. IR spectra of 1-5 Complexes and their precursor ligands.

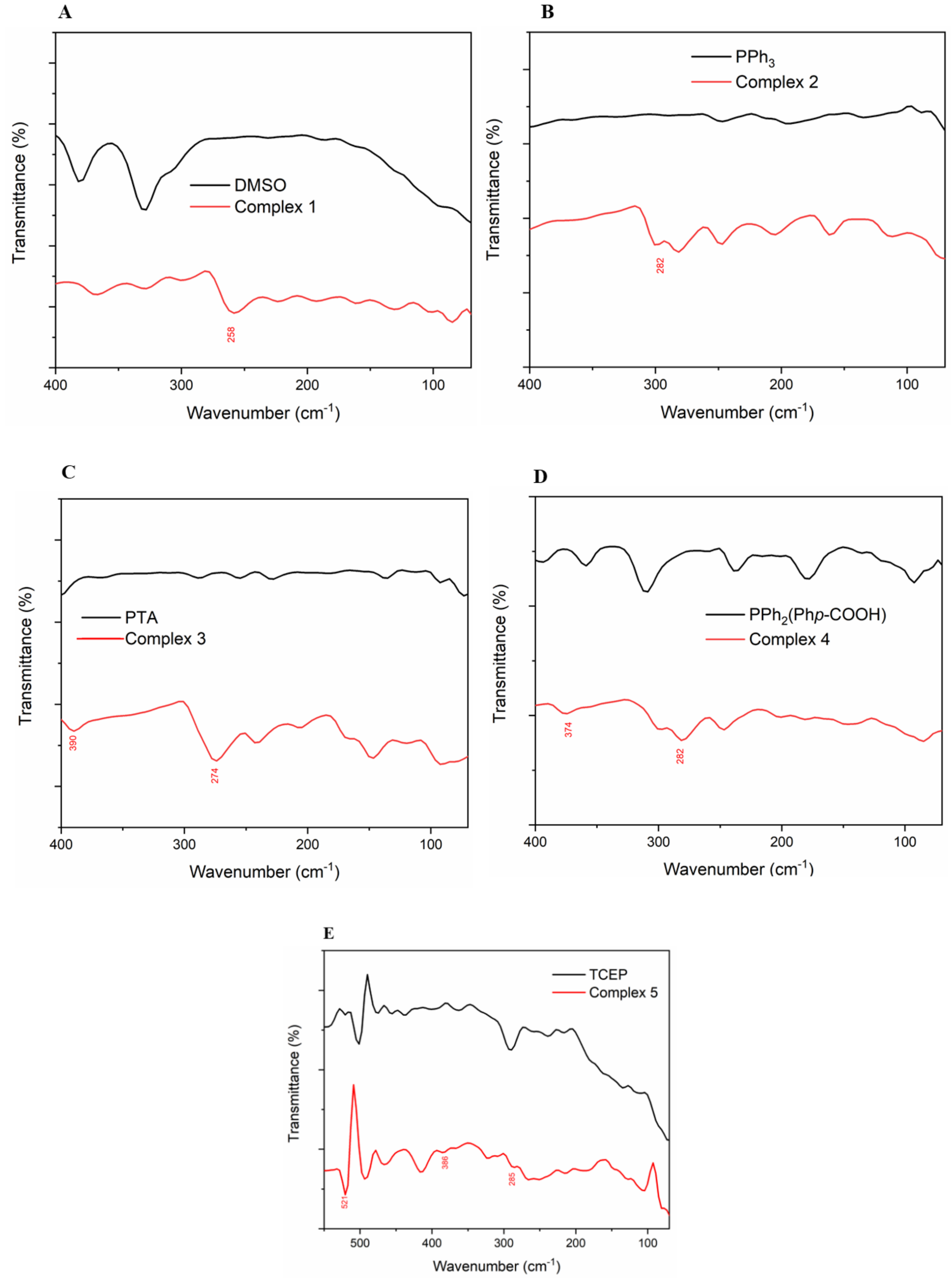


Figure S5: **A-E.** FAR-IR spectra of 1-5 Complexes and their precursor ligands.

**A**

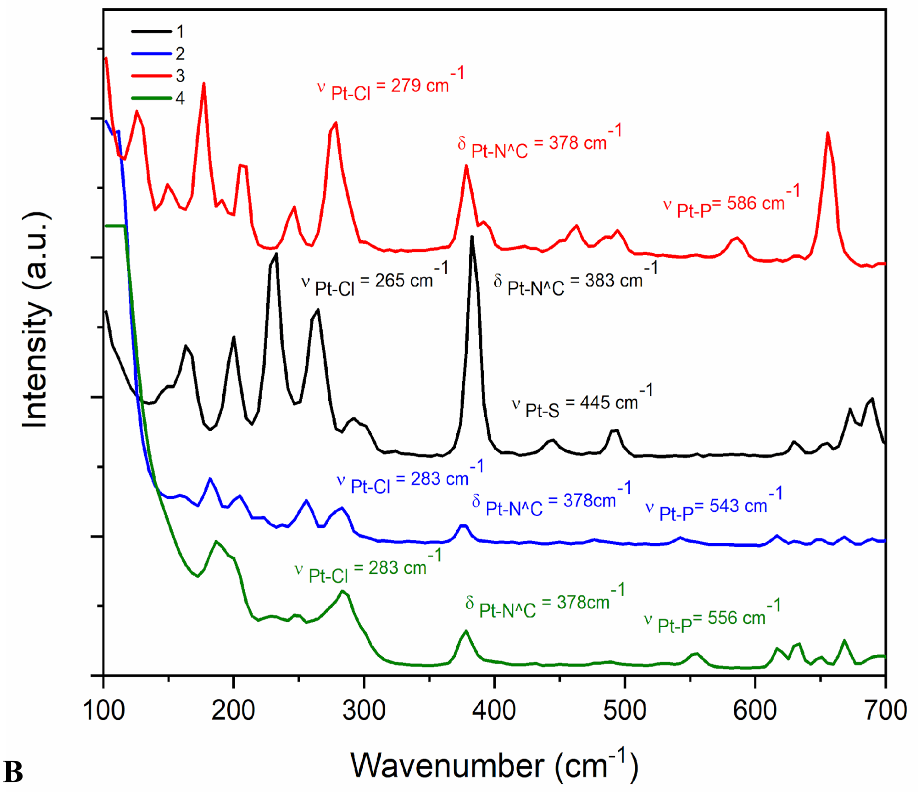


**B**

| Complex | δ Pt-N^C (cm$^{-1}$) | | | υ Pt-P (cm$^{-1}$) | | |
|---|---|---|---|---|---|---|
| | **Theoretical** | **IV** | **RAMAN** | **Theoretical** | **IV** | **RAMAN** |
| 1 | 387 | - | 383 | - | - | - |
| 2 | 385 | - | 378 | 548 | 540 | 543 |
| 3 | 384 | 390 | 392 | 588 | 582 | 586 |
| 4 | 384 | 374 | 378 | 547 | 553 | 556 |
| 5 | 383 | - | - | 526 | 521 | - |
| **Complex** | **υ Pt-Cl (cm$^{-1}$)** | | | **υ Pt-S (cm$^{-1}$)** | | |
| | **Theoretical** | **IV** | **RAMAN** | **Theoretical** | **IV** | **RAMAN** |
| 1 | 265 | 258 | 265 | 449 | 445 | 445 |
| 2 | 281 | 282 | 283 | - | - | - |
| 3 | 277 | 274 | 279 | - | - | - |
| 4 | 280 | 282 | 283 | - | - | - |
| 5 | 289 | 285 | - | - | - | - |

Figure S6: **A.** RAMAN spectra of 1-4 Complexes. **B** Theoretical and experimental assignments (IR and Raman) of the stretching vibrations of complexes 1–5 in cm$^{-1}$. Assignments were based on DFT vibrational analysis.[1,2]

**A**

Comparison between the real spectrum (top) and the simulated one (bottom) for $C_{13}H_{14}NOPtS^{+}$

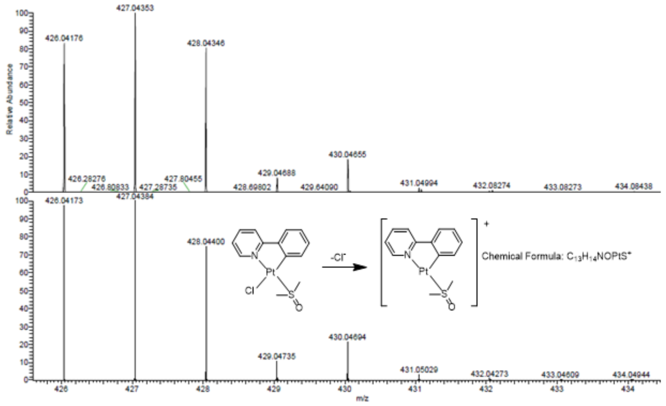


$[M - Cl]^{+} = 427.04384$ m/z

**B**

Comparison between the real spectrum (top) and the simulated one (bottom) for $C_{29}H_{23}NPPt^{+}$

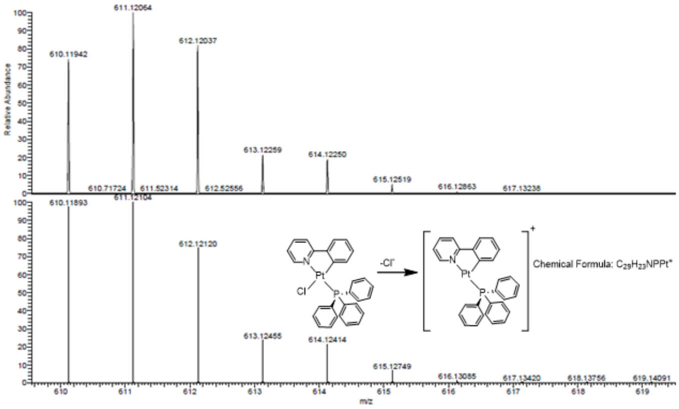


$[M - Cl]^{+} = 611.12104$ m/z

**C**

Comparison between the real spectrum (top) and the simulated one (bottom) for $C_{17}H_{20}N_{4}PPt^{+}$

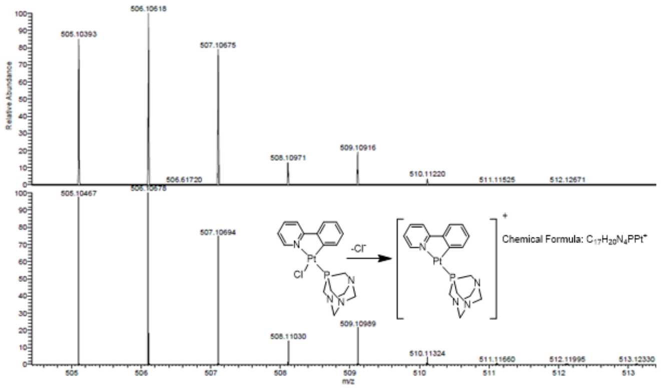


$[M - Cl]^{+} = 506.10678$ m/z

**D**

Comparison between the real spectrum (top) and the simulated one (bottom) for $C_{30}H_{23}NO_{2}PPt^{+}$

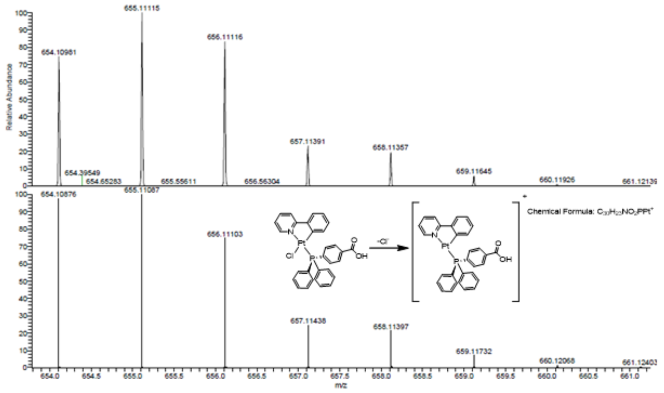


$[M - Cl]^{+} = 655.11087$ m/z

**E**

Comparison between the real spectrum (top) and the simulated one (bottom) for $C_{20}H_{23}NO_{6}PPt^{+}$

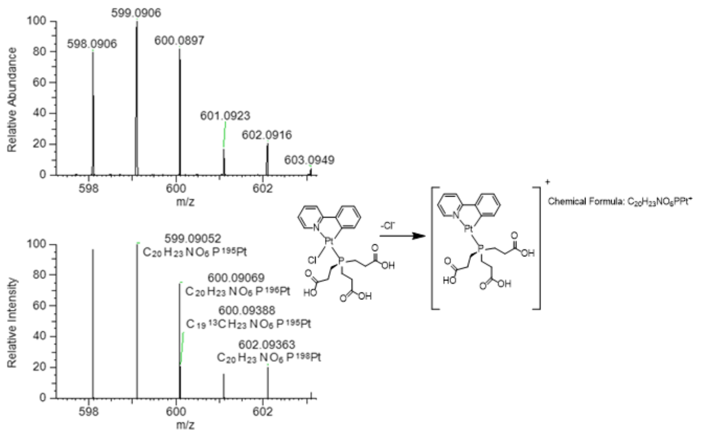


$[M - Cl]^{+} = 599.09052$ m/z

Figure S7: **A-E.** Mass spectra Complexes 1-5 $[M - Cl]^{+}$.

**A**

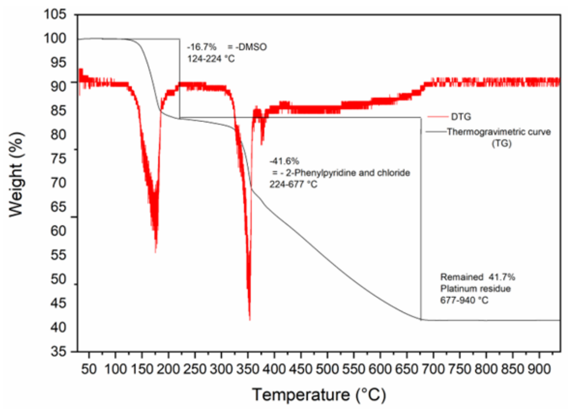


| Mass loss/remaining mass | Theoretical | Experimental | Relative error |
|---|---|---|---|
| **DMSO loss** | 16.9% | 16.7 % | -1.2% |
| **2-phenylpyridine + Chloride loss** | 41.1% | 41.6 % | 1.2% |
| **Platinum residue** | 42.0% | 41.7% | -0.7% |

**B**

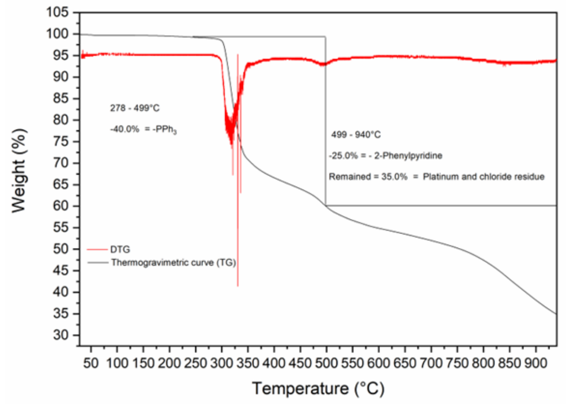


| Mass loss/remaining mass | Theoretical | Experimental | Relative error |
|---|---|---|---|
| **$PPh_3$ loss** | 40.5% | 40.0% | -1.2% |
| **2-phenylpyridine loss** | 24.0 % | 25.0% | 4.2% |
| **Platinum + chloride residue** | 35.5% | 35.0% | -1.4% |

**C**

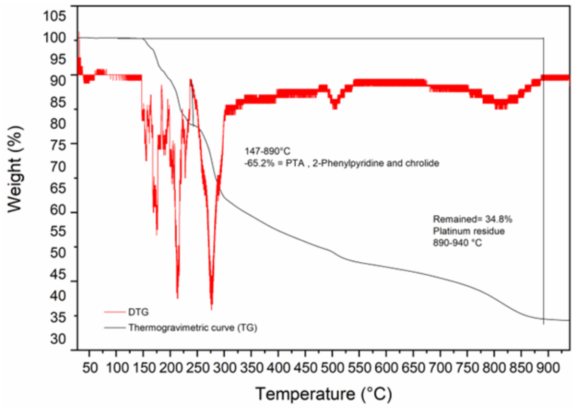


| Mass loss/remaining mass | Theoretical | Experimental | Relative error |
|---|---|---|---|
| **PTA + 2-phenylpyridine + chloride loss** | 64.0% | 65.2% | 1.9% |
| **Platinum residue** | 36.0% | 34.8% | -3.3% |

**D**

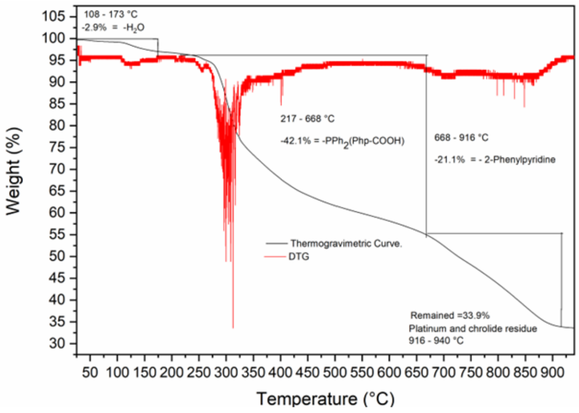


| Mass loss/remaining mass | Theoretical | Experimental | Relative error |
|---|---|---|---|
| **$H_2O$ loss** | 2.5% | 2.9% | 16.0% |
| **$PPh_2$(Php-COOH) loss** | 43.1% | 42.1% | -2.3% |
| **2-phenylpyridine loss** | 21.9% | 21.1% | -3.6% |
| **Platinum and chloride residue** | 32.5% | 33.9% | 4.3% |

**E**

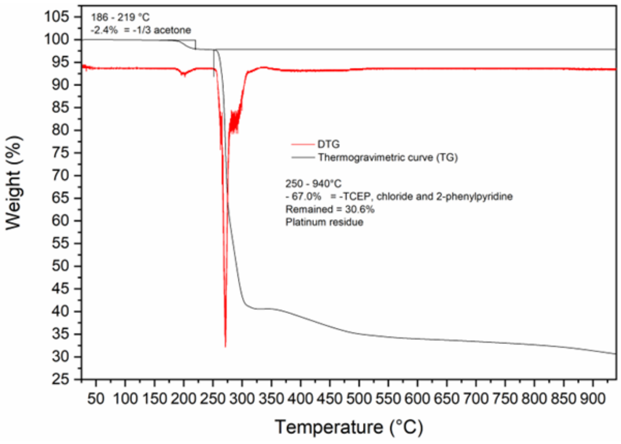


| Mass loss/remaining mass | Theoretical | Experimental | Relative error |
|---|---|---|---|
| **1/3 acetone loss** | 2.9% | 2.4% | -17.2% |
| **TCEP + chloride + 2-phenylpyridine loss** | 67.2% | 67.0% | -0.3% |
| **Platinum residue** | 29.9% | 30.6% | 2.3% |

Figure S8: **A-E.** Thermogravimetric curves (TG), their derivatives (DTG), and the corresponding relative errors of the mass losses for Complexes 1–5.

**A**

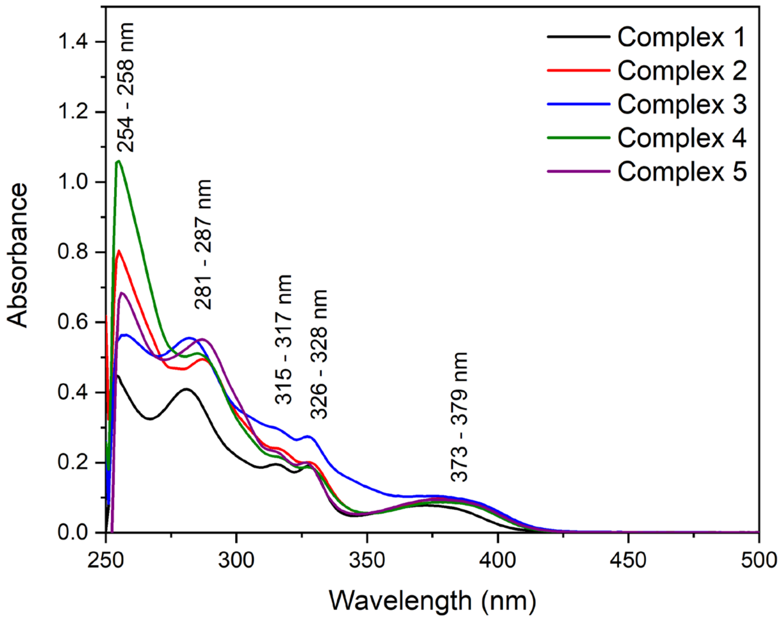


| Wavelength (nm) | ε x $10^{-3}$, $M^{-1}$ $cm^{-1}$ Complex (1; 2; 3) (4 and 5) | Assignment |
|---|---|---|
| 254-258 | (13.9; 24.8; 17.4) ( 32.7 and 20.5) | $_{\pi}$phpy, $_{\pi}$PPh$_3$, $_{\pi}$PPh$_2$(Php-COOH) → $_{\pi}^{*}$phpy, $_{\pi}^{*}$PPh$_3$ and $_{\pi}^{*}$PPh$_2$(Php-COOH) |
| 281-287 | (12.6; 15.3; 17.1) ( 15.8 and 17.0) | $_{\pi}$phpy, $_{\pi}$PPh$_3$, $_{\pi}$PPh$_2$(Php-COOH) → $_{\pi}^{*}$phpy, $_{\pi}^{*}$PPh$_3$, $_{\pi}^{*}$PPh$_2$(Php-COOH) |
| 315-317 | (6.0; 7.3 sh; 9.0 sh) ( 6.7 sh and 7.1 sh) | $_{d}$Pt/$_{\pi}$Cl/$_{\pi}$phpy → $_{\pi}^{*}$phpy, $_{\pi}^{*}$PPh$_3$, $_{\pi}^{*}$PPh$_2$(Php-COOH) |
| 326-328 | (5.9; 6.2; 8.5) ( 5.8 and 6.2) | $_{d}$Pt → $_{\pi}^{*}$phpy |
| 373-379 | (2.4; 3.0; 3.2) (2.7 and 2.9) | $_{d}$Pt/ $_{\pi}$Cl/$_{\pi}$phpy → $_{\pi}^{*}$phpy ( HOMO→ LUMO) |

**B**

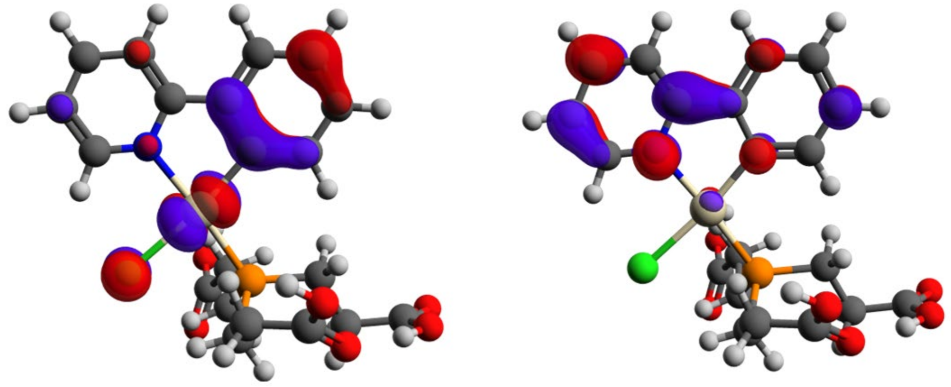

Figure S9: **A**. UV-vis spectra of solutions 3.25 x$10^{-5}$ mol/L of five platinum complexes at DMSO at room temperature and nature of transitions for each complex. $PPh_3$ and $PPh_2$(Php-COOH) are involved only in complexes 2 and 4. **B** HOMO (left) and LUMO (right) of complex 5. [3–6]

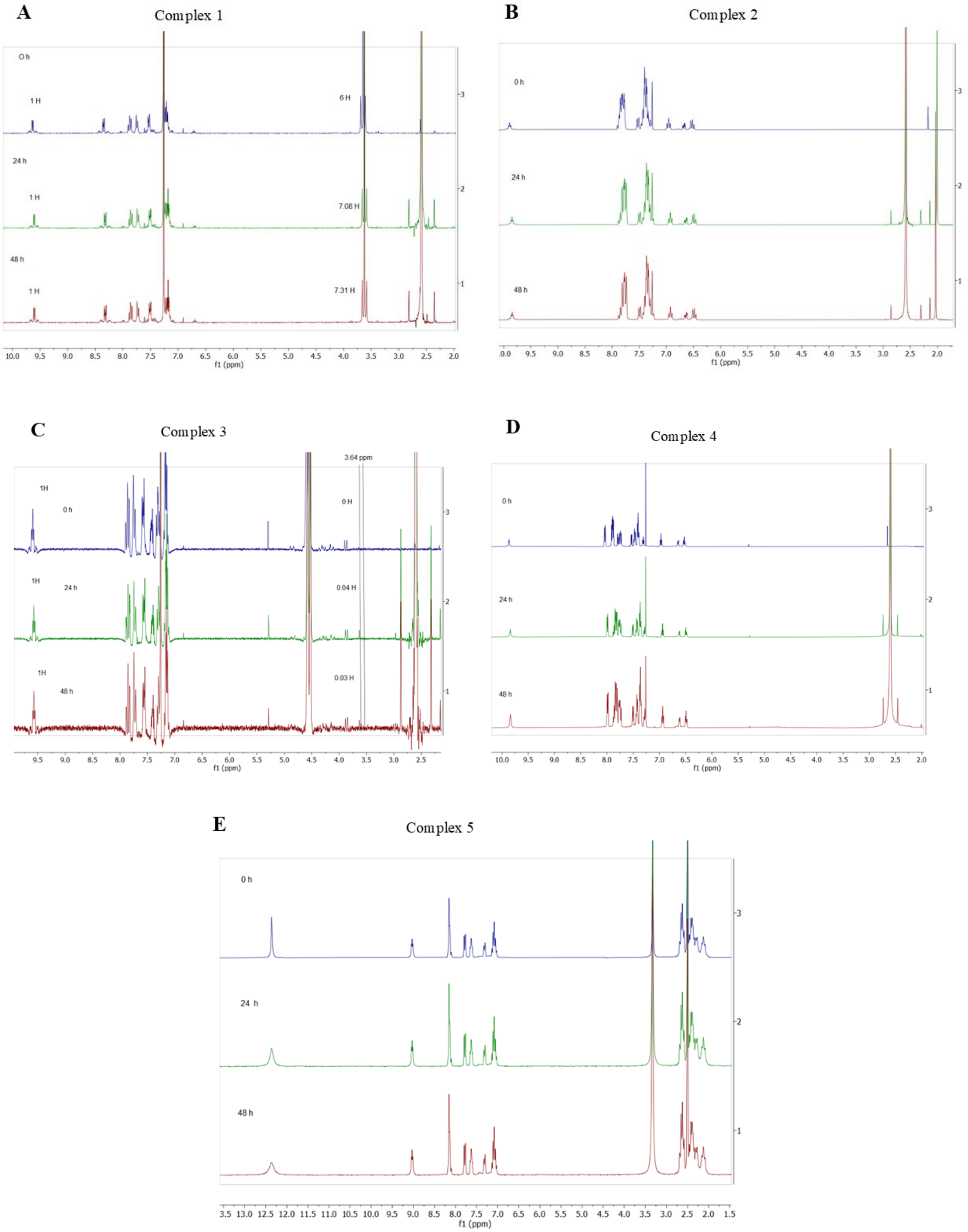


Figure S10: **A.** $^{1}$H NMR spectra Complex 1 without DMSO (0 h) and with DMSO (24 and 48 h) (300 MHz; $CDCl_3$) δ in ppm. **B-D.** $^{1}$H NMR spectra Complex 2-4 without DMSO (0 h) and with DMSO (24 and 48 h) (250 MHz; $CDCl_3$) δ in ppm. **E.** $^{1}$H NMR spectra Complex 5 (0, 24 and 48 h) (250 MHz; DMSO-d$^{6}$) δ in ppm.

**A**

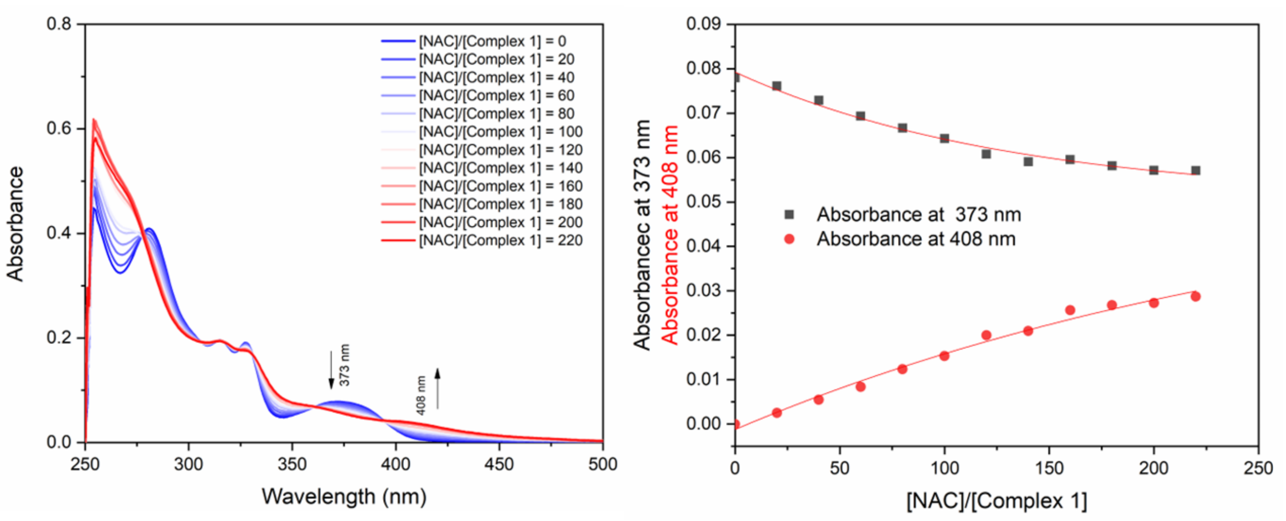


**B**

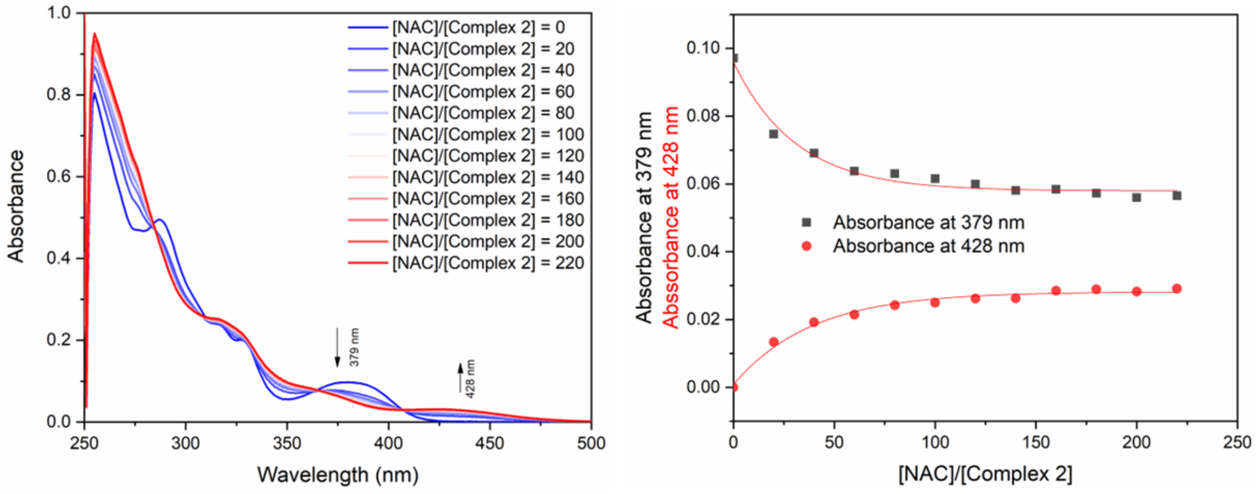


**C**

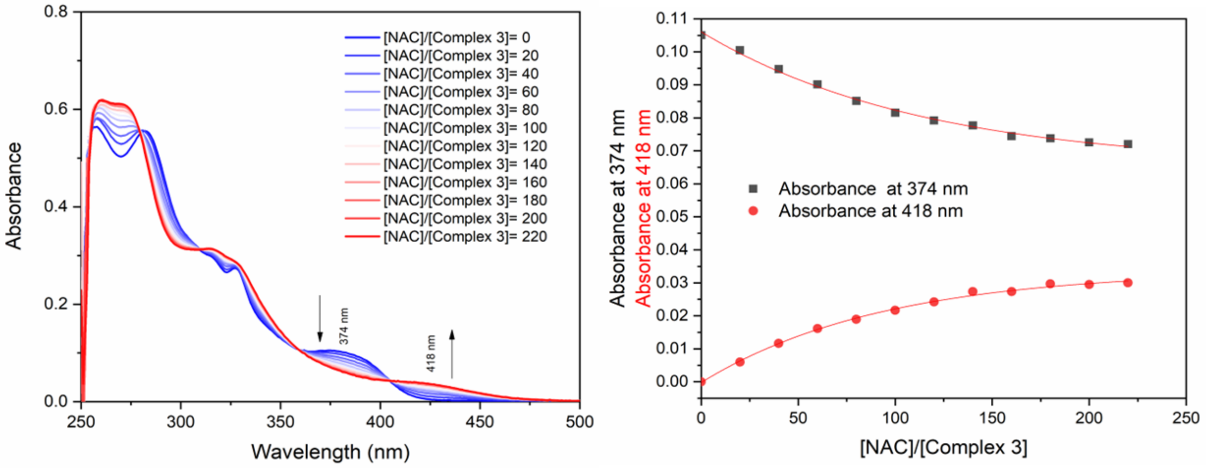


Figure S11: **A-C.** Titration of Complex 1-3 in the presence of Nac. Increase in absorbance of the 408, 428 and 418 bands and decrease in 373, 379 and 374 nm bands.

**A**

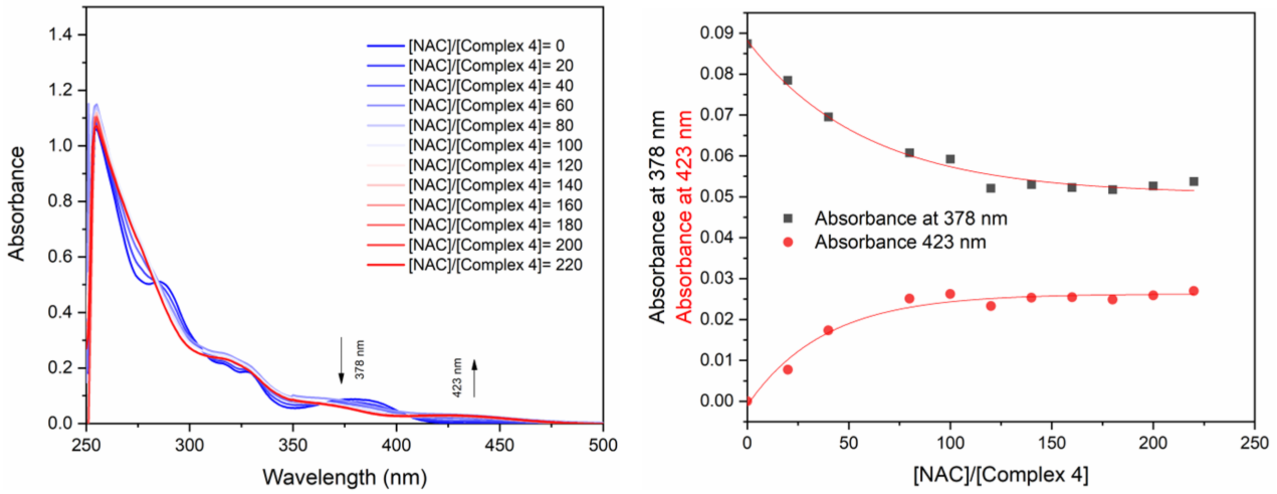


**B**

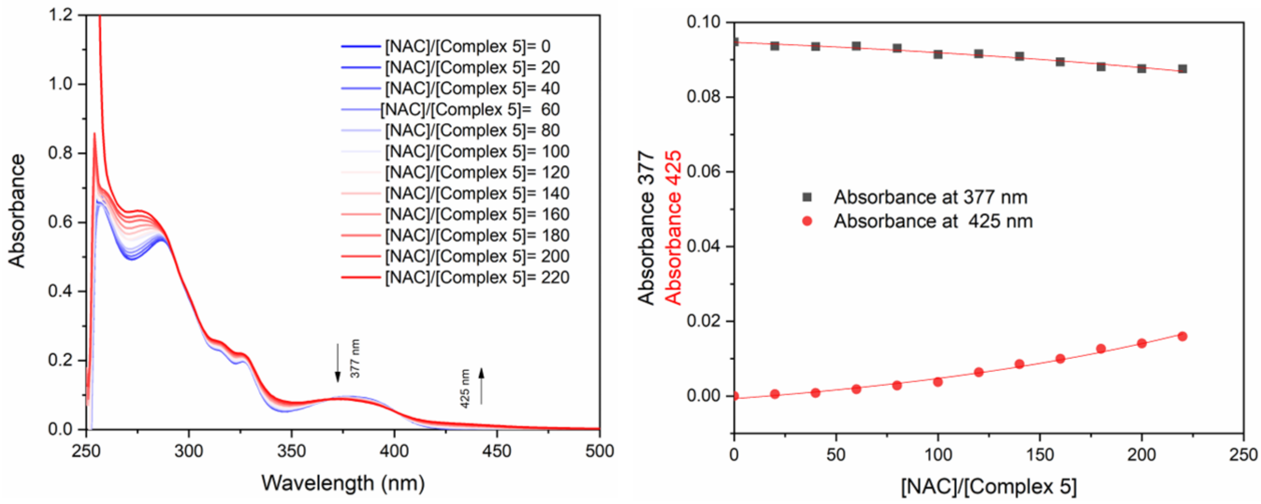


**C**

| Complex | % Complex consumed |
|---|---|
| **1** | 26.8 |
| **2** | 41.8 |
| **3** | 31.4 |
| **4** | 38.6 |
| **5** | 7.6 |

Figure S12: **A-B.** Titration of Complex 4 and 5 in the presence of Nac. Increase in absorbance of the 423 and 425 bands and decrease in 378 and 377 nm bands. **C.** Percentage of Complex 1-5 consumed after [Nac]/[Complex] = 220.

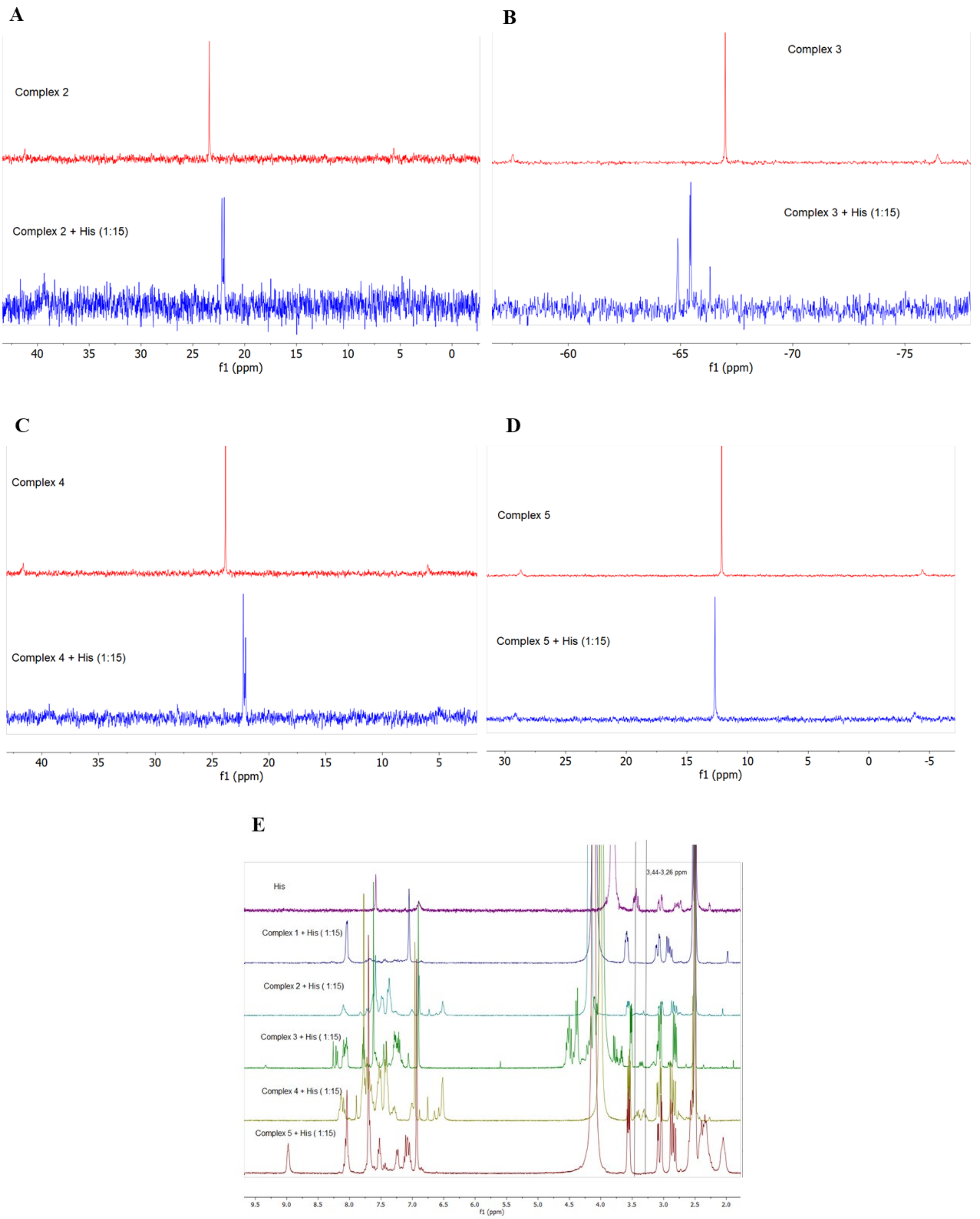


Figure S13: **A, C-D**. $^{31}$P NMR spectra of Complex 2,4-5 in DMSO-d$^6$ and $^{31}$P NMR spectra of Complex 2,4-5 + His (1:15) in DMSO-d$^6$:$D_2O$ 600/100 after 24 hours of His addition (121.50 MHz) δ in ppm. **B.** $^{31}$PNMR spectra of Complex 3 in DMSO-d$^6$ and $^{31}$P NMR spectra of Complex 3 + His (1:15) in DMSO-d$^6$:$D_2O$ 600/100 after 24 hours of His addition (202.50 MHz) δ in ppm. **E.** $^1$H NMR spectra of His and Complex 1-5 + His (1:15) in DMSO-d$^6$:$D_2O$ 600/100 after 24 hours of His addition (300 MHz) δ in ppm.

**A**

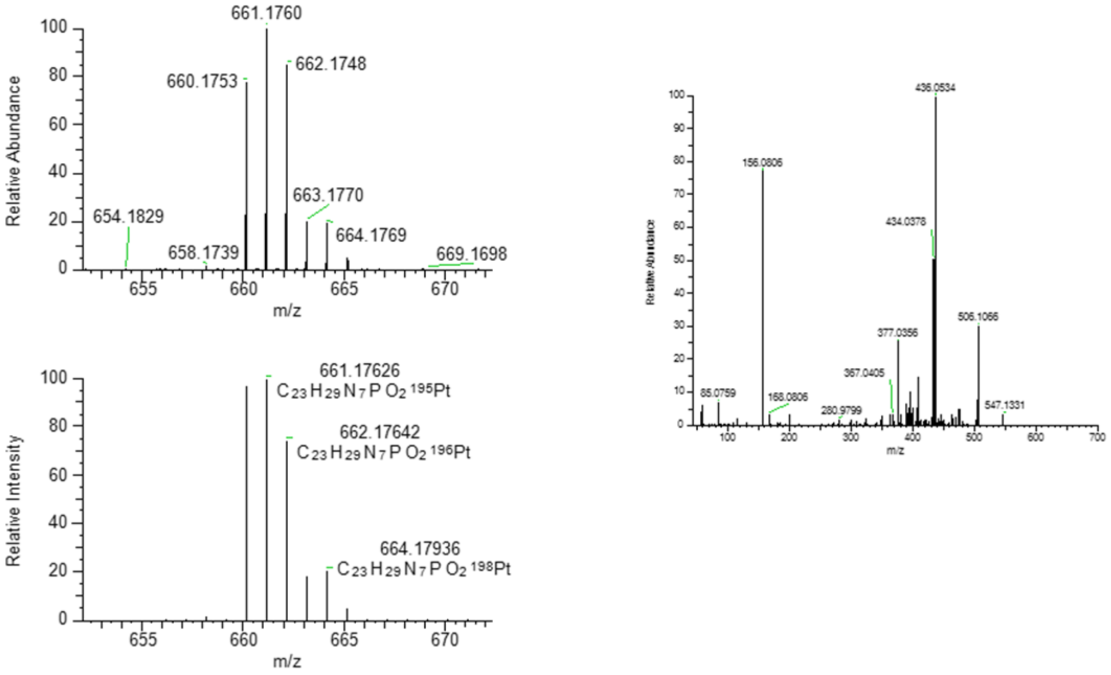


**B**

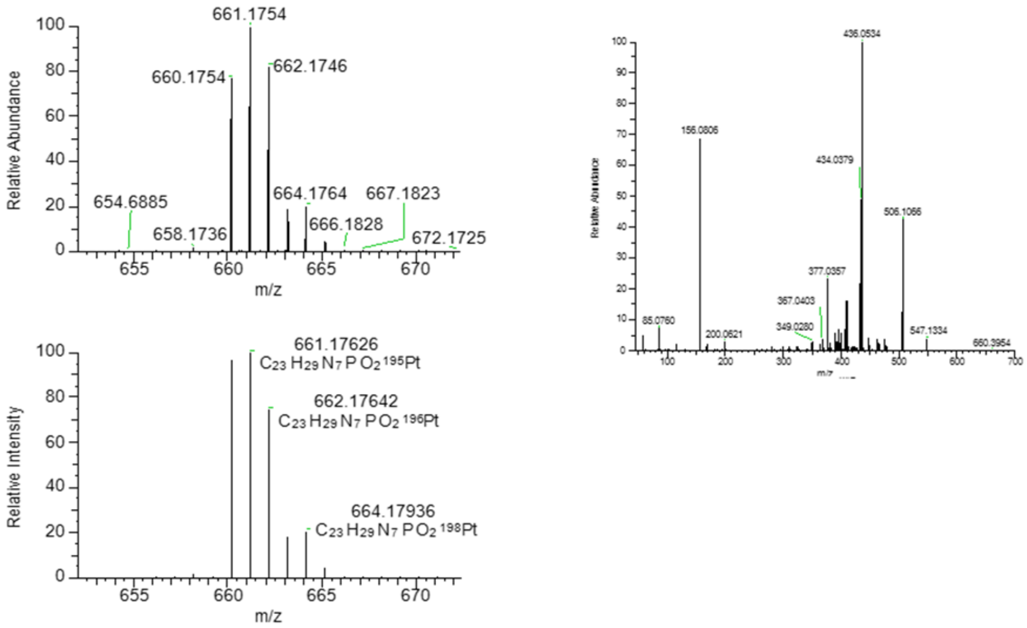


Figure S14: **A.** Comparison of the experimental (top) and simulated (bottom) mass spectra of Complex 3 + Histidine [M–Cl + His]$^{+}$ at a retention time of 8.24 min using a reverse phase, C-18 column, with a water and methanol gradient in positive ion mode, on the left. The corresponding $MS^2$ spectra is shown on the right. **B.** Comparison of the experimental (top) and simulated (bottom) mass spectra of Complex 3 + Histidine [M – Cl + His]$^{+}$ at a retention time of 9.45 min under the same chromatographic conditions in positive ion mode, on the left. The corresponding $MS^2$ spectra is shown on the right.

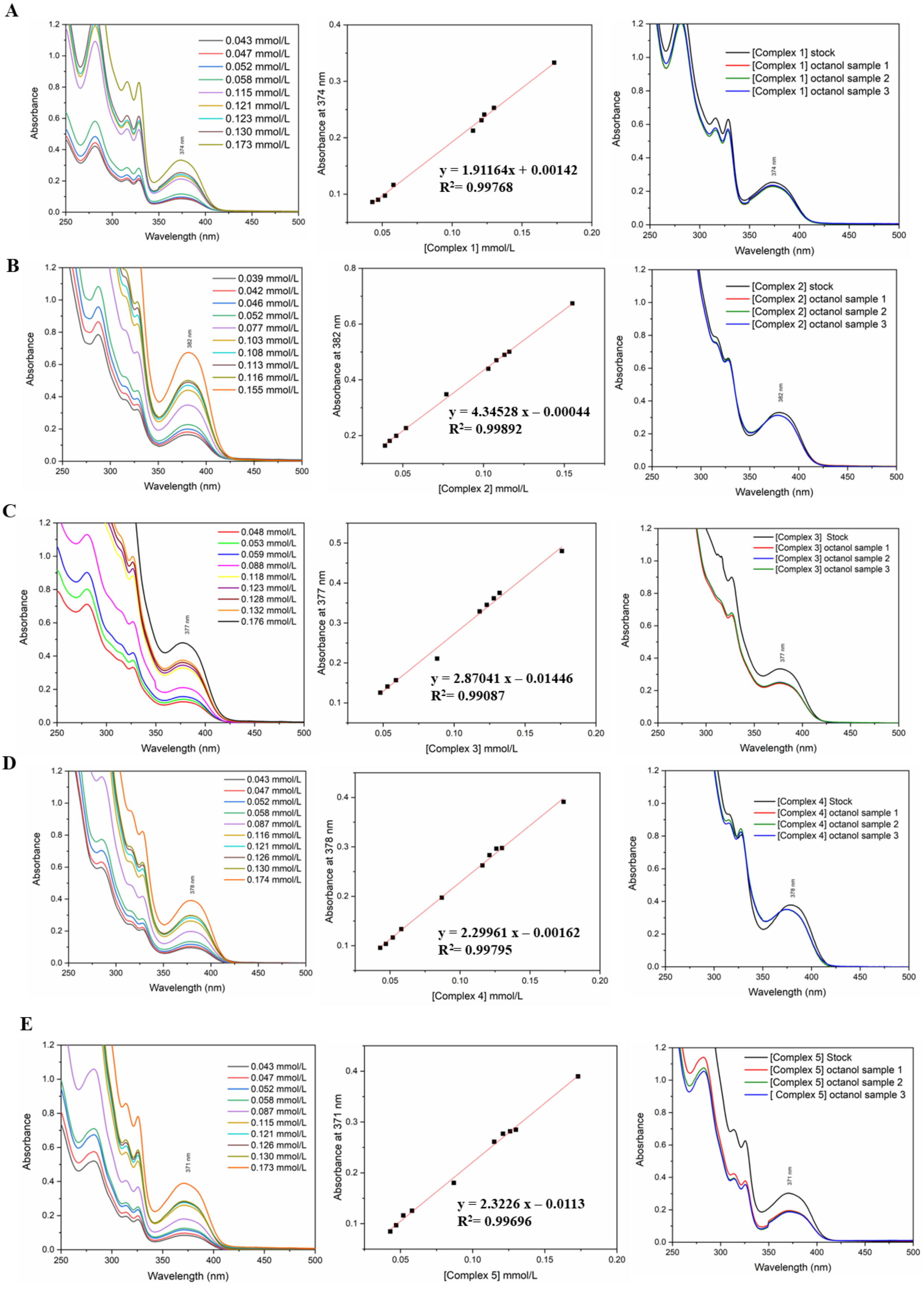


Figure S15: **A, D-E.** Calibration curve UV-Vis Complex 1,4 and 5, 1-Octanol/DMSO (95/5) and UV-vis spectra of the Stock Solution and Sample of Complex 1,4 and 5. **B-C.** Calibration curve UV-Vis Complex 2 and 3, 1-Octanol/DMSO (85/15) and UV-vis spectra of the Stock Solution and Sample of Complex 2 and 3.

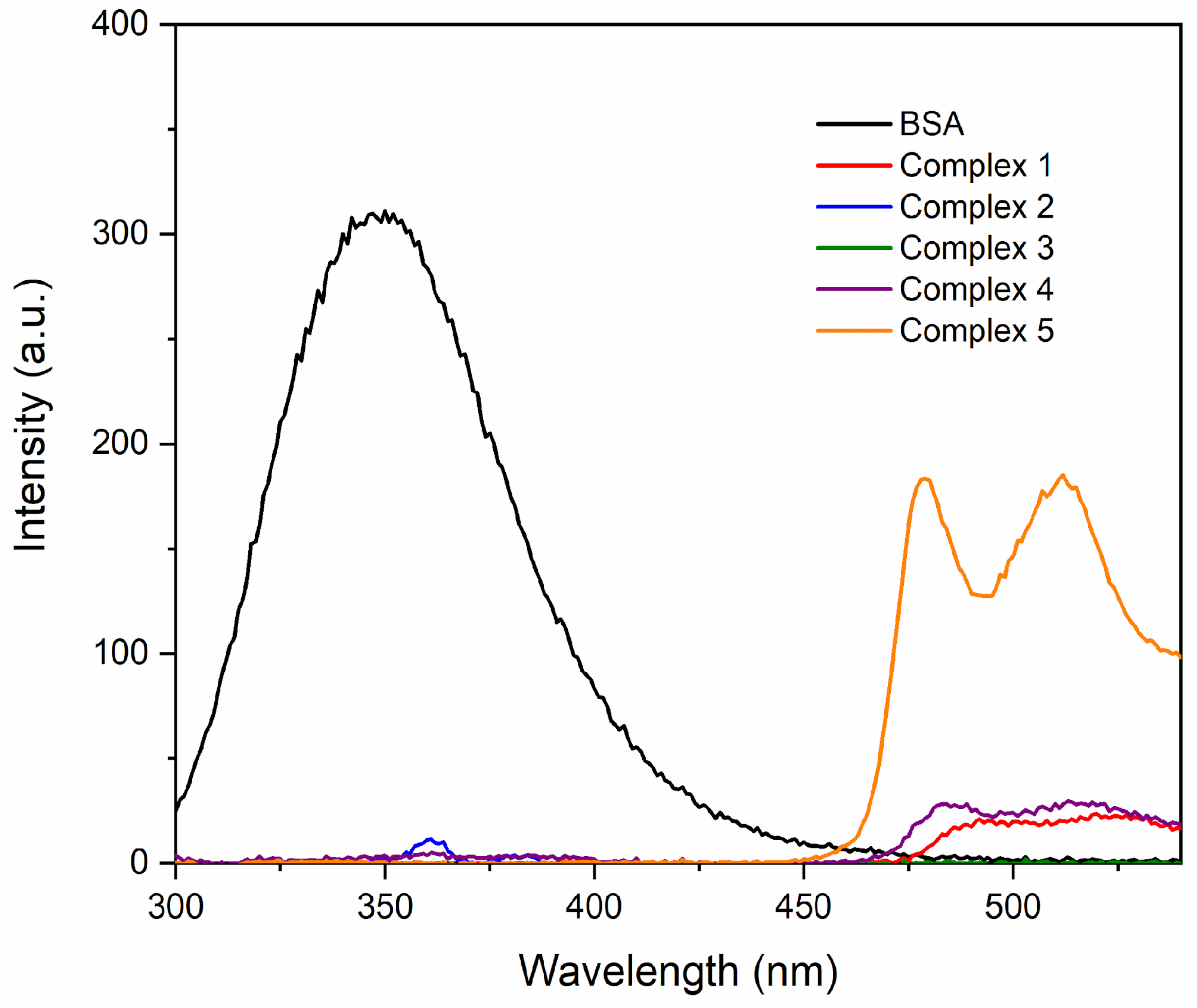


Figure S16: Emission spectra ($\lambda_{ex}$=280 nm) of 1.3 × $10^{-4}$ mol/L of Complex 1-5 and BSA.

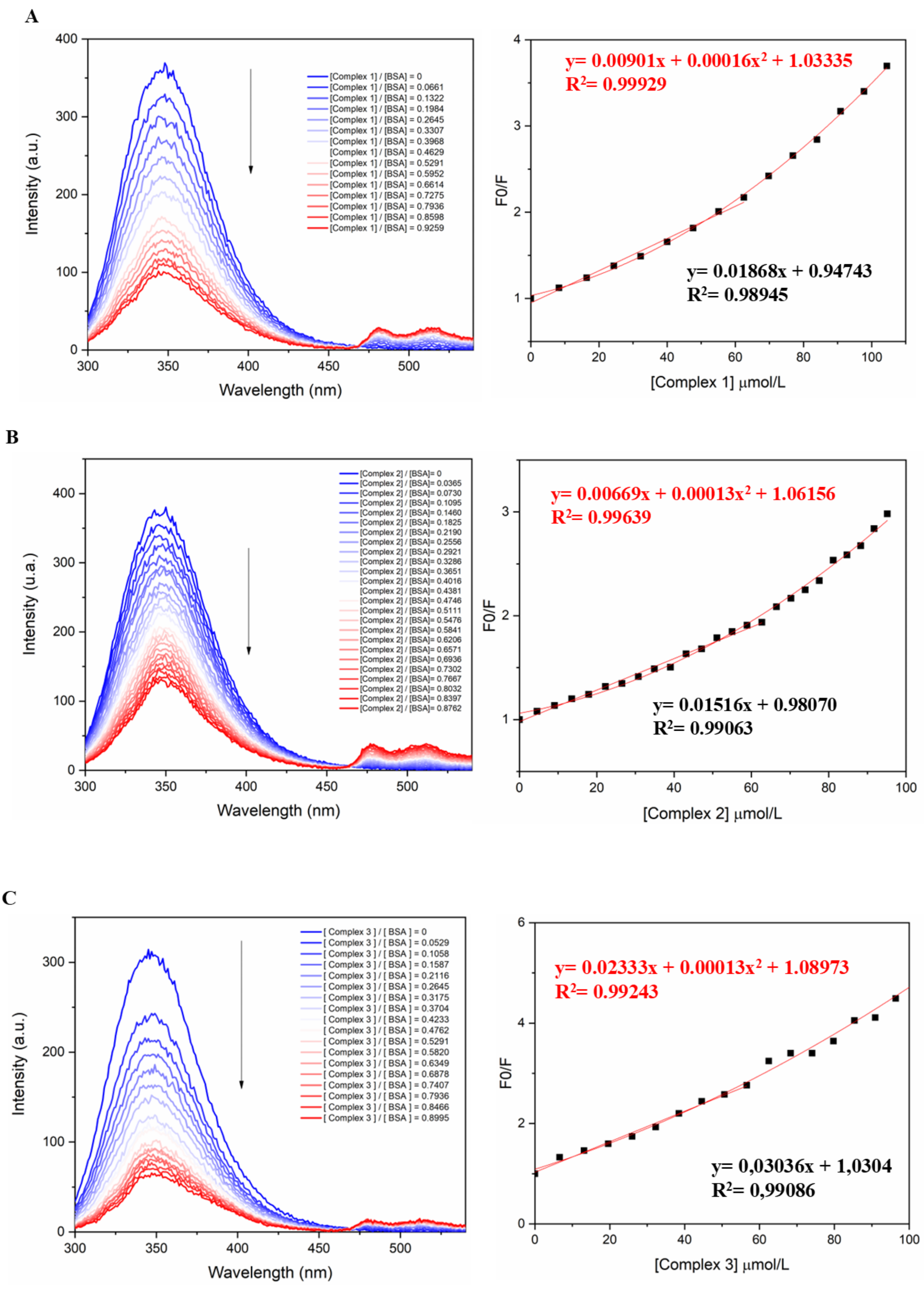


Figure S17: **A-C.** Titration of Complex 1-3 with BSA, the $F_0/F$ vs. [Complex 1-3] and equation of the complete polynomial curve, in red, and equation of the linear portion, in black, used to calculate $K_{SV}$ for each complex.

**A**

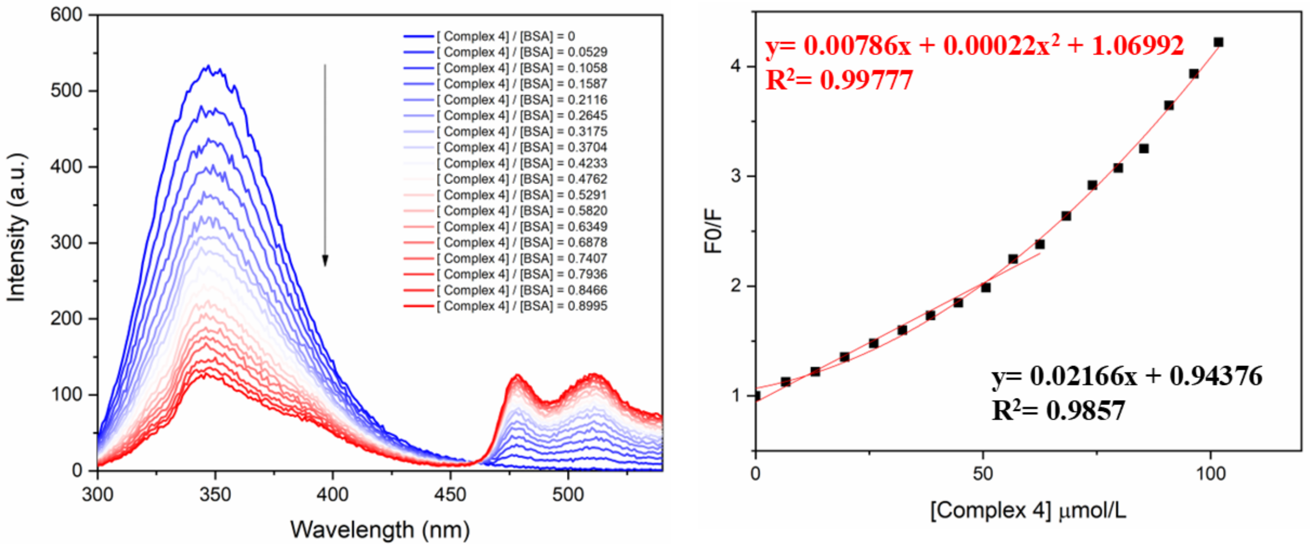


**B**

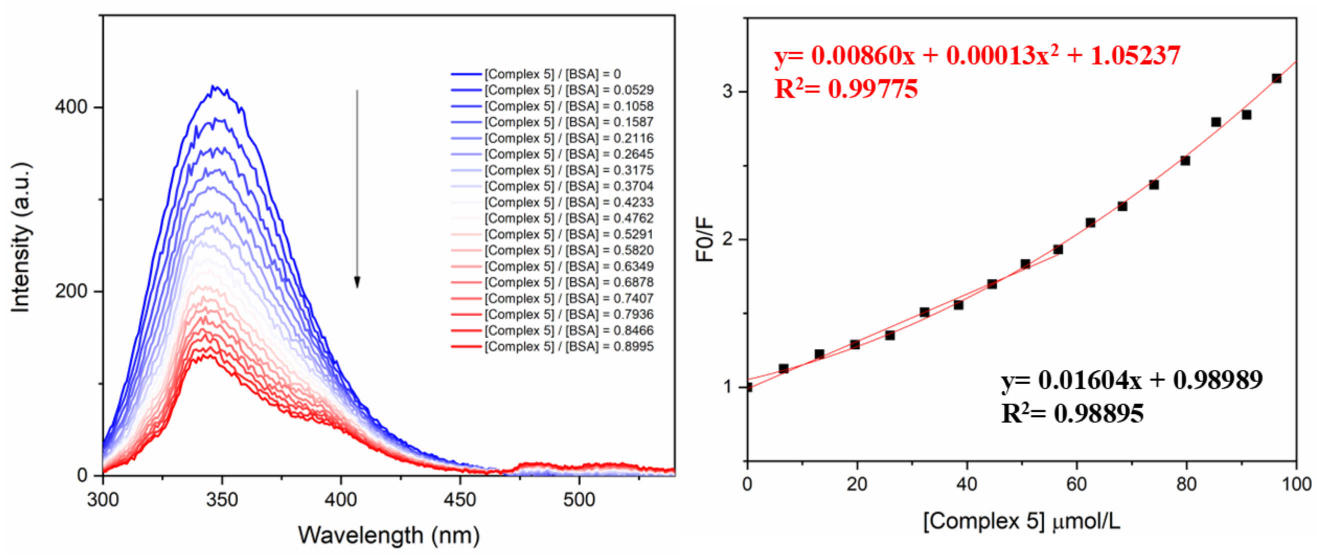


Figure S18: **A-B.** Titration of Complex 4-5 with BSA, the $F_0/F$ vs. [Complex 4-5] and equation of the complete polynomial curve, in red, and equation of the linear portion, in black, used to calculate $K_{SV}$ for each complex.

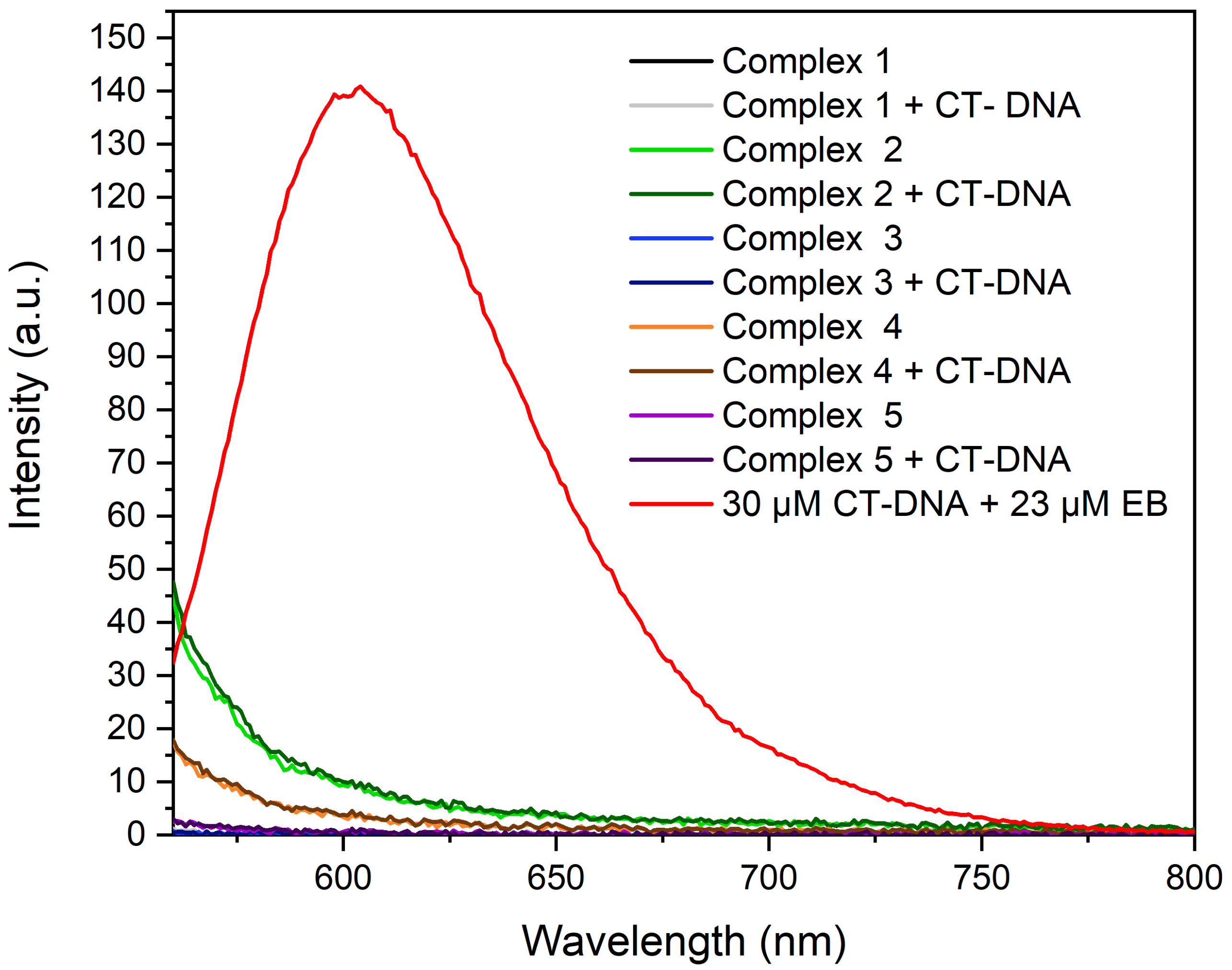


Figure S19: Emission spectra ($\lambda_{ex}$ = 540 nm) of 100 μM of Complexes 1–5, 100 μM of Complexes 1–5 + 30 μM of CT-DNA and 30 μM of CT-DNA + 23 μM of EB.

A

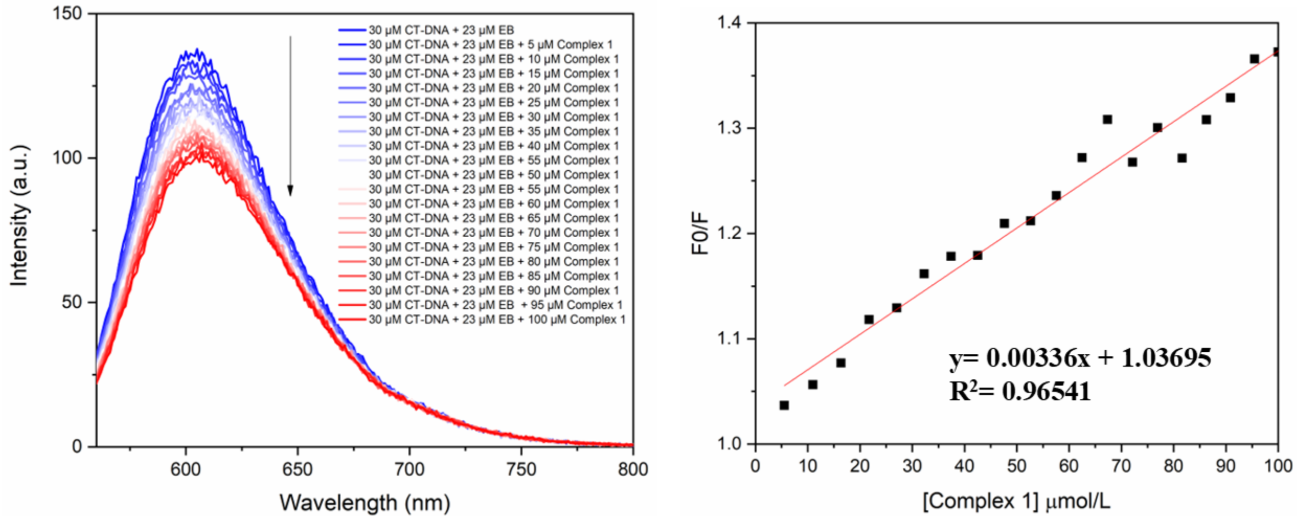


B

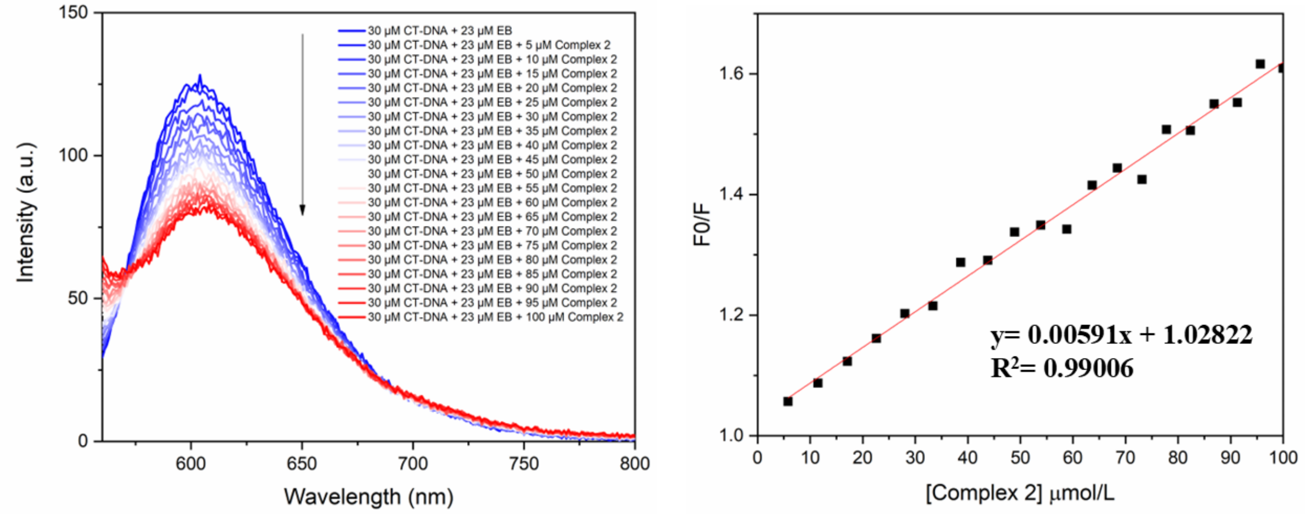


C

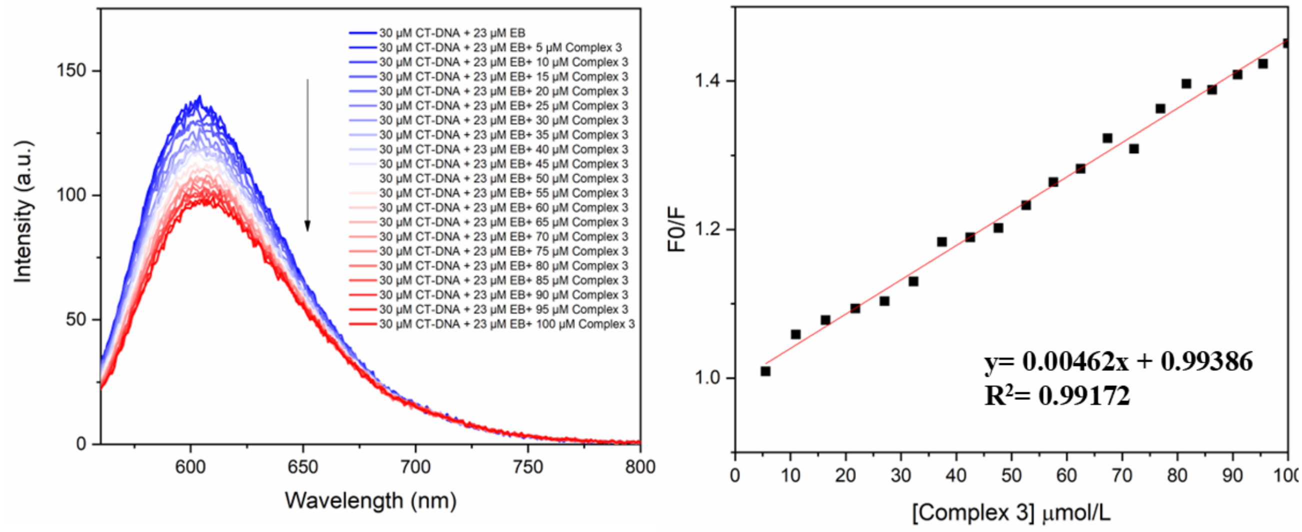


Figure S20: **A-C.** Titration of EB–CT-DNA with Complexes 1-3, the $F_0/F$ vs. [Complex 1-3] and equation used to calculate $K_{SV}$ and $k_q$ for each complex.

**A**

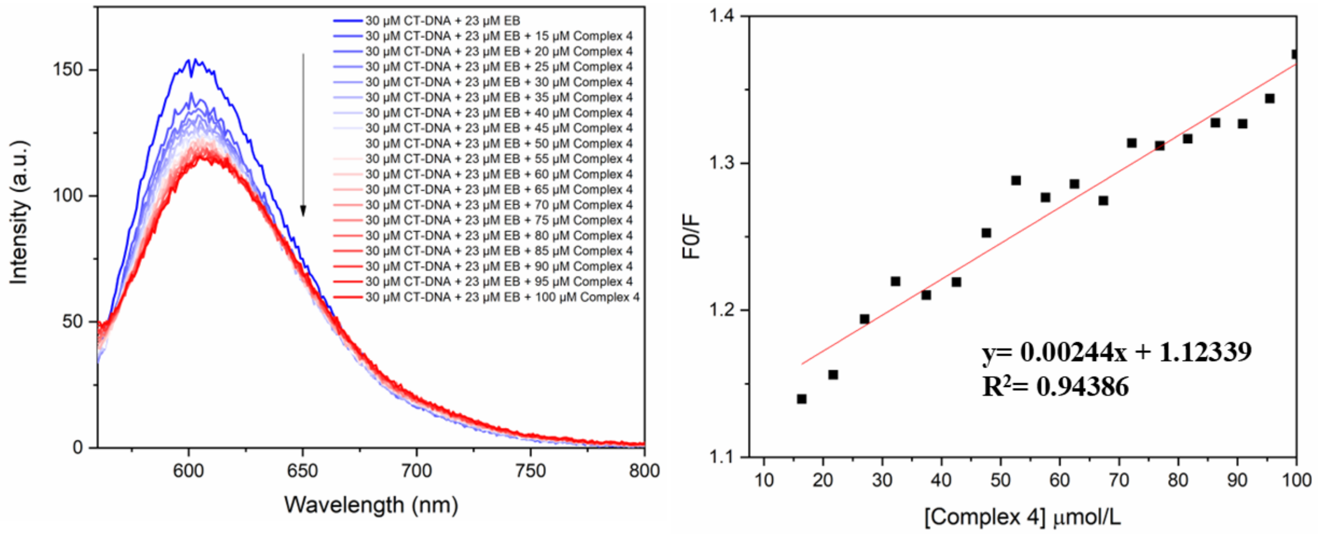


**B**

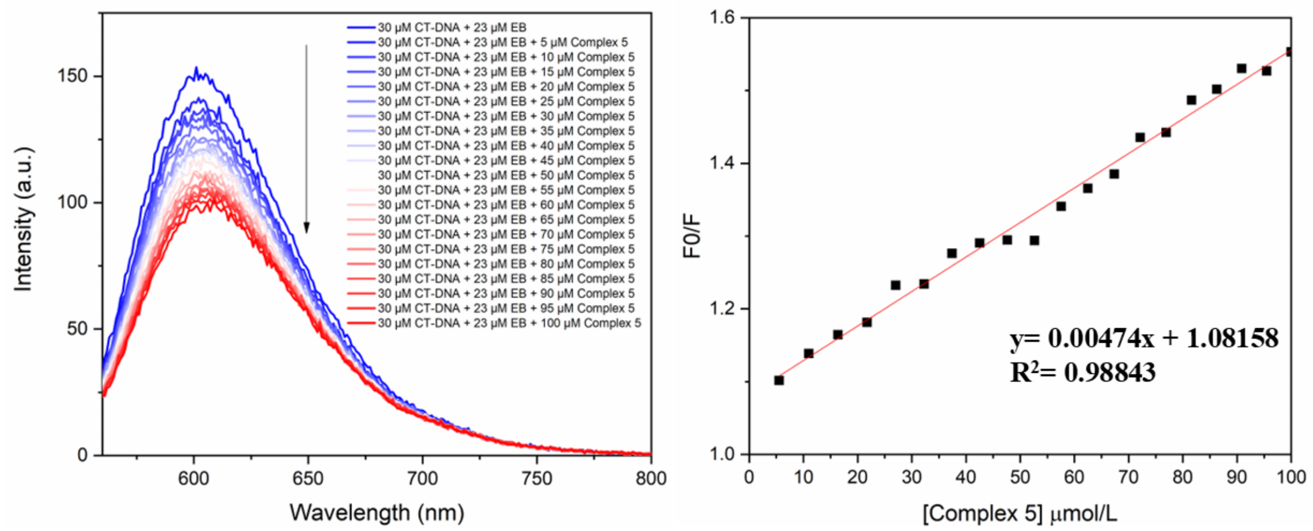


**C**

| Complex | ($\Delta F/F_0$, %) | n | $K_f$ $M^{-1}$ |
|---|---|---|---|
| **1** | 27.45 | 0.80 ± (0.02) | (8.97 ± 0.76) x $10^3$ |
| **2** | 37.50 | 0.86 ± (0.02) | (1.11 ± 0.70) x $10^4$ |
| **3** | 30.72 | 0.98 ± (0.03) | (4.79 ± 0.53) x $10^3$ |
| **4** | 27.27 | 0.63 ± (0.03) | (2.03 ± 0.25) x $10^4$ |
| **5** | 35.71 | 0.61 ± (0.02) | (3.10 ± 0.29) x $10^4$ |

Figure S21: **A-B.** Titration of EB–CT-DNA with Complexes 4-5, the $F_0/F$ vs. [Complex 4-5] and equation used to calculate $K_{SV}$ and $k_q$ for each complex. **C.** Percentage of fluorescence quenching of the EB–CT-DNA adduct, number of binding sites per nucleotide, and fluorescence binding constant (apparent binding constant) for the five complexes.

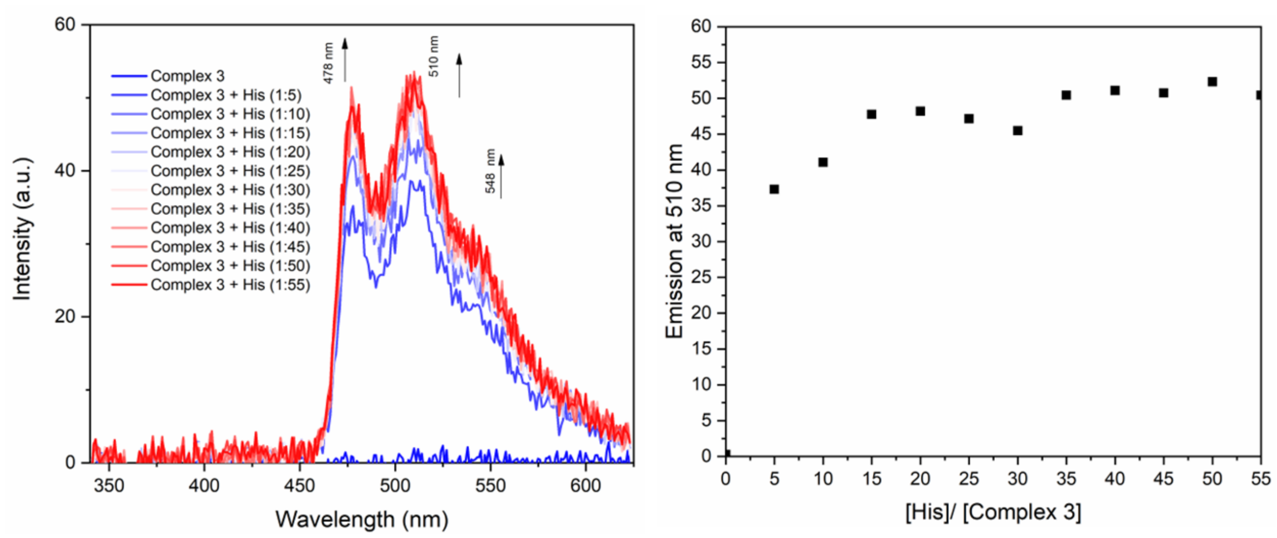


Figure S22: Fluorescence titration of Complex 3 with His, monitoring the emission intensity at 510 nm, in molar ratios from 1:5 to 1:55, with excitation at 321 nm.

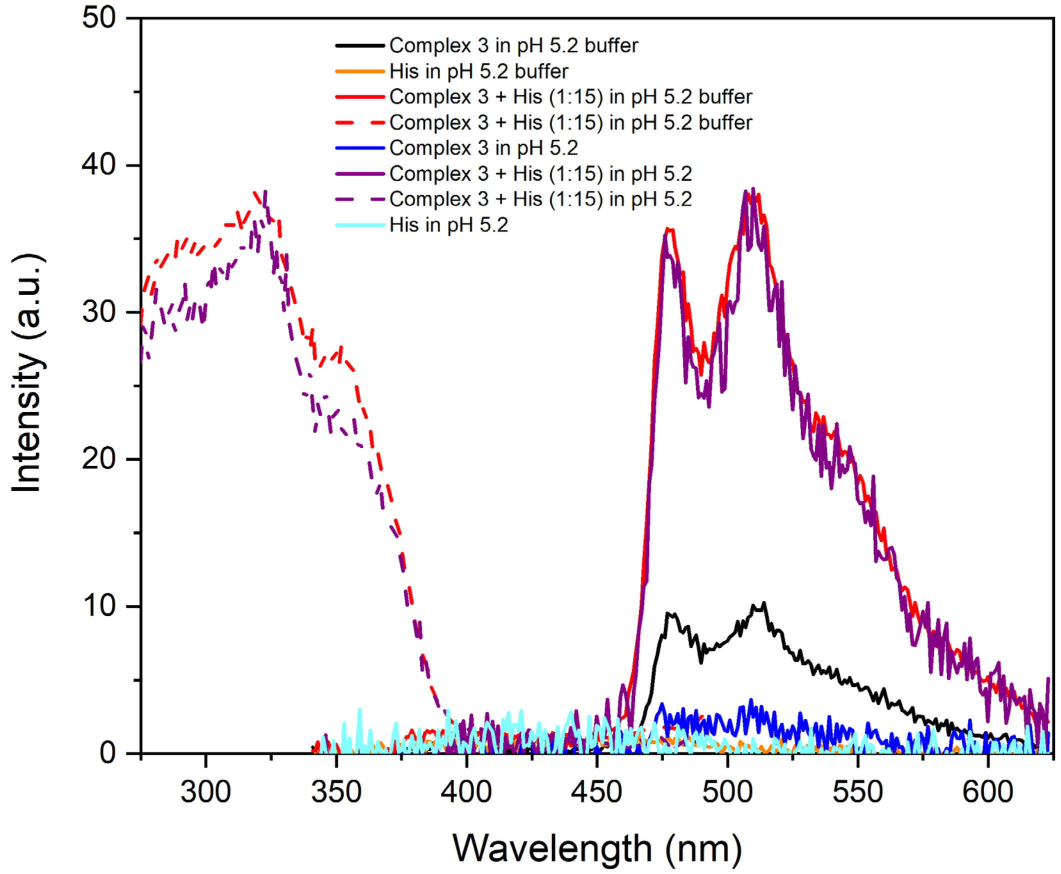


Figure S23: Excitation spectra ($\lambda_{em}$= 510 nm) dashed red for Complex 3 + His (1:15) in pH 5.2 buffer and dashed purple for Complex 3 + His (1:15) in pH 5.2. Emission spectra ($\lambda_{ex}$= 321 nm) solid black for Complex 3 in pH 5.2 buffer, solid Orange for His in pH 5.2 buffer, solid red for Complex 3 + His (1 :15) in pH 5.2 buffer, solid blue for Complex 3 in pH 5.2, solid purple for Complex 3 + His (1:15) in pH 5.2 and solid cyan for His in pH 5.2

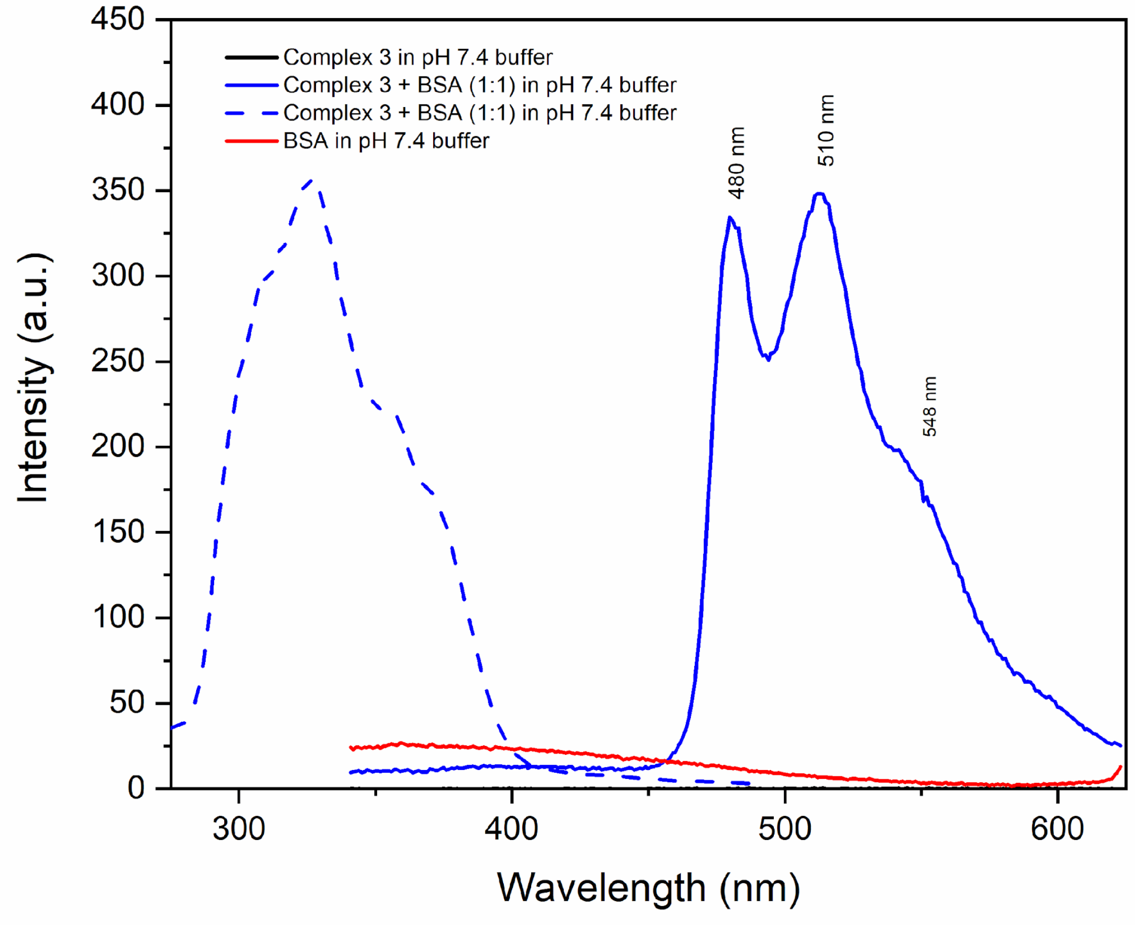


Figure S24: Excitation spectra ($\lambda_{em}$= 510 nm) dashed blue for Complex 3 + BSA (1 :1) in pH 7.4 buffer Emission spectra ($\lambda_{ex}$= 321 nm) solid blue for Complex 3 + BSA (1 :1) and solid red for BSA in pH 7.4 buffer.

## References


[1] S. R. Bhowmik, S. Gangopadhyay, P. K. Gangopadhyay, "Platinum coordination compounds of thiosemicarbazide derivatives: A new class of platinum blues" *J. Coord. Chem.* **2005**, *58*, 795–801.

[2] J. Ruiz, C. Vicente, C. De Haro, A. Espinosa, "Synthesis and antiproliferative activity of a C/N-cycloplatinated(II) complex with a potentially intercalative anthraquinone pendant" *Inorg. Chem.* **2011**, *50*, 2151–2158.

[3] M. Jamshidi, M. Babaghasabha, H. R. Shahsavari, S. M. Nabavizadeh, "The influence of thiolate ligands on the luminescence properties of cycloplatinated(II) complexes" *Dalt. Trans.* **2017**, *46*, 15919–15927.

[4] M. V. Dobrynin, E. V. Sokolova, M. A. Kinzhalov, A. S. Smirnov, G. L. Starova, V. Y. Kukushkin, R. M. Islamova, "Cyclometalated Platinum(II) Complexes Simultaneously Catalyze the Cross-Linking of Polysiloxanes and Function as Luminophores" *ACS Appl. Polym. Mater.* **2021**, *3*, 857–866.

[5] J. Hu, M. Nikravesh, H. R. Shahsavari, R. Babadi Aghakhanpour, A. L. Rheingold, M. Alshami, Y. Sakamaki, H. Beyzavi, "A C^N Cycloplatinated(II) Fluoride Complex: Photophysical Studies and Csp3-F Bond Formation" *Inorg. Chem.* **2020**, *59*, 16319–16327.

[6] M. A. Ivanov, M. V. Puzyk, "Preparation and optical properties of Au(III) phenylpyridynate complexes" *Russ. J. Gen. Chem.* **2001**, *71*, 1660–1661.
